# In-Field and Zero-Field Relaxation Dynamics of Dysprosocenium in Solution

**DOI:** 10.1021/acs.jpca.4c06678

**Published:** 2025-02-25

**Authors:** William J. A. Blackmore, Sophie C. Corner, Peter Evans, Gemma K. Gransbury, David P. Mills, Nicholas F. Chilton

**Affiliations:** † Department of Chemistry, School of Natural Sciences, University of Manchester, Oxford Road, Manchester M13 9PL, U.K.; ‡ Research School of Chemistry, The Australian National University, Building 137, Sullivans Creek Road, Canberra ACT 2601, Australia

## Abstract

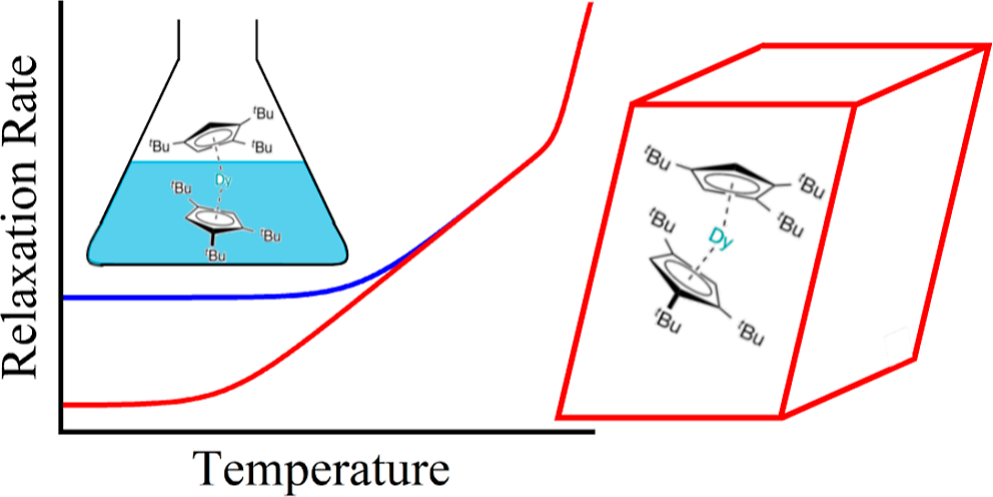

Most of the work in expanding the frontiers of single-molecule magnets employs the chemical design of new molecules to increase the size of the effective barrier (*U*_eff_) or the hysteresis temperature (*T*_H_). Here we explore how perturbing the local environment affects magnetic relaxation properties by dissolving [Dy(Cp^ttt^)_2_][B(C_6_F_5_)_4_] in two different solvents: difluorobenzene (DFB) and dichloromethane (DCM). Surprisingly, we find no significant effects in the phonon-driven Raman-I regime at higher temperatures, but we do observe that the frozen-solution environment increases the rate of quantum tunneling of the magnetization (QTM) due to an increase in the size of the avoided level crossing. We find that there is a drastic decrease in the Raman relaxation rate at low temperatures for the concentrated DCM and polycrystalline samples under the applied magnetic field where the QTM process is quenched, which is attributed to changes in the low-energy phonon spectrum and is not replicated for the other samples.

## Introduction

Single-molecule magnets (SMMs) are highly anisotropic superparamagnetic molecules that have large energy barriers that impede the reversal, or relaxation, of their magnetic moment.^1^ At cryogenic temperatures, the scarcity of phonons means that magnetization reversal is sufficiently slow such that the moment is often considered blocked on the time scale of the measurements and hence can show memory effects.^2,3^ Significant advancements in synthetic organometallic chemistry have recently led to vast increases in the magnitude of this barrier, raising the temperature at which memory effects can be observed. Currently, the most successful SMMs are based on Dy(III) ions with two bulky axial anionic ligands, generating a highly axial electrostatic potential and hence large magnetic anisotropy.^4−14^

Magnetic relaxation in SMMs (that is, the reversal of the total angular momentum) arises due to coupling between the spin and phonon degrees of freedom that facilitates absorption/emission/scattering of the latter to generate transitions of the former.^3,15^ Most of the focus in this area has concentrated on increasing the temperature at which these memory effects are observed through the chemical design of larger magnetic anisotropy in new molecules.^4−14^ Other chemical design trends look at increasing the QTM relaxation time at the lowest temperatures using magnetic exchange, incorporating radical^16^ and dimetallic complexes.^17,18^ Other routes include using increasing symmetry^19^ or isotopes^20^ to suppress QTM, and radicals are known to shift the QTM resonance away from zero field.^21^ Local fields can also be controlled by the design of the packing of crystal structures.^22^

However, there has been comparatively little work in understanding how relaxation processes are affected by extrinsic local environmental effects that could potentially modulate the phonon spectrum presented to the molecule.^23−25^ Recently, we showed that dissolving [Dy(Cp^ttt^)_2_][B(C_6_F_5_)_4_] in difluorobenzene (DFB) and dichloromethane (DCM) surprisingly increased the size of the tunneling gap compared to the polycrystalline sample.^26^ This was despite a decrease in the dipolar field, which is one of the processes that facilitates QTM. The size of the transverse interaction that gives rise to the tunneling gap is related to the QTM rate via the coherence time. However, the coherence time is also a function of the transverse interaction, and hence, it is not possible to quantify or estimate the transverse interaction by solely comparing QTM rates. We attributed the increase in the tunneling to some form of structural or packing change brought on by the frozen solvent environment versus the crystalline phase. In an attempt to elucidate further the underlying processes, we extend our work herein to look at the temperature- and field-dependent relaxation rates of the same [Dy(Cp^ttt^)_2_][B(C_6_F_5_)_4_] samples. We find that a solution environment increases the rate of the relaxation of [Dy(Cp^ttt^)_2_][B(C_6_F_5_)_4_] in the QTM regime and that there is a nontrivial dependence on the low-energy phonon spectrum such that the phonon-pair-driven Raman-I relaxation tends toward the *T*^2^ limit as a function of solvent and solute concentration.

## Experimental Details

A polycrystalline sample of [Dy(Cp^ttt^)_2_][B(C_6_F_5_)_4_] was prepared,^27^ and 27.5 mg was pulverized in a mortar and pestle and then secured inside a 5 mm flame-sealed NMR tube using 20.3 mg of eicosane. Solution samples were prepared under an inert argon atmosphere through the serial dilution of a 200 mM stock solution of [Dy(Cp^ttt^)_2_][B(C_6_F_5_)_4_] (52.3 mg, 0.04 mmol) in DFB or DCM (200 μL). Owing to sample instability in DCM, the solutions were kept below 0 °C throughout. 100 μL of each solution (DFB: 200 mM, 100 mM, and 10 mM; DCM: 200 mM and 100 mM) was pipetted into borosilicate NMR tubes, frozen by immersion in liquid nitrogen, placed under vacuum, and flame-sealed to a length of ≈3 cm. We can be confident that the samples remain chemically stable following this protocol: decomposition of this complex is accompanied by a diagnostic color change from yellow to pink (not observed here) and that the decomposition product shows no slow magnetization dynamics (cf. the significant remnant magnetization observed here). We note here that we prepared a sample with 10 mM [Dy(Cp^ttt^)_2_][B(C_6_F_5_)_4_] in DCM but observed no slow magnetization dynamics, so this sample was not pursued further. N.B.: these are the same samples used by Blackmore et al.^26^

Magnetic measurements were performed by using a Quantum Design MPMS3 SQUID magnetometer. Borosilicate NMR tubes containing the samples were loaded into straws that were fixed to a glass-reinforced polycarbonate adaptor attached to a carbon–fiber rod. DC decay measurements were performed for all samples using DC mode, with the sample saturated at 3 T for 20 min before the field was dropped to target at 700 Oe/s. The field was stabilized before measurement of the moment began. We have used the “DC Moment Free Ctr (emu)” values of the moment in the analysis of the decay measurements. The resultant magnetization decays were fitted to a stretched exponential function of the form
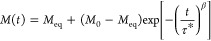
1with *M*_0_ fixed to the moment at the first measured point. In the case of zero-field decays, *M*_eq_ was initially left as a free variable (see the Supporting Information). At higher temperatures where the sample has almost completely equilibrated, the *M*_eq_ data can be modeled using
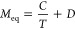
2where *C* is the Curie constant and *D* is a temperature-independent diamagnetic contribution. This relationship was extrapolated to lower temperatures, allowing us to fix *M*_eq_ for all of the presented zero-field decays. The *M*_eq_ for infield decay measurements were calculated from calibrated PHI simulations of the magnetization using the crystal field parameters calculated by Goodwin et al.^4^ as the decays take too long to feasibly measure the equilibrium values directly.

All fitting of the DC decay curves and relaxation profiles were performed using CCFIT2.^28,29^

## Results and Discussion

### Temperature-Dependent Magnetization Dynamics in Zero Field

Magnetization decay measurements of solution samples of [Dy(Cp^ttt^)_2_][B(C_6_F_5_)_4_] were performed in zero DC field between 1.8 and ≈60 K (Figures S11–S31), giving associated magnetic relaxation rates (Figure 1). At higher temperatures, the data show very little concentration and solution dependence, with a small distribution of rates. As the temperature is lowered into a regime dominated by QTM, there is a large increase in the width of the rate distribution due to the frozen amorphous nature of the sample leading to a wide distribution of perturbed [Dy(Cp^ttt^)_2_]^+^ moieties. Furthermore, there is a small divergence in the solution rates; the fastest is 200 mM in DCM and the slowest is 10 mM in DFB.

**Figure 1:**
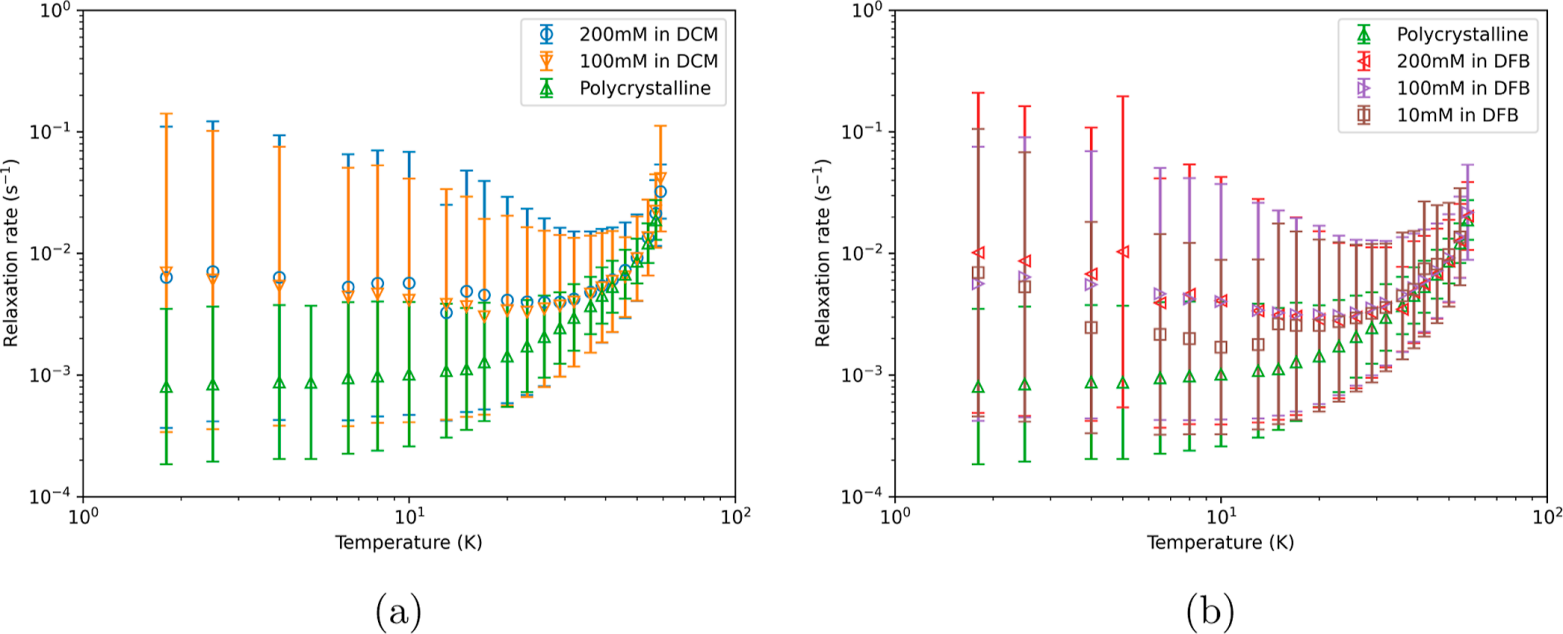
Relaxation profile for zero-field field decay measurements of the polycrystalline and DCM samples (a) and DFB samples (b). *T* > 5 K data for the polycrystalline sample taken from Parmar et al.^27^ and remodeled.

The polycrystalline sample shows significantly slower rates, about an order of magnitude slower than the solution samples, and has a narrower distribution of rates. A slower rate for the polycrystalline sample in the QTM regime is unexpected as separation of magnetic centers via dilution should decrease dipolar interactions and hence suppress QTM. However, previous work has shown that dissolving [Dy(Cp^ttt^)_2_][B(C_6_F_5_)_4_] in DFB and DCM increases the size of the tunneling gap,^26^ which indeed supports an increase in the QTM rates, as observed here. This can be rationalized via likely distortions to the linearity of the [Dy(Cp^ttt^)_2_][B(C_6_F_5_)_4_] motif in frozen solutions and/or close equatorial contacts with solvent molecules disrupting the axiality of the crystal field, thus increasing mixing of |*m*_J_| < 15/2 into the ground state wave function and making the molecules more susceptible to opening of a QTM tunneling gap via transverse dipolar fields compared to the solid-state sample.

At the lowest temperatures, there is a slight rise in the “central”  values^29^ for the solution samples in the QTM regime. We find that the lower bound of the one-σ estimated standard deviation of the distribution of rates remains constant but that the upper one-σ bound increases. We attribute this rise to the appearance of more complicated decay curves that cannot be captured by a stretched exponential model (Figures S11–S31). Adding additional stretched exponential functions to the model makes the fits worse. There is no physical reason why magnetic relaxation rates should increase with decreasing temperature, and as such, this must be viewed as an artifact of using a simple model to interpret the collective behavior of a nonuniform amorphous ensemble.

**Figure 2:**
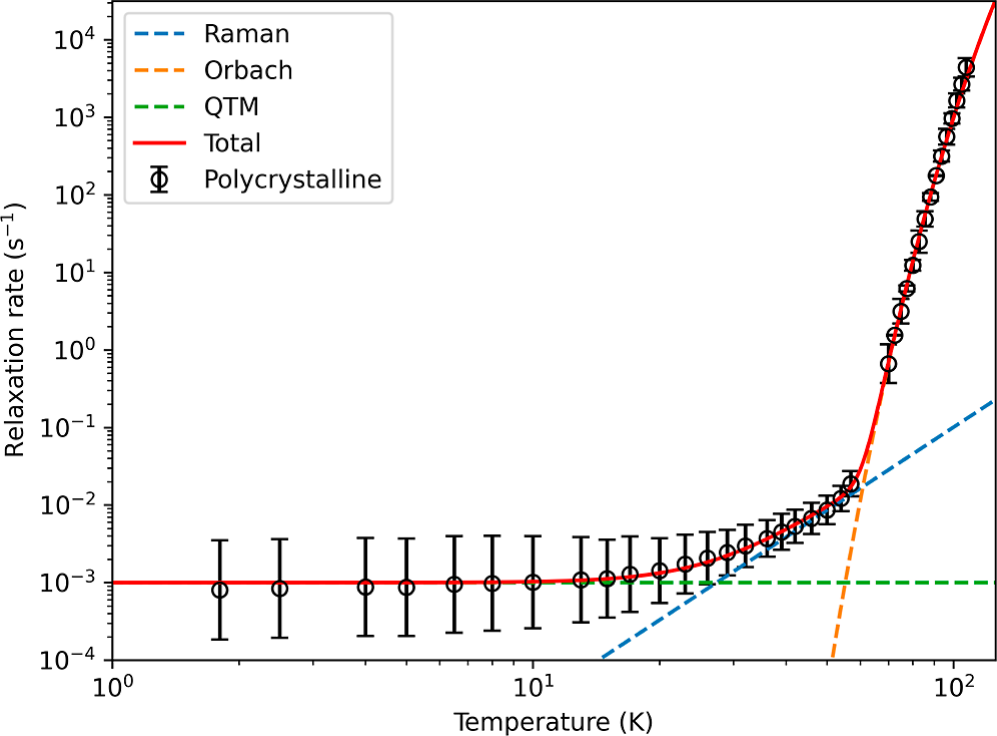
Relaxation profile of polycrystalline [Dy(Cp^ttt^)_2_][B(C_6_F_5_)_4_] in zero-field fitted to eq 3.

The zero-field temperature-dependent magnetic relaxation rates of the polycrystalline crystalline sample have been previously reported by Parmar et al.^27^ However, an updated protocol for analyzing magnetometry data has since been developed, providing greater accuracy and consistency with DC decay measurements.^29^ Thus, we have reanalyzed these data^27^ using the new protocol and performed additional low-temperature DC decay measurements to the same low-temperature range as we have investigated for the frozen solution samples. The temperature-dependent relaxation data was fitted using eq 3
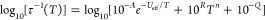
3with Orbach (10^–*A*^e^–*U*_eff_/*T*^), Raman (10^*R*^T^*n*^), and QTM (10^–*Q*^) contributions to the relaxation rate. The resultant fit (Figure 2) gives , *U*_eff_ = 1706(4) K, , *n* = 3.56(59), and  in good agreement with the previous parameterization.^27^ We attempted to fit the regime between the exponential Orbach and temperature-independent QTM regimes with the phonon-pair-driven (PPD) Raman model;^30^ however, this gave a slightly worse fit than the common power-law Raman expression which gave the best agreement to experimental data (Figure S32).

Due to the limited temperature regime where the Raman process dominates in the frozen solution samples, freely fitting eq 3 leads to a nonphysical Raman exponent of *n* > 10. We therefore fix the value of *n* ≡ 3.56 from the modeling of the polycrystalline sample, along with the Orbach parameters, while the *R* and *Q* parameters are fitted (Figure S33 and Table 1); note that the low-temperature data where the rates are increasing were not included, and the temperature ranges at which fits were performed are shown in Table 1. The Raman parameter *R* shows little solution dependence, whereas the QTM rate is noticeably faster for the solution samples compared to that for the polycrystalline sample.

**Table 1:** Parameters from Fitting Eq 3 to Temperature-Dependent Relaxation Data of Polycrystalline and Solution Samples of [Dy(Cp^ttt^)_2_][B(C_6_F_5_)_4_]^a^

sample	*U*_eff_ K	*A* Log[s]	*n^a^*	*R* Log[s^–1^K^–*n*^]	*Q* Log[s]	*T* range K
polycrystalline	1706(4)	–10.40(2)	3.56(59)	–8.1(1.0)	3.00(10)	1.8–57
200 mM in DFB	1706	–10.40	3.56	–8.12(1)	2.99(2)	20–57
100 mM in DFB	1706	–10.40	3.56	–8.19(6)	2.55(7)	13–57
10 mM in DFB	1706	–10.40	3.56	–8.19(3)	2.58(3)	15–57
200 mM in DCM	1706	–10.40	3.56	–8.19(5)	2.50(6)	15–57
100 mM in DCM	1706	–10.40	3.56	–8.16(5)	2.45(6)	13–57

a The Raman parameter *n* ≡ 3.56 was fixed for the solution samples (see main text). Parentheses indicate the distribution of the parameters on the last significant figure. The far right column indicates the temperature ranges that were fitted to eq 3.

### Field-Dependent Magnetization Dynamics

**Figure 3:**
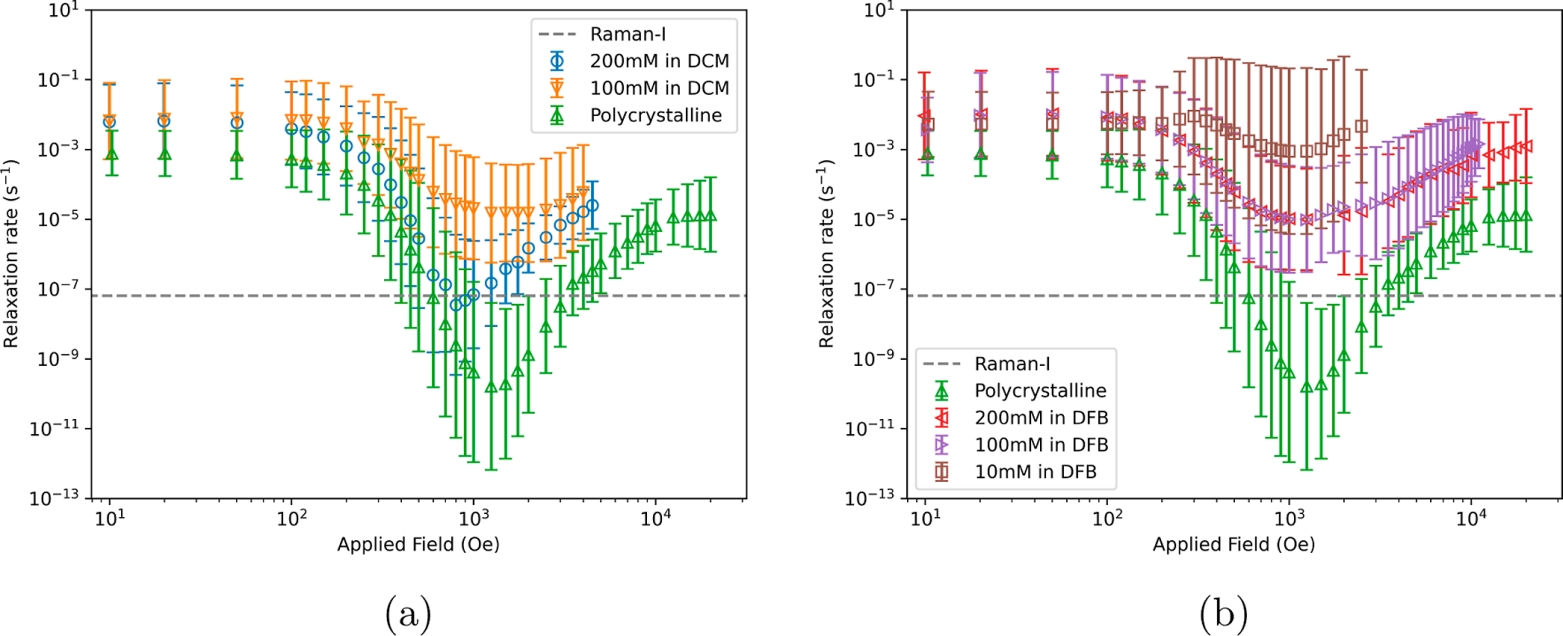
Relaxation rates obtained from in-field DC decay measurements of the polycrystalline, DCM (a), and DFB (b) samples, at 1.8 K. The temperature-dependent Raman-I fit to the polycrystalline sample (Table 1) has been extrapolated to 1.8 K and is shown here as the dark-gray dashed line.

DC decay measurements of polycrystalline and frozen solution samples of [Dy(Cp^ttt^)_2_][B(C_6_F_5_)_4_] were performed at 1.8 K in nonzero magnetic fields between 0 and 2500–20000 Oe (Figures S37–S73) and fitted with stretched exponential models to extract the magnetic relaxation rates (Figure 3). The frozen solution samples all show very similar relaxation rates at small fields, while the polycrystalline sample shows slower relaxation, consistent with the zero-field measurements. As the magnetic field increases, the QTM mechanism can be quenched, and hence, the rates generally become slower. We observe that the 200 mM sample in DCM becomes slower and the 10 mM in DFB remains faster compared to the other solution samples; the polycrystalline sample becomes much slower than all frozen solution samples. There is a minimum in all data sets around 1000 Oe which is a crossover from a field-dependent quenching of the QTM region to a field-dependent Raman-II or direct regime.^31^ The value of the relaxation rate at the minimum is considered to be an upper bound on the field-independent Raman-I process;^32^ however, for the polycrystalline and 200 mM in DCM samples, the minimum is very sharp, suggesting that the field-independent Raman-I process is much slower. Conversely, the 100 mM in DCM, 100 mM in DFB, and 200 mM in DFB samples all have similar rates at the minimum that appear to be quite flat, suggesting they may be limited by the field-independent Raman-I process in this regime. The outlier is the 10 mM in DFB sample showing relaxation rates that are 2 orders of magnitude faster than the next slowest solution samples as well as an increase of the upper 1-σ limit even as the expectation value of the relaxation rates drops to the crossover point.

**Figure 4:**
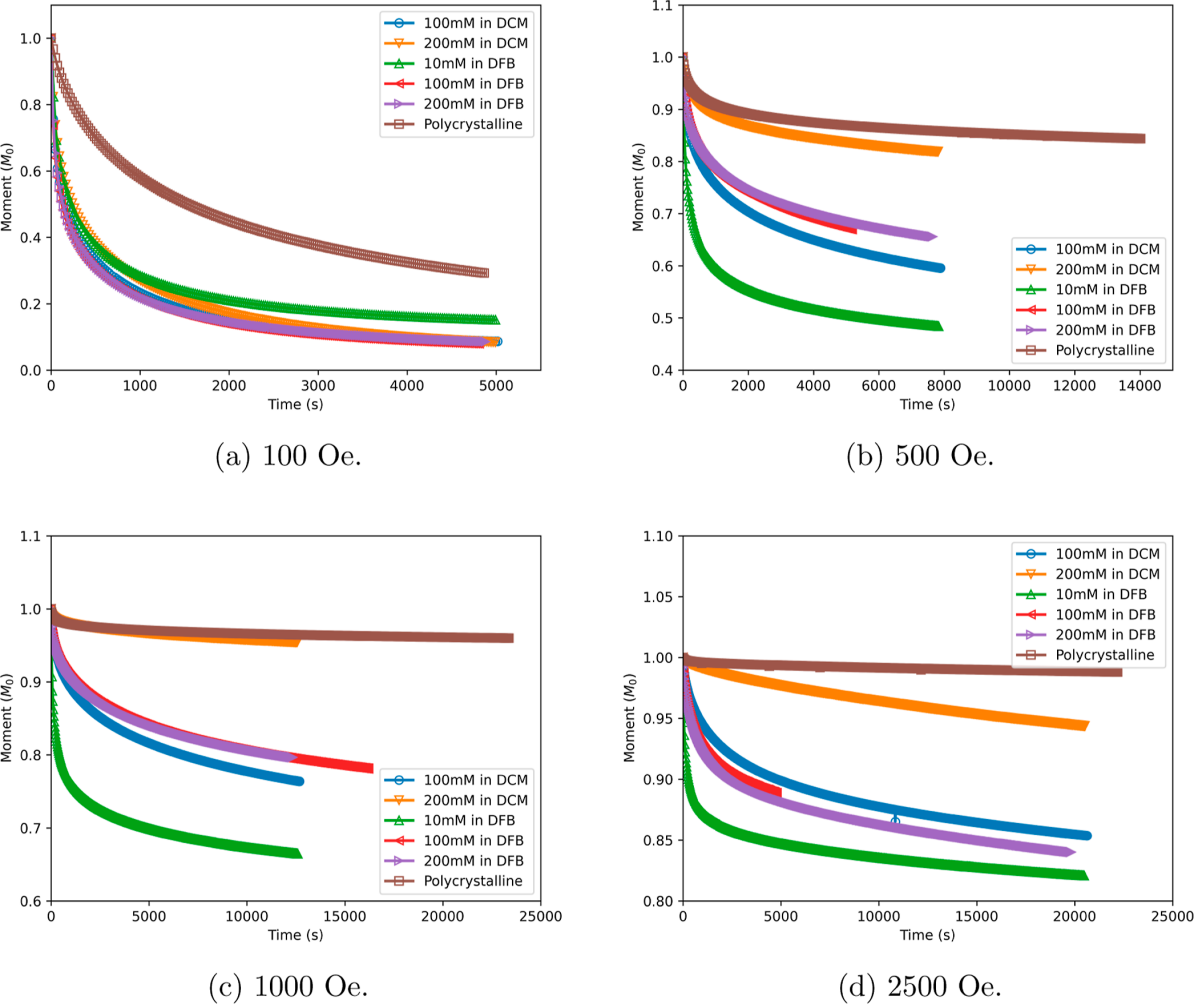
Decay profile for all samples at an applied field of (a) 100, (b) 500, (c) 1000, and (d) 2500 Oe. Each data set has been normalized to the moment of the first measured point of the decay.

As the magnetic field is increased further, all samples show an increase in their relaxation rates at a similar gradient (note the log_10_–log_10_ scale), indicating that a similar field-dependent process begins to dominate in this regime. We note, however, that the slowest relaxation times indicated here are of the order of 300 years, which is obtained from a measurement lasting less than 7 h, and thus cannot be fully representative of the true relaxation time scale. While this urges some caution to the absolute values of the rates, we believe the trends across different samples are accurate. This can be observed by directly comparing the DC decay curves at 100, 500, 1000, and 2500 Oe between the different samples (Figure 4). These decay profiles show the same trends that are observed in Figure 3, with the polycrystalline sample clearly taking much longer to decay compared to that of the 10 mM in DFB sample, for example. Due to this limitation on the utility of the absolute values of magnetic relaxation, we do not fit these relaxation rate data to field-dependent expressions and will rather discuss the trends and effects of the frozen solution phase versus the crystalline phase.

## Discussion

The temperature-dependent relaxation data shows that the polycrystalline sample has very similar magnetization dynamics compared to the frozen solution samples in the intermediate temperature regime (30–60 K) where the Raman-I mechanism dominates. This is in agreement with the calculations by Staab et al.^33^ that showed that the extrinsic effect of a frozen solution phase phonon spectrum has little impact on the Raman-I spin dynamics compared to the polycrystalline compound. At the lowest temperatures, there is an increase in the relaxation rate for all of the solution samples compared to the polycrystalline compound. This is unexpected within the QTM regime, where increasing the distance between magnetic centers should decrease dipolar interactions and therefore suppress QTM. Our previous work on measuring the intrinsic QTM tunneling gap under the same conditions shows that while 100 and 200 mM samples have dipolar fields of a similar order of magnitude to the polycrystalline sample, the observed tunneling gaps are larger and that while the 10 mM sample has a near two orders-of-magnitude smaller dipolar fields, it has a similar tunneling gap to the polycrystalline sample.^26^ This change in the size of the tunneling gap, and therefore the QTM rate, was presumed to be driven by a change in the local structure that may, for example, involve a decrease in the Cp_cent_^R^-Dy-Cp_cent_^R^ angle due to a lack of crystal packing effects or the close approach of solvent molecules.^34,35^ Such changes would increase mixing of the smaller magnitude *m*_J_ functions into the ground state, thus increasing sensitivity toward transverse dipolar files in opening up a tunneling gap and thus explaining the increase in QTM rates measured here for the concentrated solution samples compared to the polycrystalline compound. However, we found here that the zero-field QTM rate for the 10 mM in DFB is similar to the other solution samples, while it appears to have field-dependent relaxation behavior that is significantly different (faster, less field dependence) compared to all other samples; indeed, the shapes of the partial hysteresis loops of this sample were also vastly different to all other samples.^26^ Clearly, the field-dependent spin dynamics in this most-dilute sample are different from the others, which at this point we cannot explain. It could be that there are nontrivial many-body effects or some aggregation effect that becomes apparent in low concentrations, in conjunction with a randomly dispersed sample with variations in the local structure that make any theoretical interpretation of these data beyond our current models of SMMs.

Regarding the minimum rates that occur around 1000 Oe as a function of the magnetic field, we can extrapolate our field-independent Raman-I model from the polycrystalline sample and compare the predicted rates to the minima for all compounds (Figure S34, dashed lines). This indicates that models derived from zero-field temperature-dependent data alone are insufficient to explain Raman-I relaxation at low temperatures. The extrapolated value is slower for the 100 mM in DCM and 100 and 200 mM in DFB solutions than the experimental data; however, the flat region at the base of the trough and the similarity of the rates for the 100 mM in DCM and 100 and 200 mM in DFB solutions in this region suggests that they are all rate-limited by the Raman-I process.^31,32^ For these samples, we can use the field-dependent data to improve the modeling of the Raman-I parameters. Performing a global fit of the temperature-dependent data with an additional point at 1.8 K taken from the minimum rate of the field-dependent data, we obtain updated Raman-I and QTM parameters (Figure S35 and Table S1), finding that *n* ∼ 2.2 and *R* ∼ 10^–5^ log_10_ [s^–1^ K^–*n*^] for all three solution samples within error of each other.

The sharp drop in the field-dependent rates for the polycrystalline and 200 mM in DCM samples can be accounted for using the PPD description of Raman-I relaxation. The PPD Raman model (derived by Briganti et al. using the Fourier transform of the two-phonon correlation function^30^) is an alternative expression for the Raman-I process that places significant importance on a low-energy Γ-point optical phonon mode, eq 4.
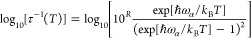
4where ω_α_ is the order of the first Γ-point optical modes. We have thus attempted to use this model instead of the standard power-law expression in eq 3 to reanalyze the field-dependent relaxation dynamics. We noted above that this model is a worse fit compared to the power-law Raman-I relaxation at higher temperatures for the polycrystalline sample (Figure S32). The other samples suffer from the same issue as the power-law Raman-I model, giving nonphysical values when fitting only the temperature-dependent data. However, if we include the position of the trough from the field-dependent measurements when modeling the temperature-dependent dynamics, we observe that the drastic decrease in the relaxation rate for the polycrystalline and 200 mM in DCM samples can be described using the PPD Raman model (Figure S36), with phonon energies of ω_α_ = 31.2(3.6) K and ω_α_ = 18.8(3.3) K, respectively (Table S2). For the 100 mM in DCM and 100 mM and 200 mM in DFB samples, the data can be modeled either using the PPD Raman or power-law expressions, Figure S35. This can be attributed to the first Γ-point optical modes having much lower energy in these samples, as in the limit *k*_B_*T* ≫ ω_α_, eq 4 simplifies to τ^–1^ ∝ *T*^2^.^30^ This is consistent with what we found when fitting these data sets to the power law model, where *n* ≈ 2.2 (Figure S35 and Table S1). While it is perfectly feasible to say that the phonon modes that drive the field-independent Raman-I process at low temperatures for the polycrystalline sample are at much higher energy than the other samples, for this to be also true for the 200 mM in DCM sample implies a nontrivial relationship between the host lattice and solute concentration in amorphous frozen solutions.

However, it is possible that the relaxation rates for these two samples are substantially overestimated due to the short measurement times compared with their observed spin dynamics. This would place large uncertainties on the extracted values of ω_α_, beyond what are reported above simply derived from the fitting algorithm.

## Conclusions

We have measured the zero-field temperature dependence and the 1.8 K field dependence of magnetic relaxation rates for polycrystalline and solution samples of [Dy(Cp^ttt^)_2_][B(C_6_F_5_)_4_]. We find that the temperature dependence of the “high-temperature” Raman-I regime is quite similar for all samples, experimentally confirming recent theoretical models. We also find similar results in the low-temperature QTM regime to previous measurements of QTM gaps on these same samples; however, we note that the most dilute sample (10 mM in DFB solution) is rather strangely behaved and is likely the result of many-body effects or aggregation that cannot be modeled. As a function of the magnetic field, the reduction in relaxation rates in most of the concentrated solution samples is well behaved and is limited by the standard field-independent Raman-I regime that can be modeled with a power-law temperature dependence. For the most concentrated DCM sample (200 mM) and the polycrystalline material, the Raman-I rates are much slower than the other samples at the lowest temperatures, which we ascribe to a phonon-pair-driven Raman-I process with Γ-point optical phonon modes on the order of ω_α_ = 18.8(3.3) K and ω_α_ = 31.2(3.6) K, respectively, suggesting that choice of solvent and dilution level has a nontrivial impact on the low-energy phonon spectrum.

## Data Availability

The magnetization decay traces of [Dy(Cp^ttt^)_2_][B(C_6_F_5_)_4_] can be obtained via FigShare at DOI: 10.48420/27116863.

## Supporting Information Available

Individual zero-field and infield decay plots with a description of how they were modeled and temperature- and field-dependent relaxation profiles for all samples along with any modeling (PDF)
