# Supplementary material for: In-Field and Zero-Field Relaxation Dynamics of Dysprosocenium in Solution
Source: J Phys Chem A. 2025 Feb 25;129(9):2144–50. doi: 10.1021/acs.jpca.4c06678 (PMC11891893; doi:10.1021/acs.jpca.4c06678)
Supplement: Supplementary file 1 — jp4c06678_si_001.pdf [file jp4c06678_si_001.pdf]

In-field and zero-field relaxation dynamics of dysprosocenium in solution

William J. A. Blackmore,[†] Sophie C. Corner,[†] Peter Evans,[†] Gemma K. Gransbury,[†] David P. Mills,[*,†] and Nicholas F. Chilton[*,‡,†]

†*Department of Chemistry, School of Natural Sciences, University of Manchester, Oxford Road, Manchester, M13 9PL, UK*

‡*Research School of Chemistry, The Australian National University, Building 137, Sullivans Creek Road, Canberra, ACT, 2601, Australia.*

E-mail: david.mills@manchester.ac.uk; nicholas.chilton@anu.edu.au

Find M_{eq} in zero-field

The negative equilibrium magnetisation in the higher temperature zero-field decay measurements is due to a lack of calibration of the applied field using a standard Pd sample. Indeed, these measurements were what inspired the in depth study of DC decay measurements using a Quantum Design MPMS3 described in Blackmore *et al.*[S1]. From this study, we believe the true field is in the region $-20 \leq \mu_0 H \leq 25$ Oe. Therefore, M_{eq} is expected to follow Curie-like behaviour. Due to the large amounts of solvent in these samples, there is also a possibility of a significant diamagnetic response. All zero-field decays were fitted to Eq. 1 with M_0 fixed to the first measured point and M_{eq} free to fit. At higher temperatures where the sample has almost completely decayed, the M_{eq} data can be modelled (Fig. S1 - S6) using

$$M_{eq} = \frac{C}{T} + D, \tag{S1}$$

where C is the Curie constant and D is the temperature independent diamagnetic contribution. This relationship was extrapolated to lower temperatures, allowing us to fix M_{eq} for all zero-field decays.

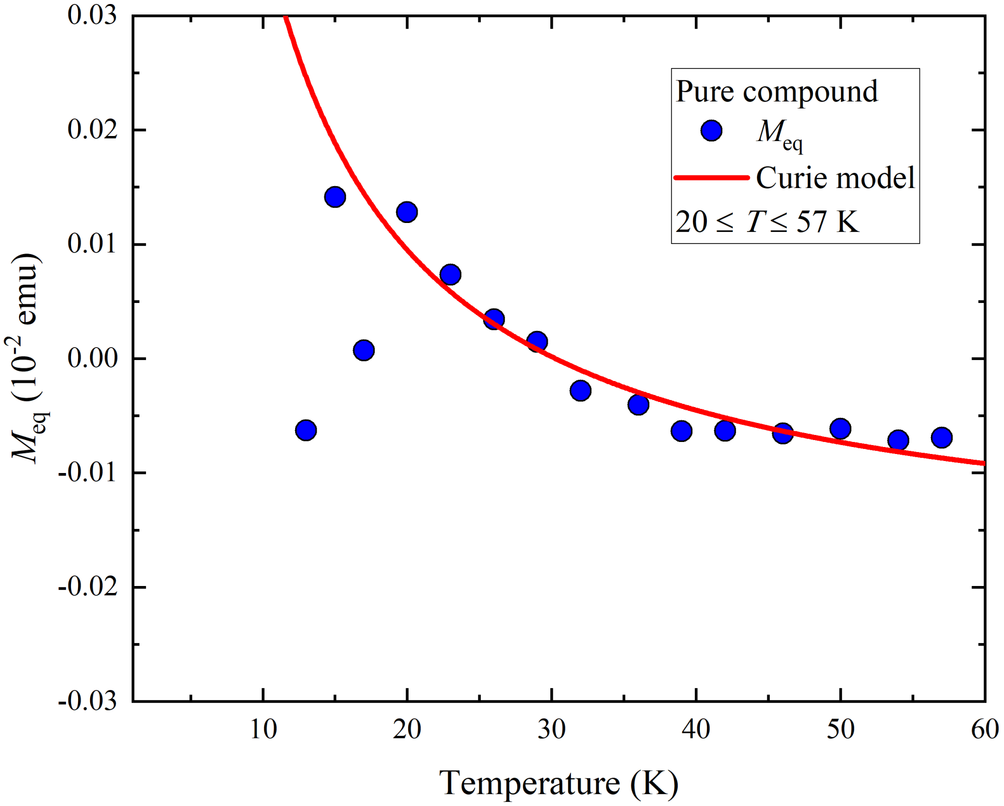

Figure S1: Fit of the high temperature M_{eq} data of the polycrystalline sample to Eq. S1

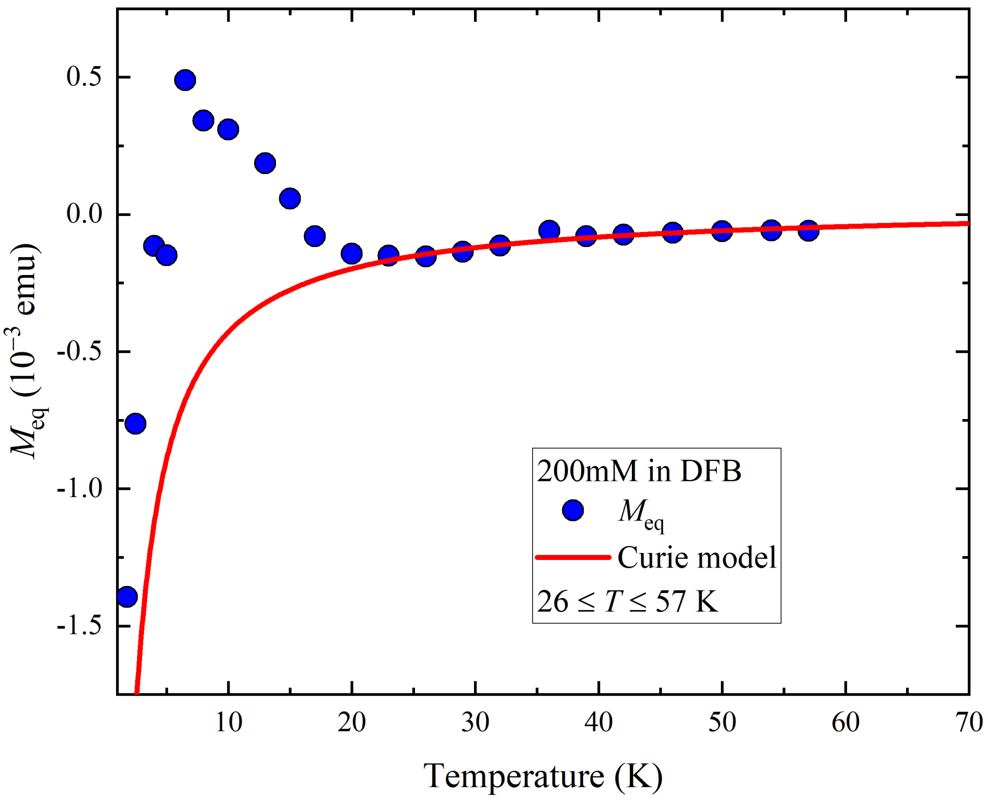

Figure S2: Fit of the high temperature M_{eq} data of the 200mM in DFB sample to Eq. S1

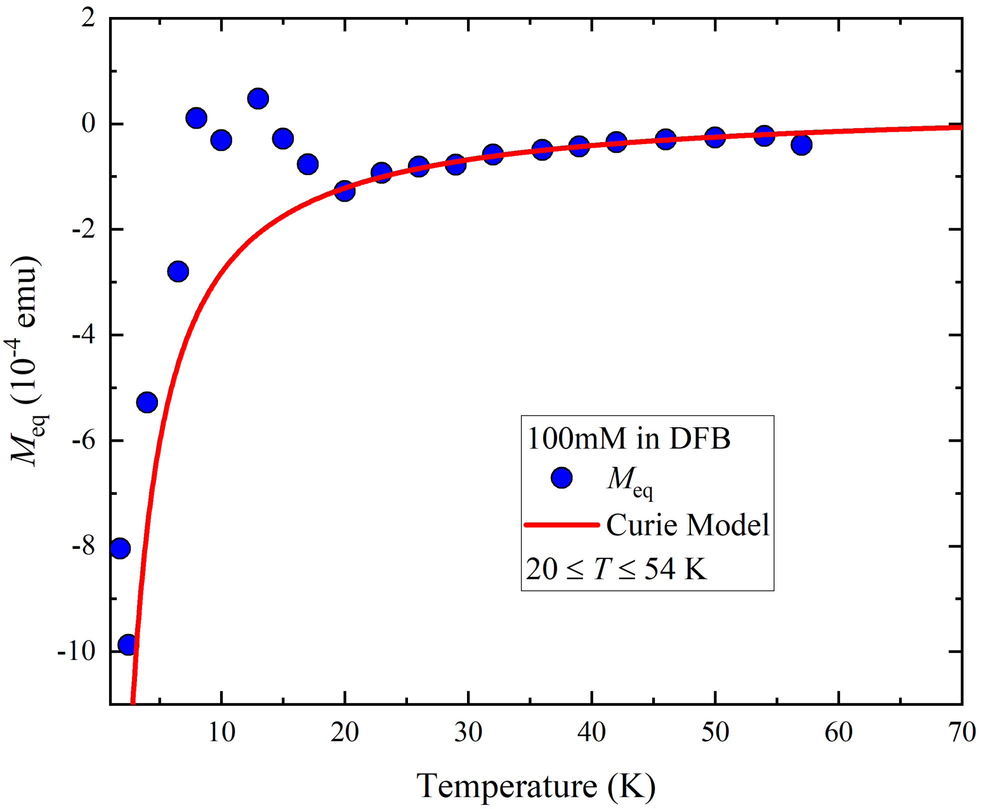

Figure S3: Fit of the high temperature M_{eq} data of the 100mM in DFB sample to to Eq. S1

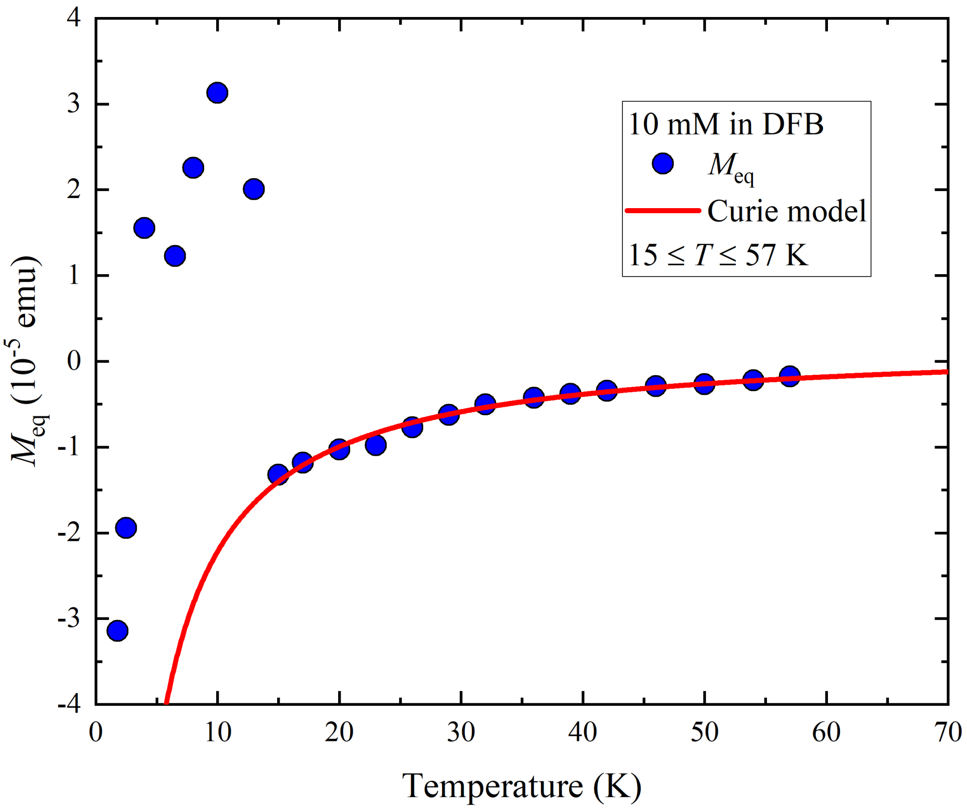

Figure S4: Fit of the high temperature M_{eq} data of the 10mM in DFB sample to to Eq. S1

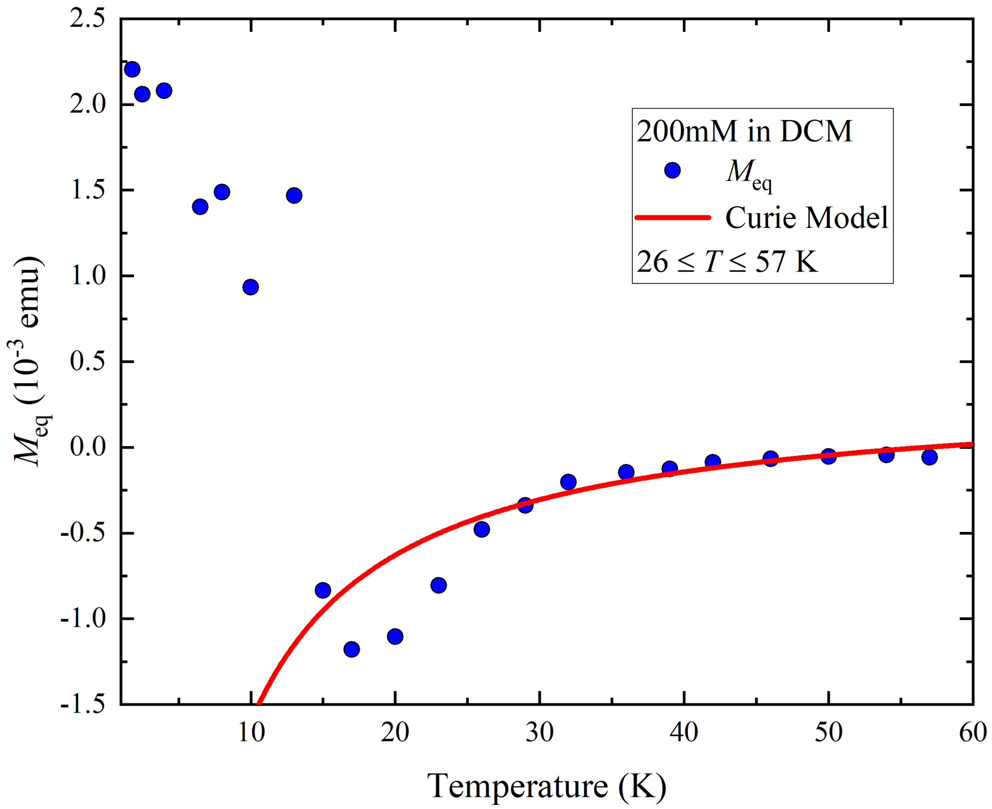

Figure S5: Fit of the high temperature M_{eq} data of the 200mM in DCM sample to to Eq. S1

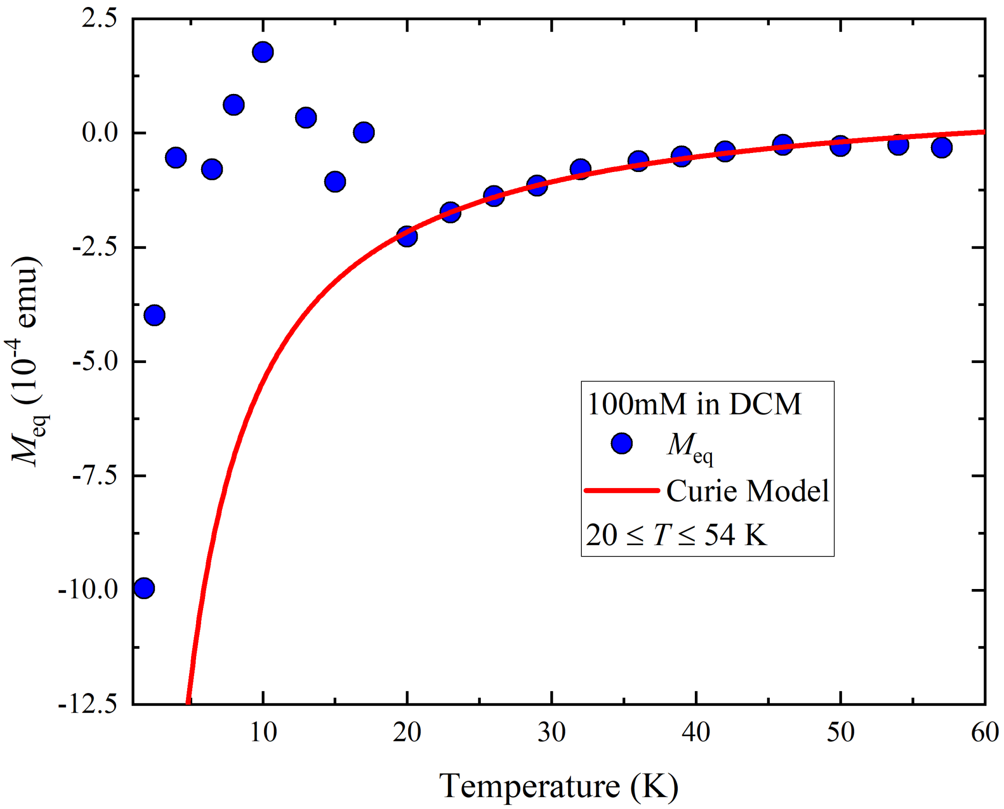

Figure S6: Fit of the high temperature M_{eq} data of the 100mM in DCM sample to to Eq. S1

Zero-field decays

Zero-field DC decay measurements of $[Dy(Cp^{ttt})_2][B(C_6F_5)_4]$ were modelled using Eq. 1, with M_0 fixed to the first data point at measured zero-field, and M_{eq} fixed to a value calculated using Eq. S1 (see previous section). The data where $M < 0.01 \times M_0$ was not fitted.[S1]

Polycrystalline

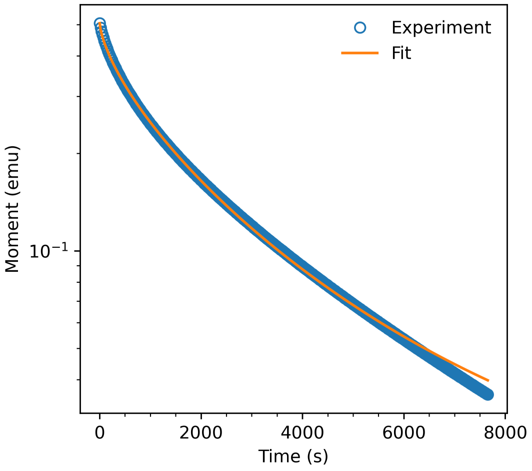

(a) 1.8 K

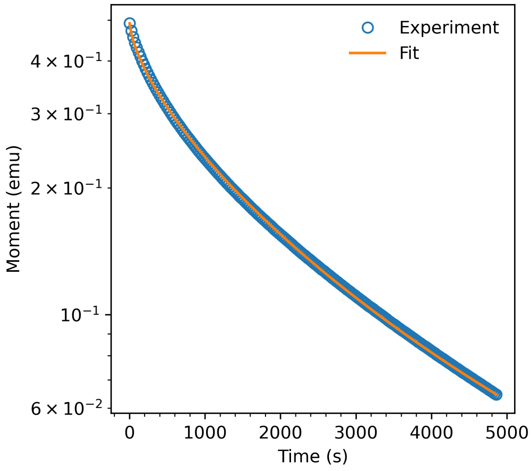

(b) 2.5 K

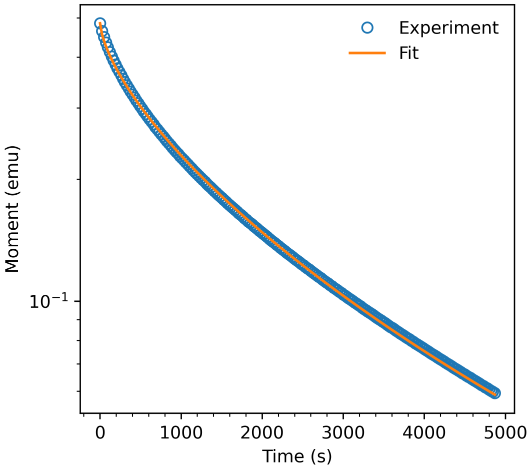

(c) 4 K

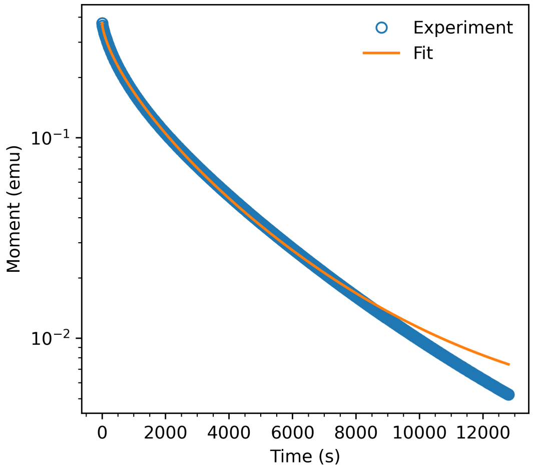

(d) 6.5 K

Figure S7: Zero-field DC Decay measurements of polycrystalline $[Dy(Cp^{ttt})_2][B(C_6F_5)_4]$.

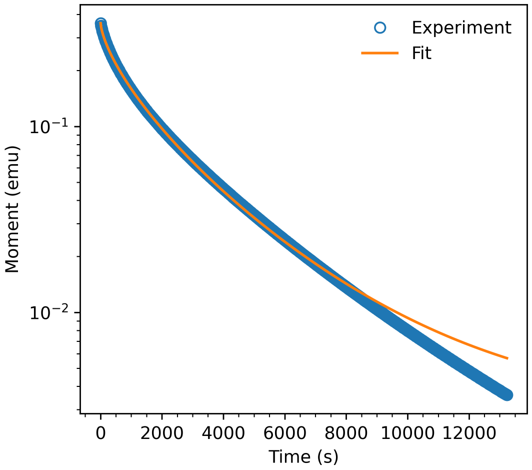

(a) 8 K

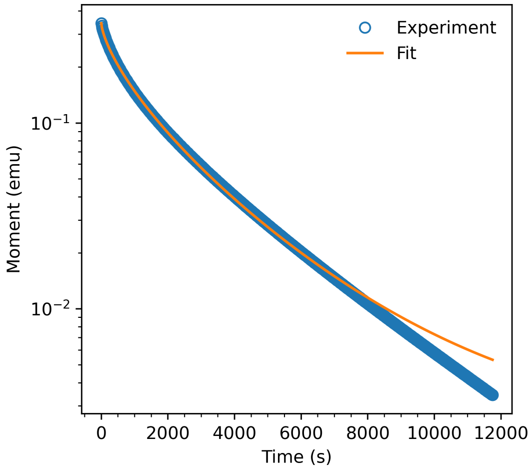

(b) 10 K

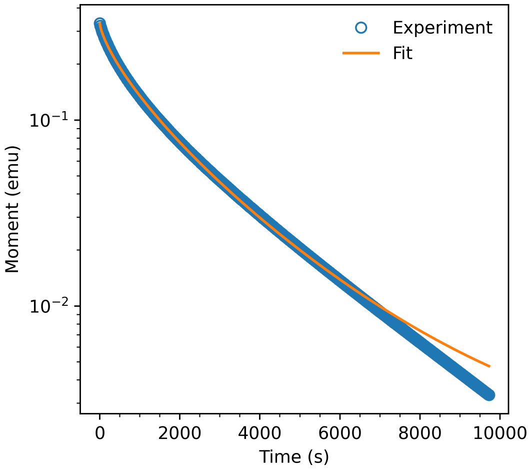

(c) 13 K

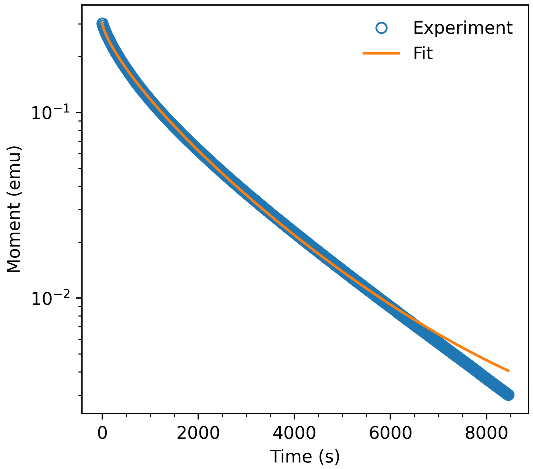

(d) 15 K

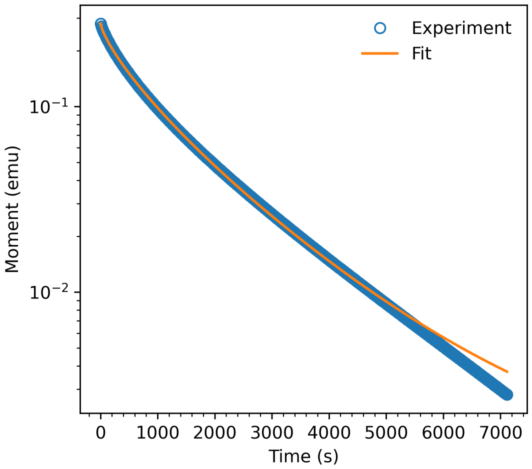

(e) 17 K

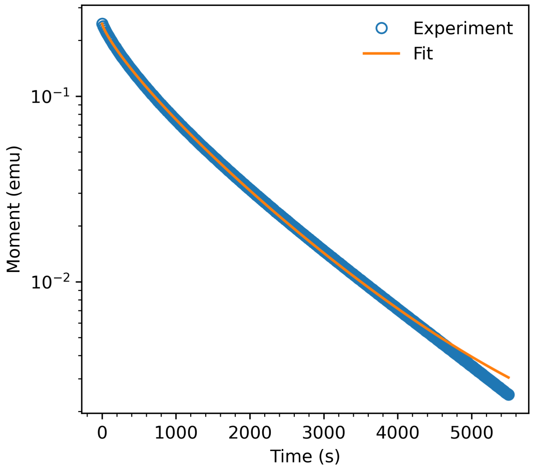

(f) 20 K

Figure S8: Zero-field DC Decay measurements of polycrystalline $[Dy(Cp^{ttt})_2][B(C_6F_5)_4]$.

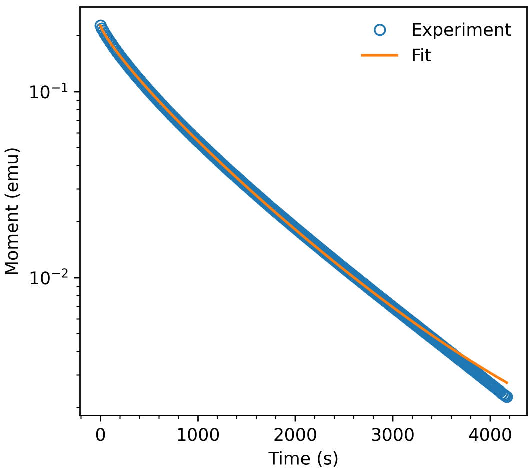

(a) 23 K

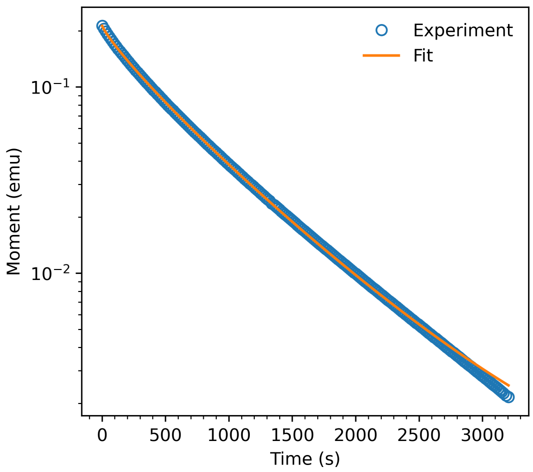

(b) 26 K

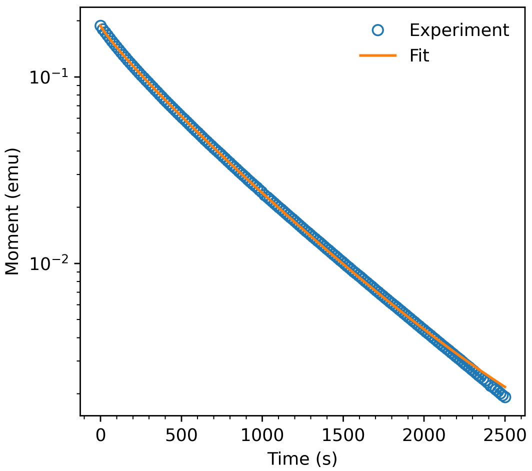

(c) 29 K

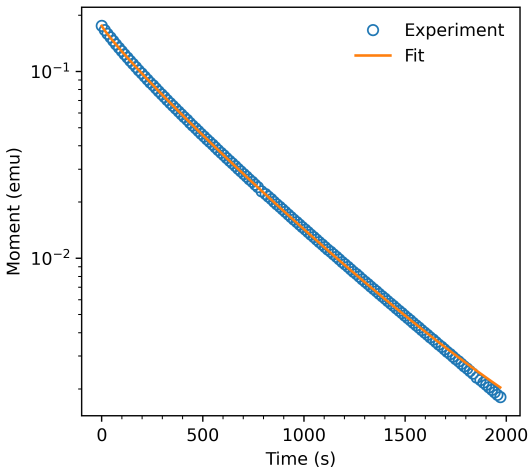

(d) 32 K

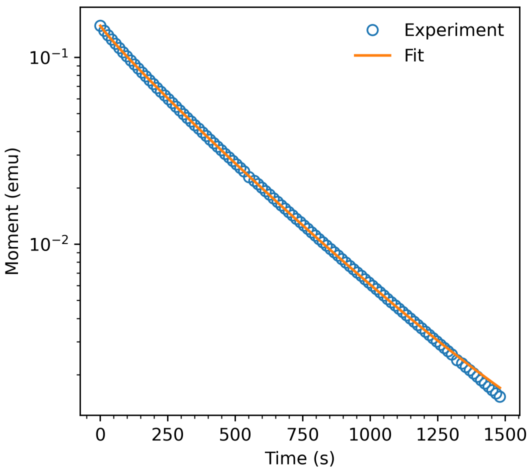

(e) 36 K

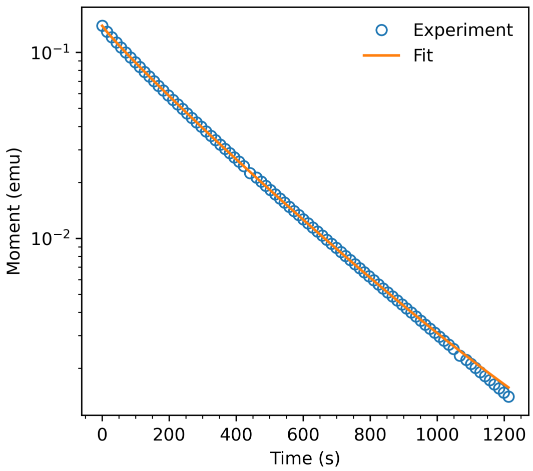

(f) 39 K

Figure S9: Zero-field DC Decay measurements of polycrystalline $[Dy(Cp^{ttt})_2][B(C_6F_5)_4]$.

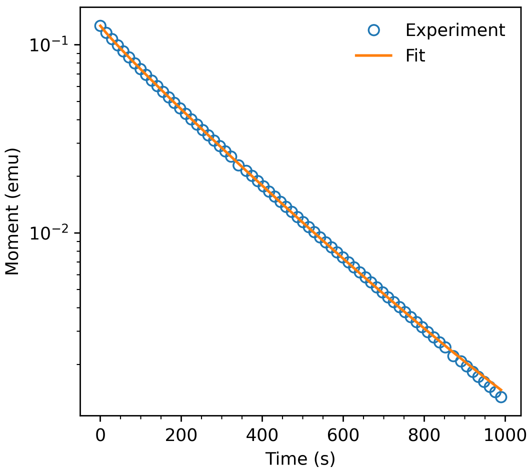

(a) 42 K

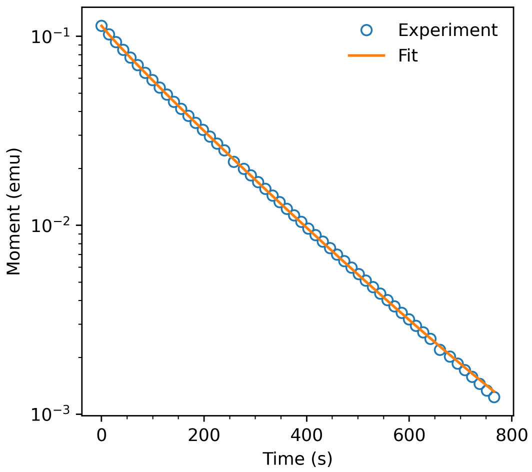

(b) 46 K

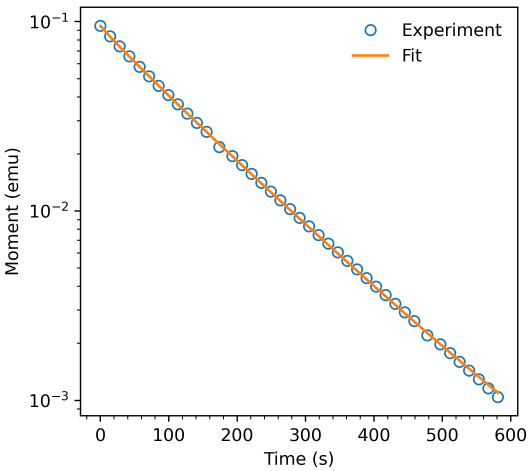

(c) 50 K

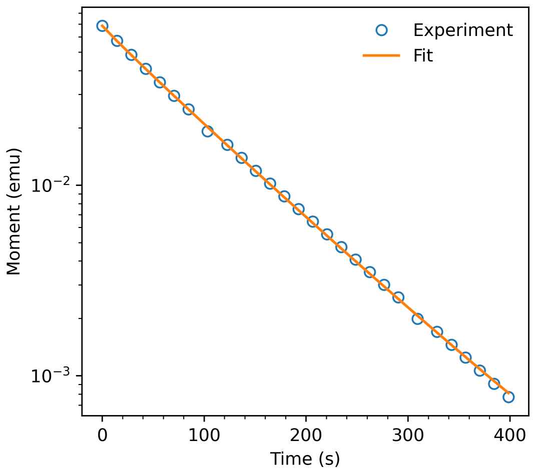

(d) 54 K

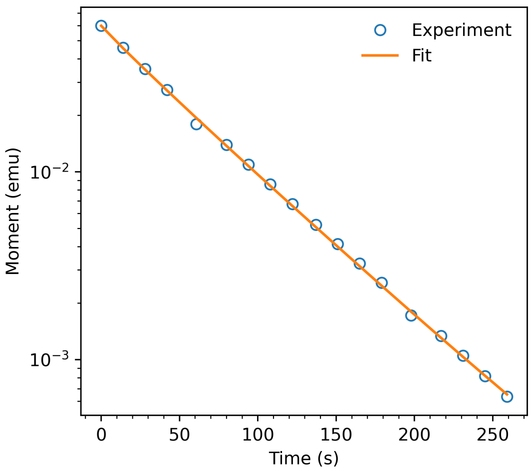

(e) 57 K

Figure S10: Zero-field DC Decay measurements of polycrystalline $[Dy(Cp^{ttt})_2][B(C_6F_5)_4]$.

DFB

200mM

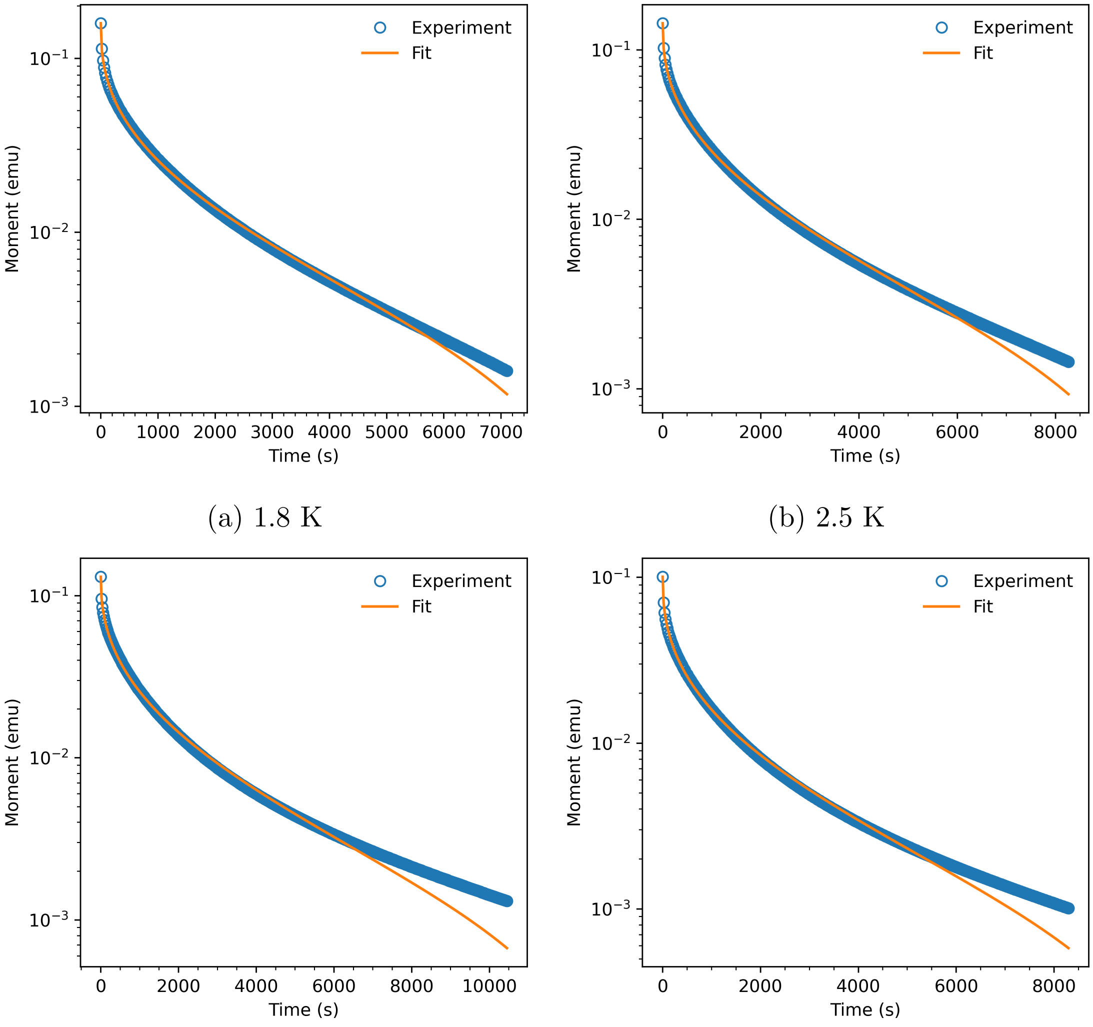

(a) 1.8 K (b) 2.5 K

(c) 4 K (d) 5 K

Figure S11: Zero-field DC Decay measurements for 200mM of $[Dy(Cp^{ttt})_2][B(C_6F_5)_4]$ dissolved in DFB

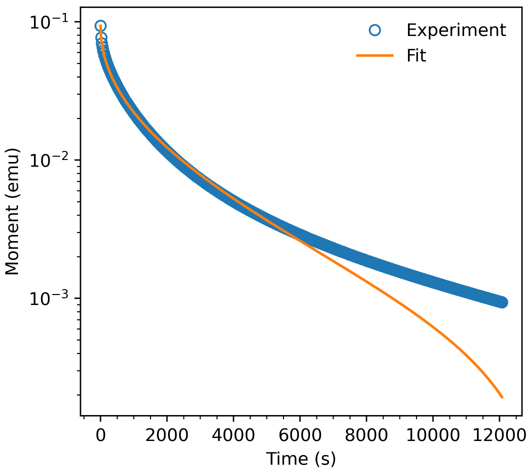

(a) 6.5 K

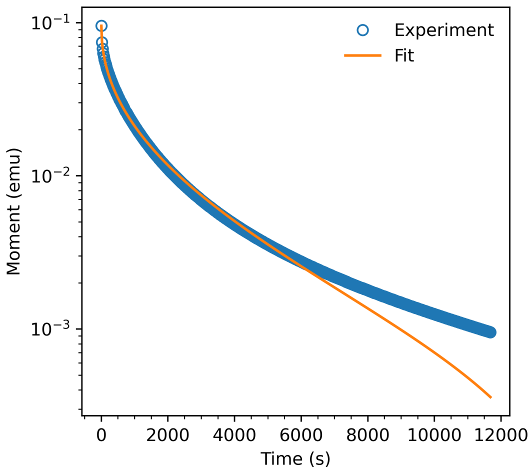

(b) 8 K

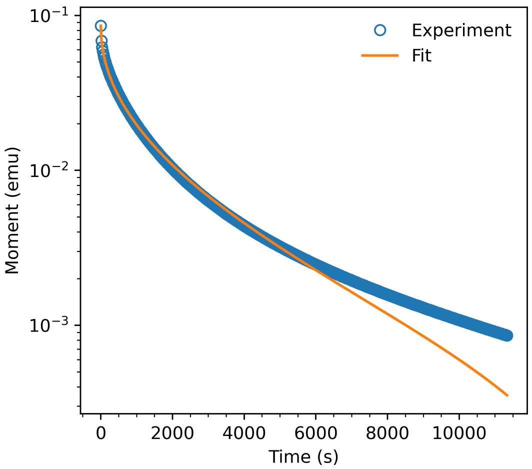

(c) 10 K

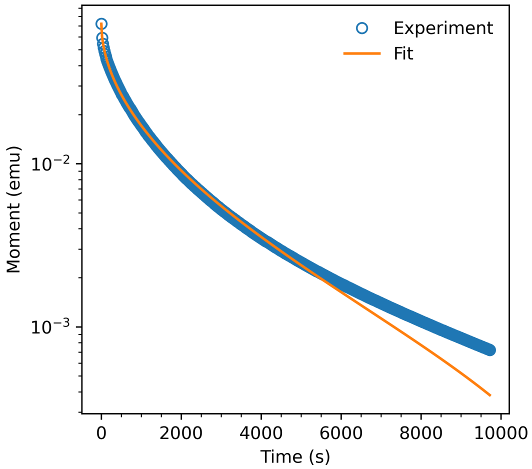

(d) 13 K

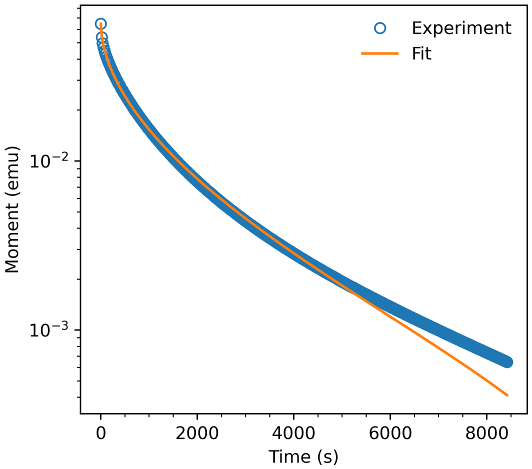

(e) 15 K

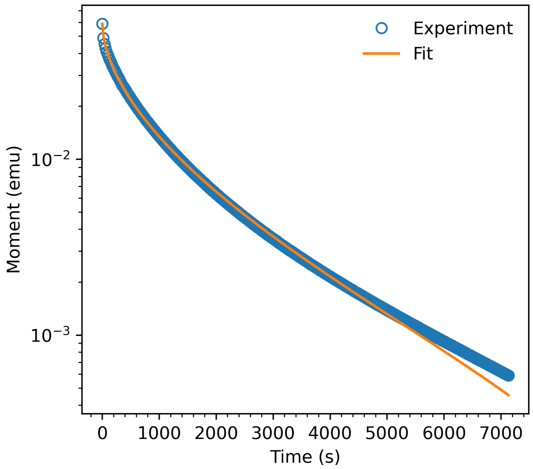

(f) 17 K

Figure S12: Zero-field DC Decay measurements for 200mM of $[Dy(Cp^{ttt})_2][B(C_6F_5)_4]$ dissolved in DFB

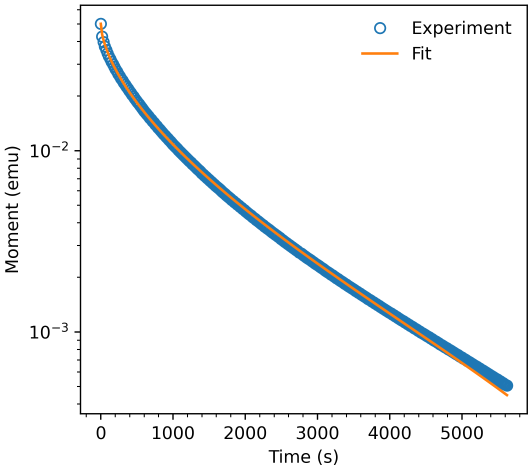

(a) 20 K

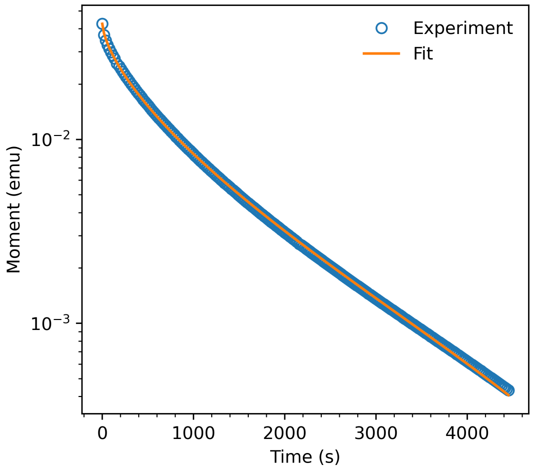

(b) 23 K

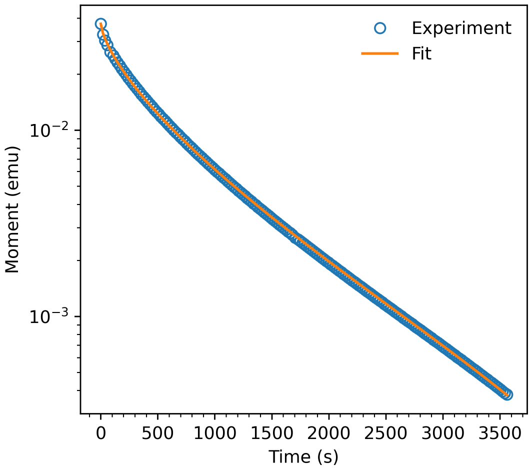

(c) 26 K

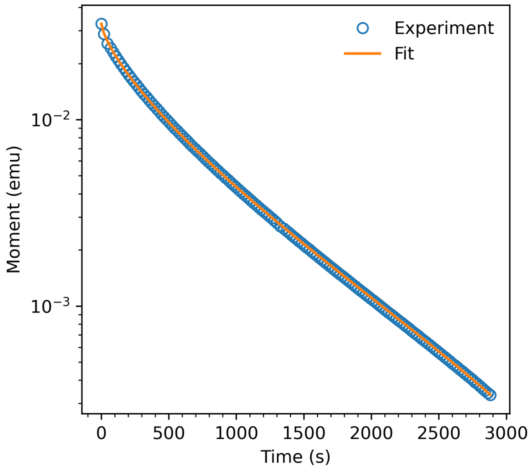

(d) 29 K

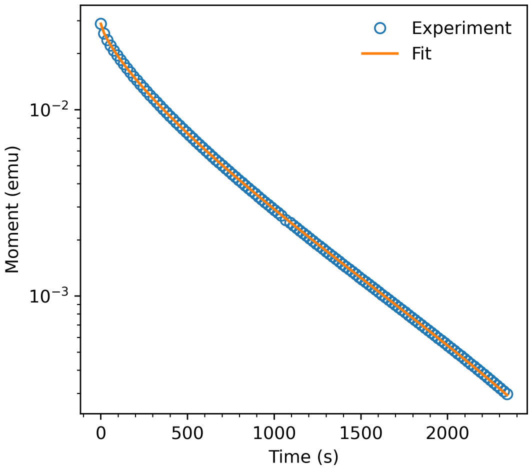

(e) 32 K

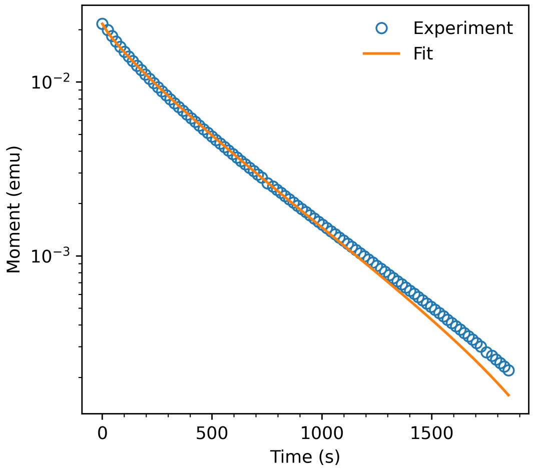

(f) 36 K

Figure S13: Zero-field DC Decay measurements for 200mM of $[Dy(Cp^{ttt})_2][B(C_6F_5)_4]$ dissolved in DFB

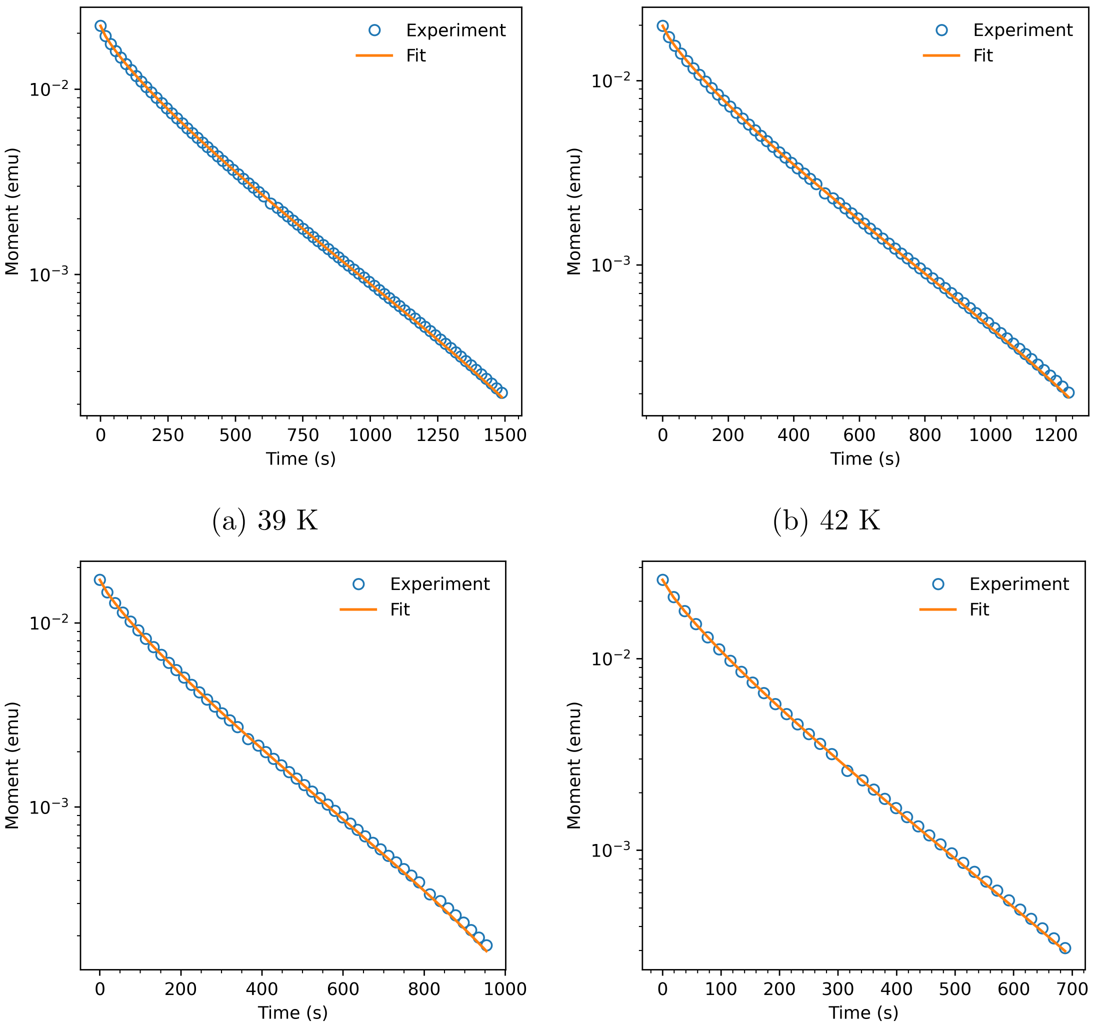

(a) 39 K (b) 42 K

(c) 46 K (d) 50 K

Figure S14: Zero-field DC Decay measurements for 200mM of $[Dy(Cp^{ttt})_2][B(C_6F_5)_4]$ dissolved in DFB

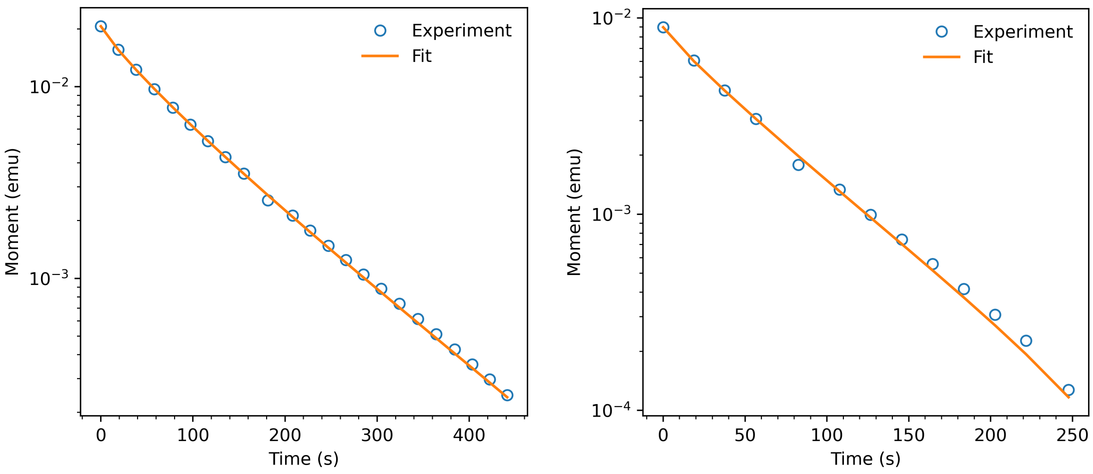

(a) 54 K

(b) 57 K

Figure S15: Zero-field DC Decay measurements for 200mM of $[Dy(Cp^{ttt})_2][B(C_6F_5)_4]$ dissolved in DFB

100mM

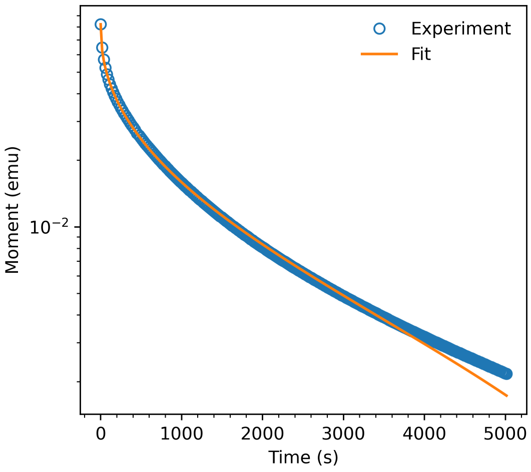

(a) 1.8 K

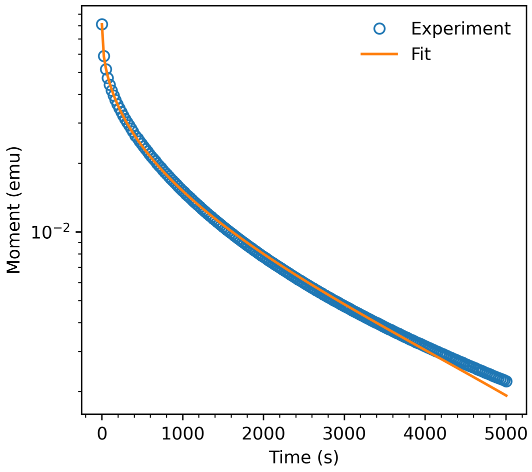

(b) 2.5 K

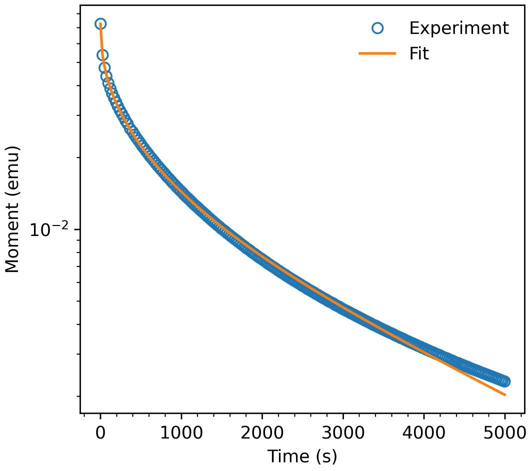

(c) 4 K

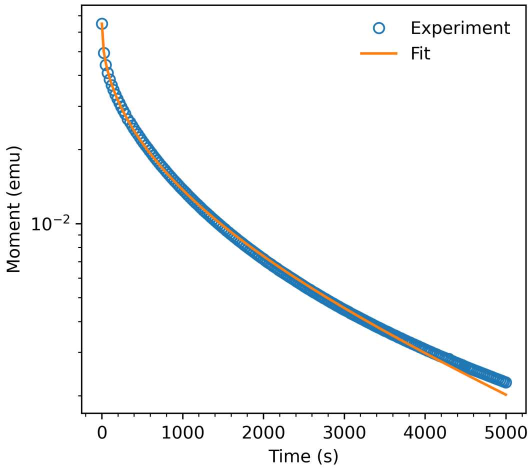

(d) 6.5 K

Figure S16: Zero-field DC Decay measurements for 100mM of $[Dy(Cp^{ttt})_2][B(C_6F_5)_4]$ dissolved in DFB

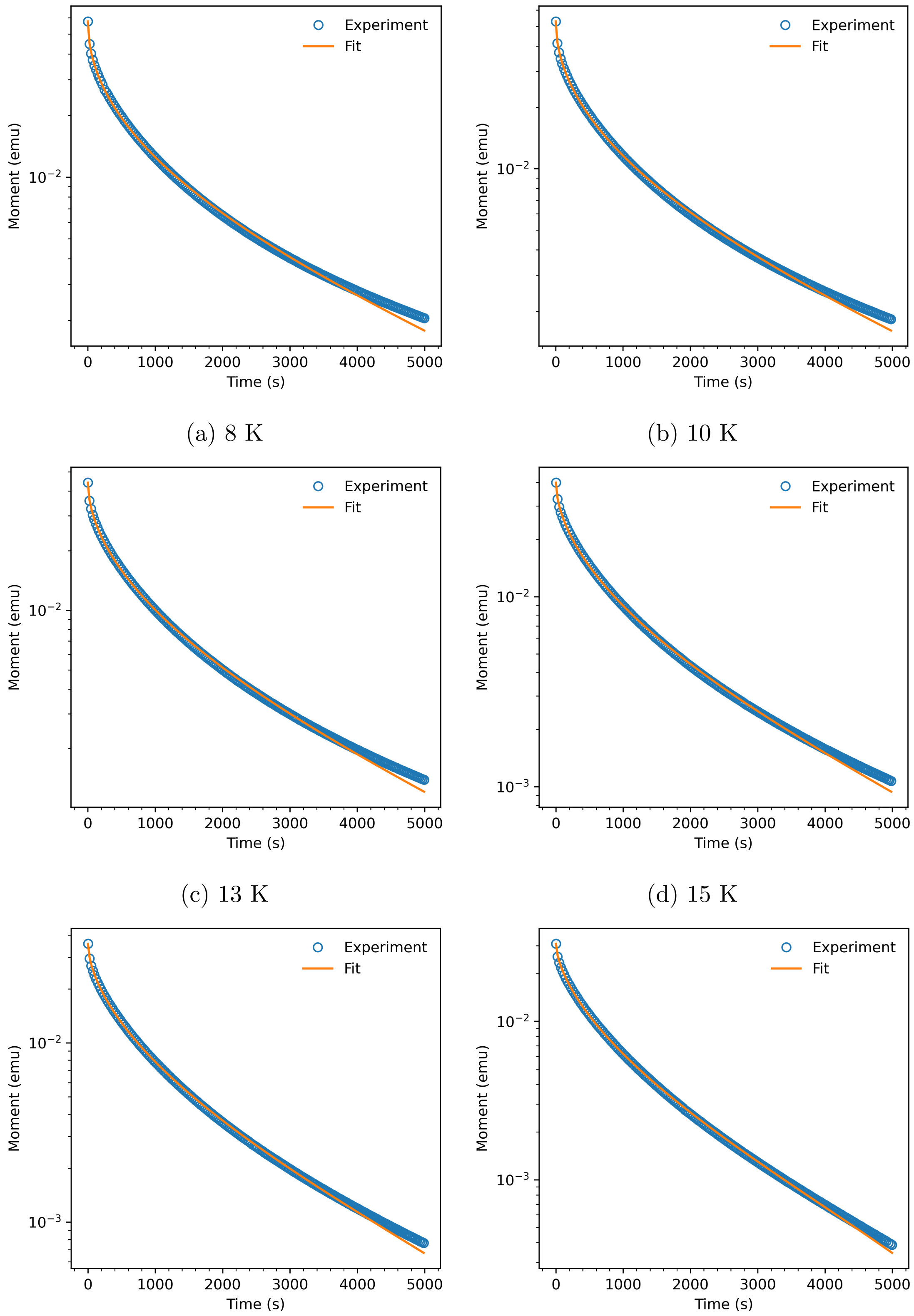

(a) 8 K (b) 10 K

(c) 13 K (d) 15 K

(e) 17 K (f) 20 K

Figure S17: Zero-field DC Decay measurements for 100mM of $[Dy(Cp^{ttt})_2][B(C_6F_5)_4]$ dissolved in DFB

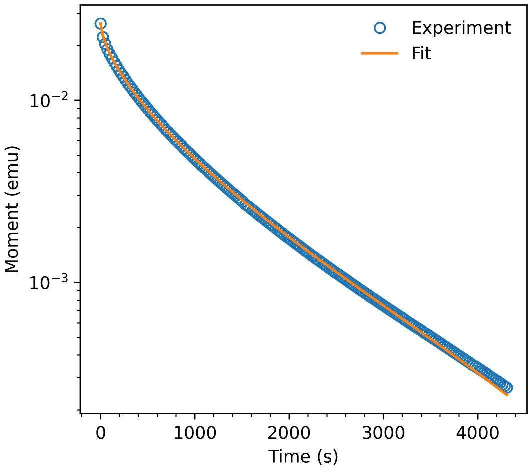

(a) 23 K

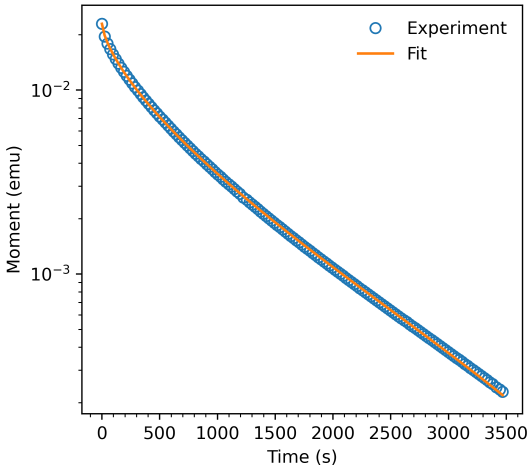

(b) 26 K

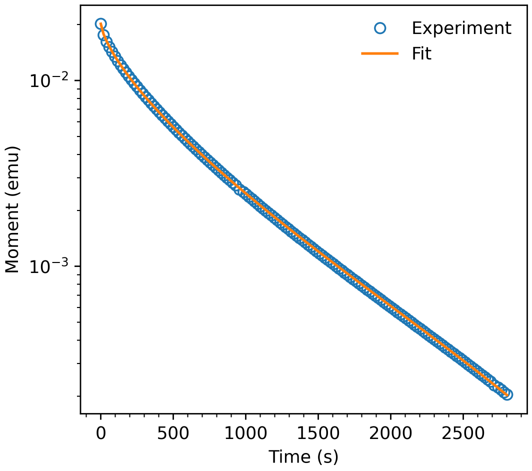

(c) 29 K

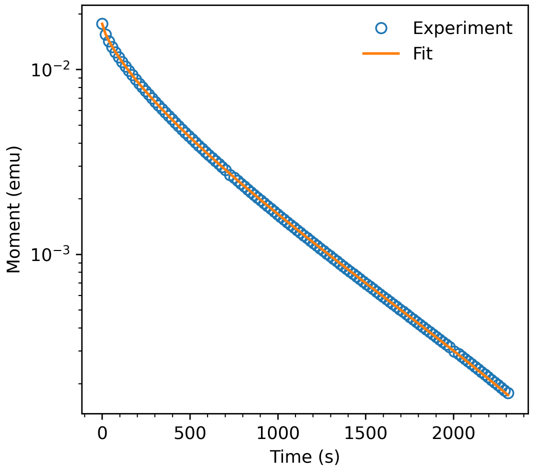

(d) 32 K

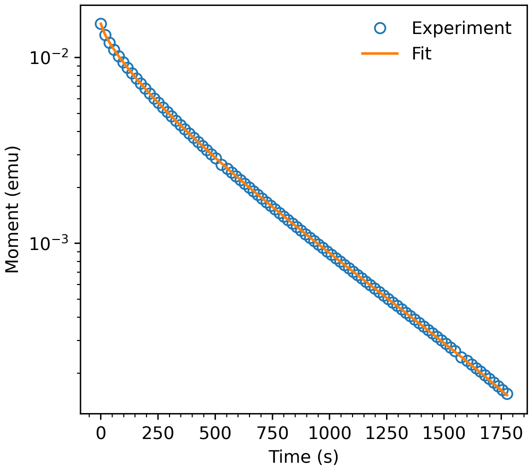

(e) 36 K

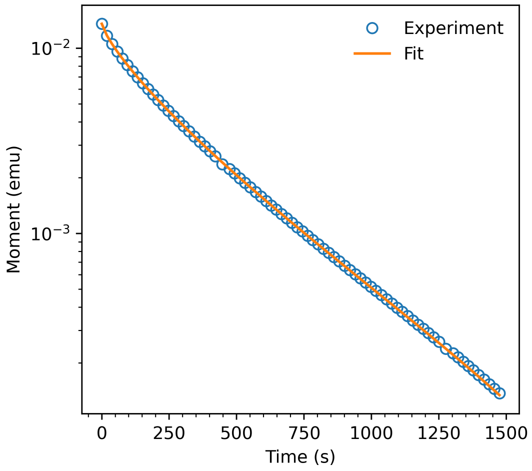

(f) 39 K

Figure S18: Zero-field DC Decay measurements for 100mM of $[Dy(Cp^{ttt})_2][B(C_6F_5)_4]$ dissolved in DFB

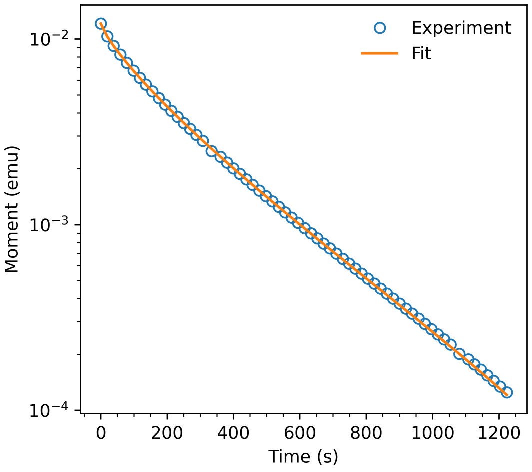

(a) 42 K

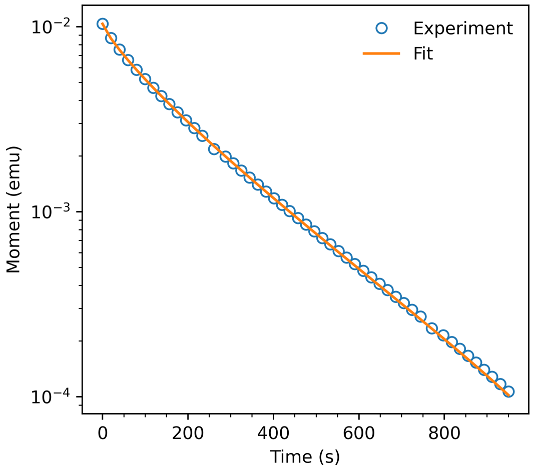

(b) 46 K

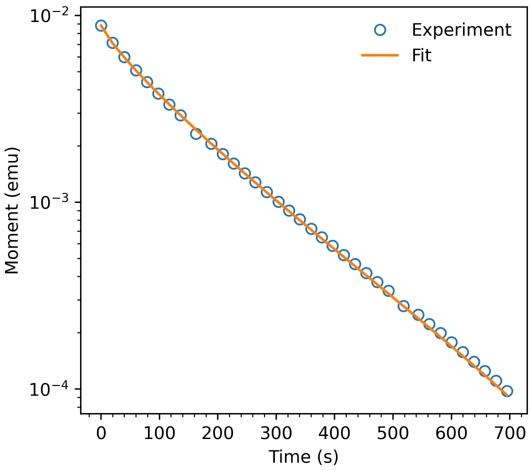

(c) 50 K

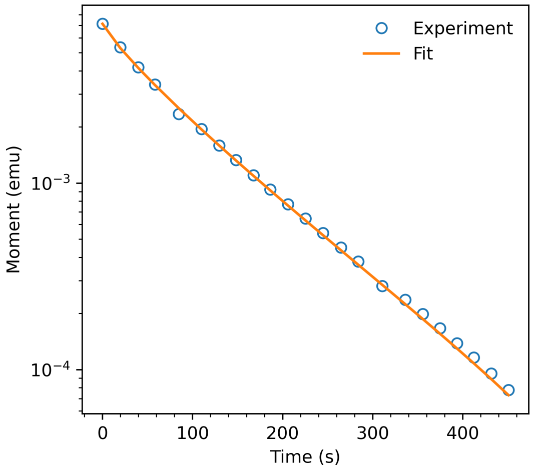

(d) 54 K

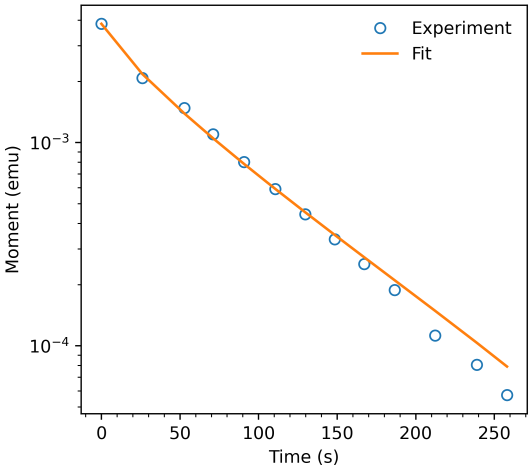

(e) 57 K

Figure S19: Zero-field DC Decay measurements for 100mM of $[Dy(Cp^{ttt})_2][B(C_6F_5)_4]$ dissolved in DFB

10mM

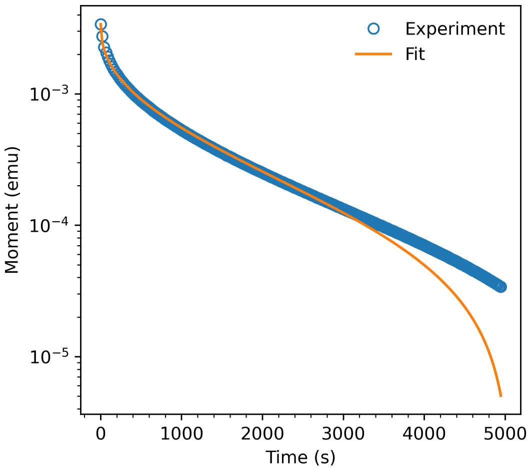

(a) 1.8 K

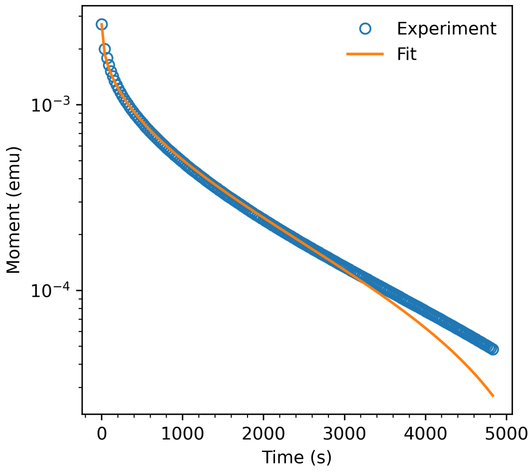

(b) 2.5 K

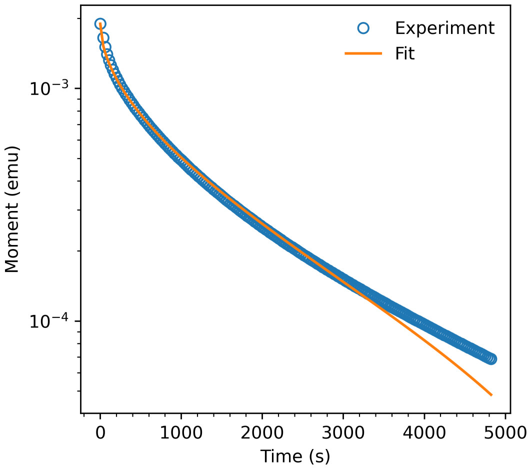

(c) 4 K

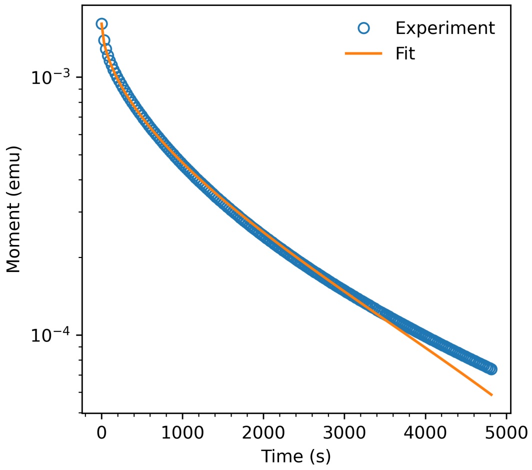

(d) 6.5 K

Figure S20: Zero-field DC Decay measurements for 10mM of $[Dy(Cp^{ttt})_2][B(C_6F_5)_4]$ dissolved in DFB

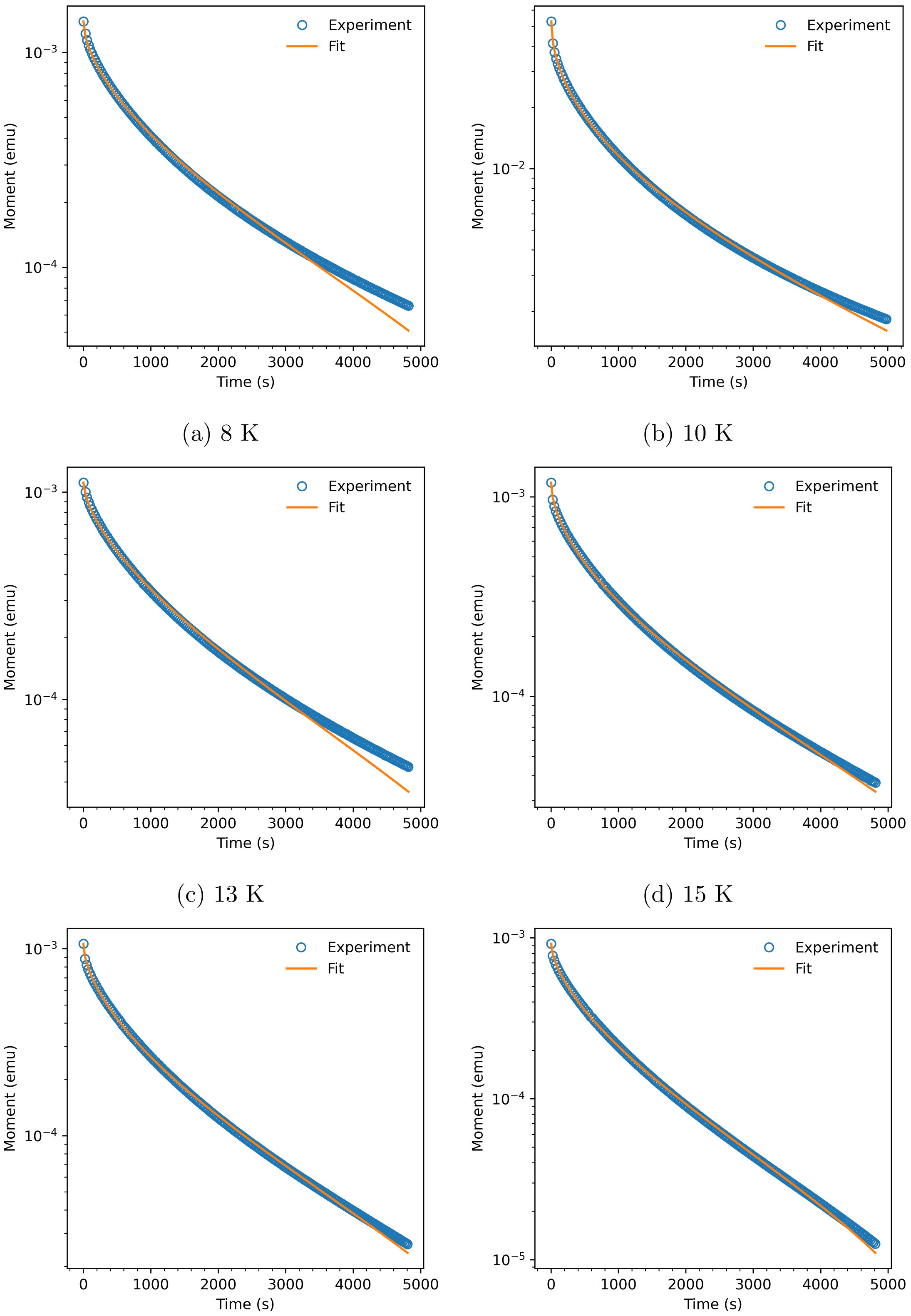

(a) 8 K (b) 10 K

(c) 13 K (d) 15 K

(e) 17 K (f) 20 K

Figure S21: Zero-field DC Decay measurements for 10mM of $[Dy(Cp^{ttt})_2][B(C_6F_5)_4]$ dissolved in DFB

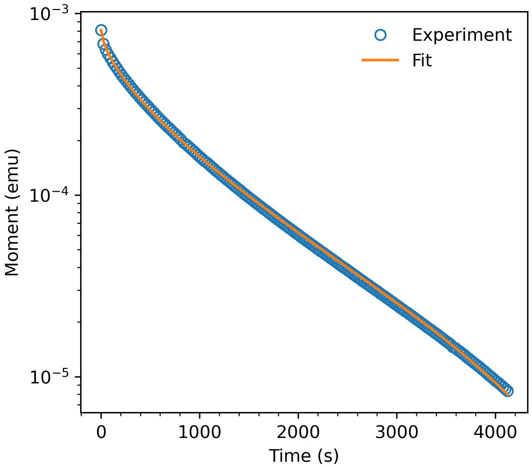

(a) 23 K

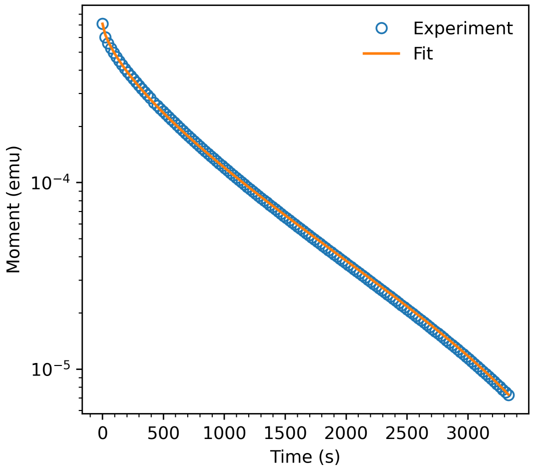

(b) 26 K

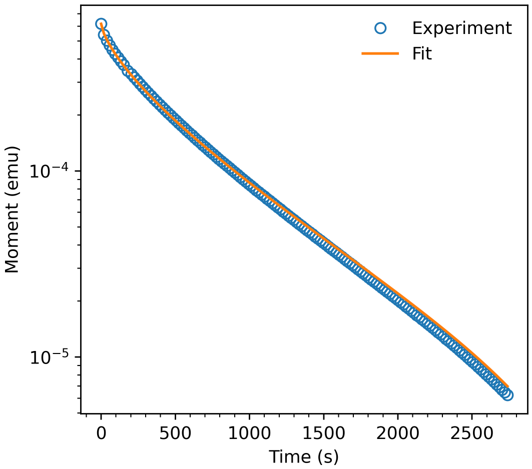

(c) 29 K

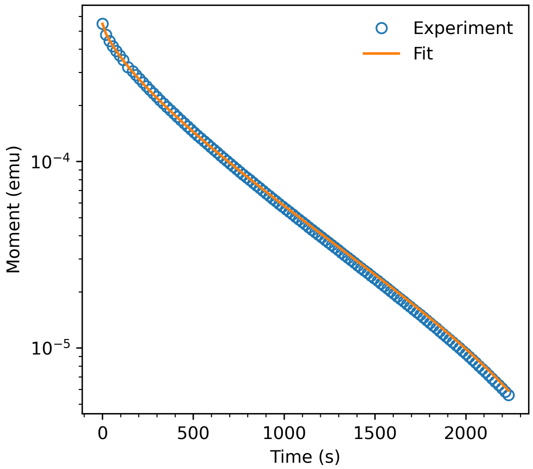

(d) 32 K

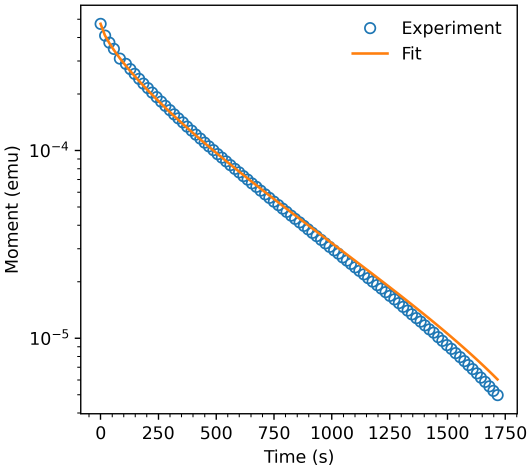

(e) 36 K

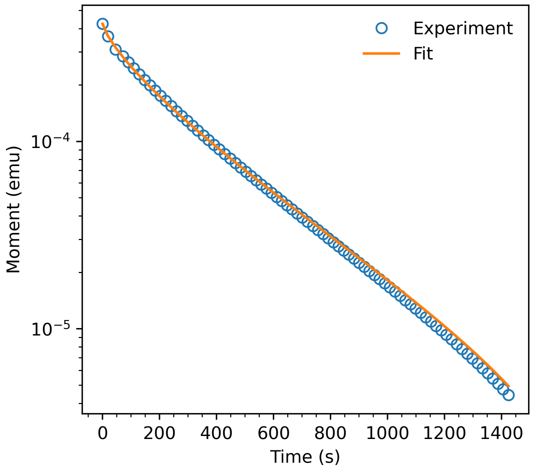

(f) 39 K

Figure S22: Zero-field DC Decay measurements for 10mM of $[Dy(Cp^{ttt})_2][B(C_6F_5)_4]$ dissolved in DFB

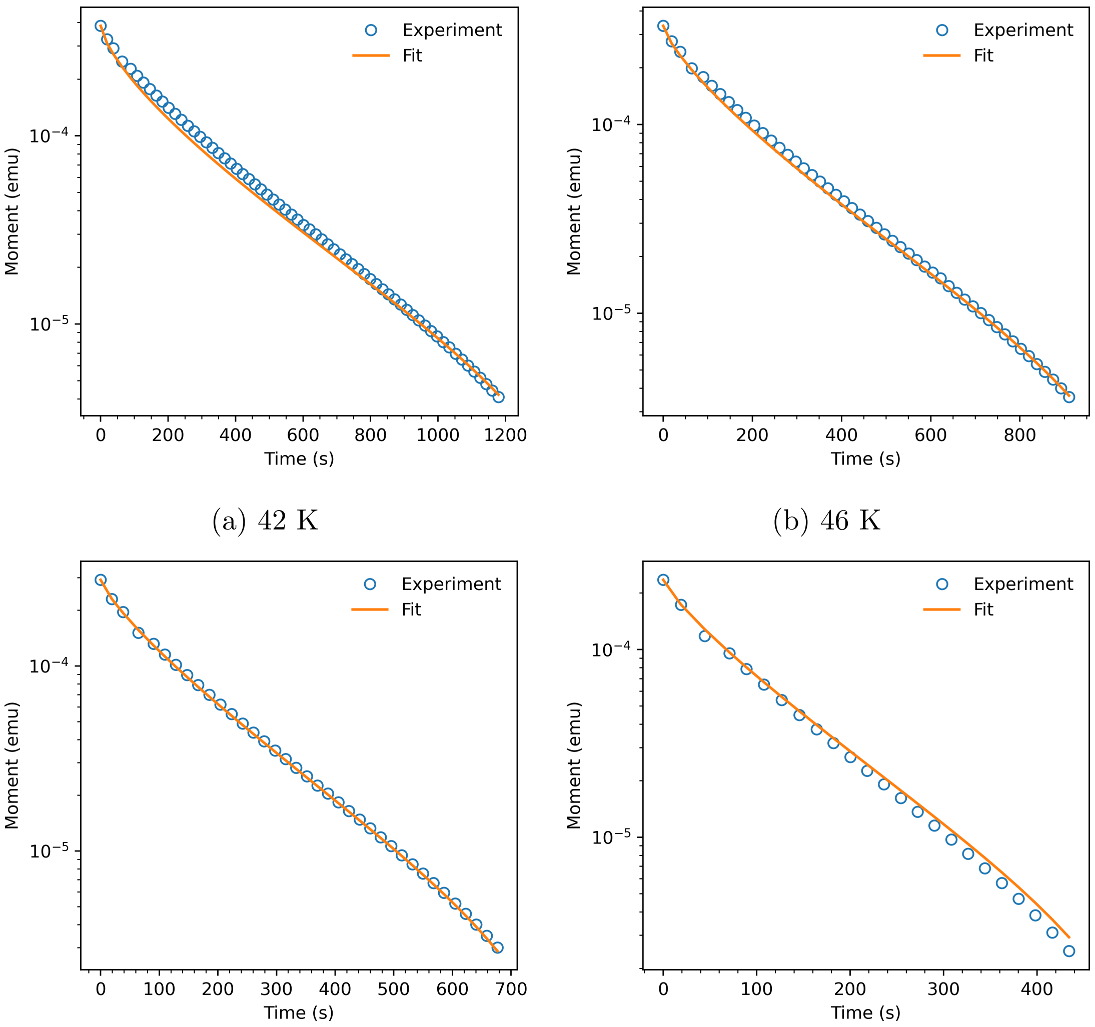

(a) 42 K

(b) 46 K

(c) 50 K

(d) 54 K

Figure S23: Zero-field DC Decay measurements for 10mM of $[Dy(Cp^{ttt})_2][B(C_6F_5)_4]$ dissolved in DFB

DCM

200mM

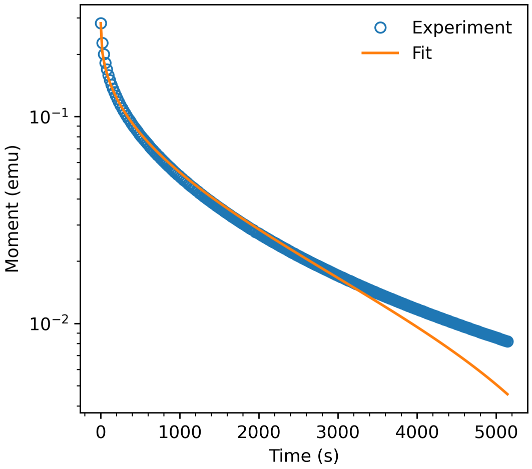

(a) 1.8 K

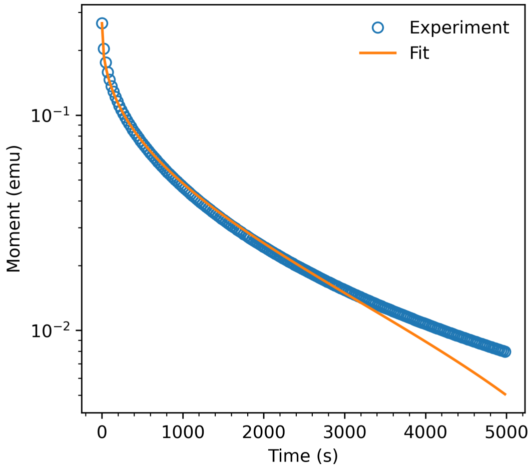

(b) 2.5 K

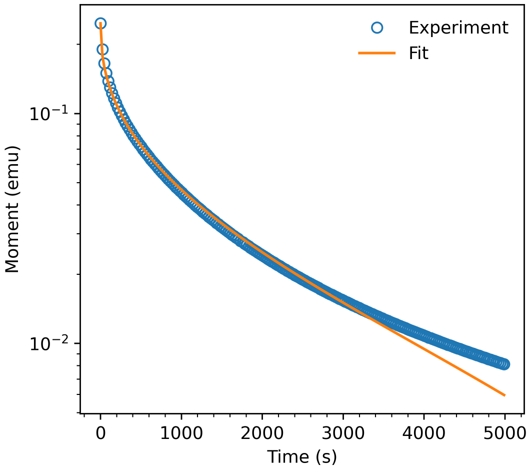

(c) 4 K

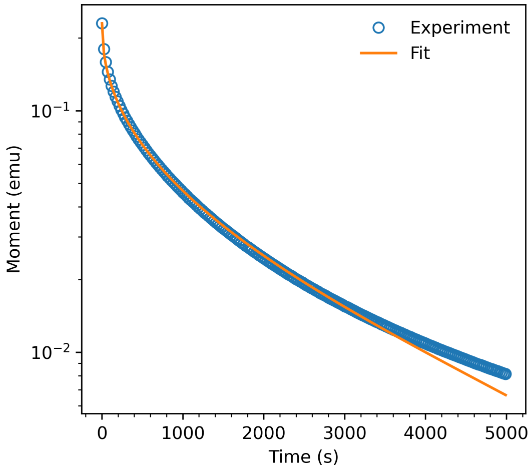

(d) 6.5 K

Figure S24: Zero-field DC Decay measurements for 200mM of $[Dy(Cp^{ttt})_2][B(C_6F_5)_4]$ dissolved in DCM

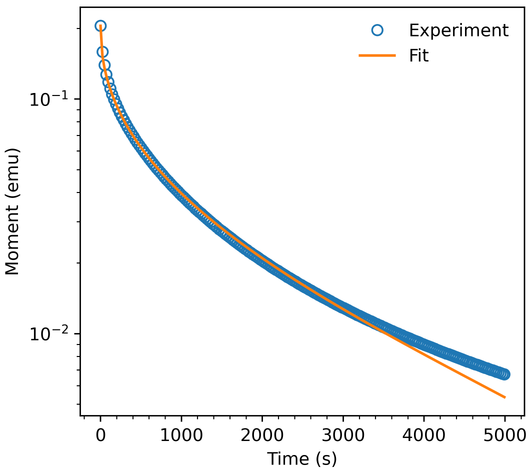

(a) 8 K

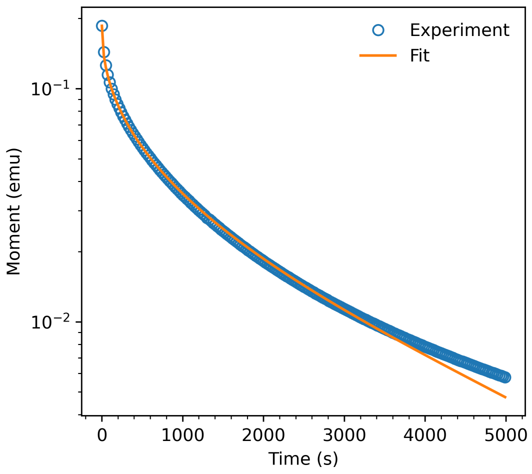

(b) 10 K

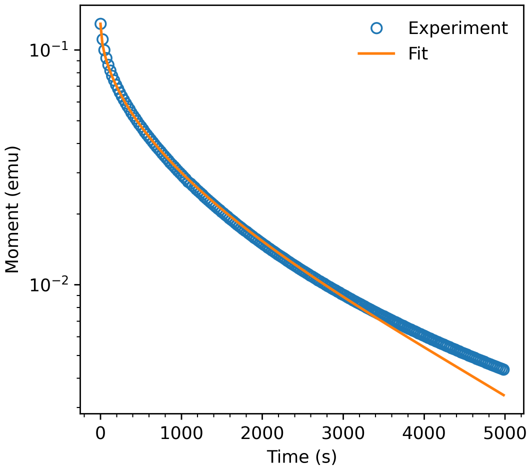

(c) 13 K

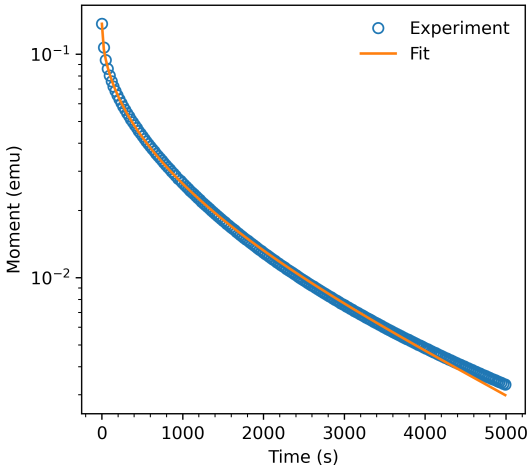

(d) 15 K

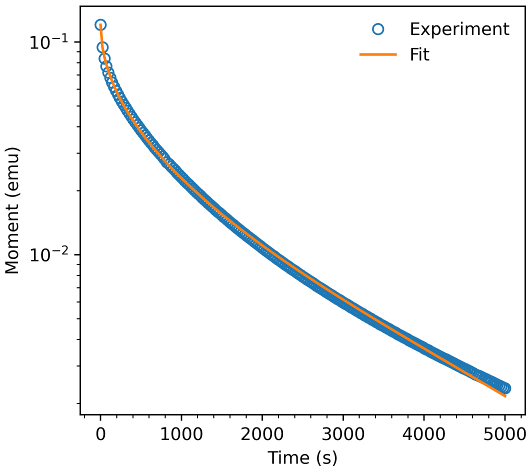

(e) 17 K

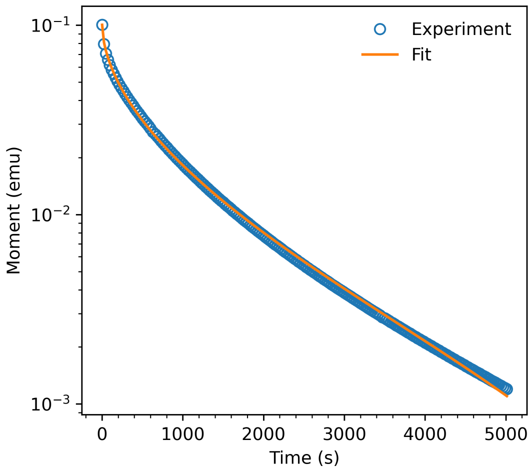

(f) 20 K

Figure S25: Zero-field DC Decay measurements for 200mM of $[Dy(Cp^{ttt})_2][B(C_6F_5)_4]$ dissolved in DCM

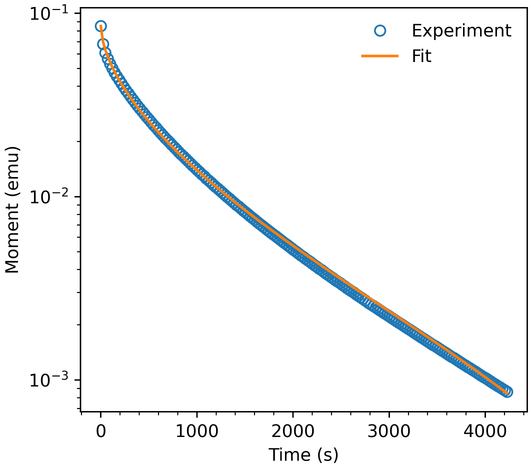

(a) 23 K

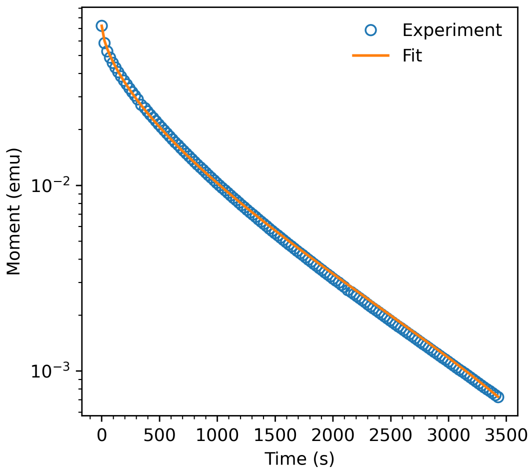

(b) 26 K

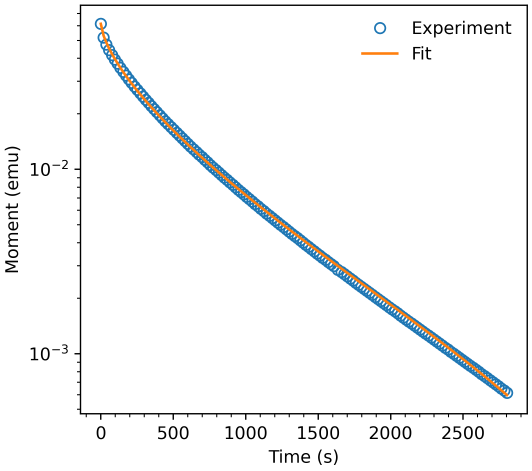

(c) 29 K

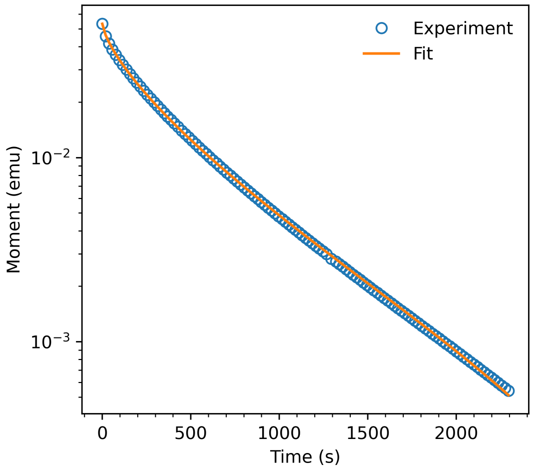

(d) 32 K

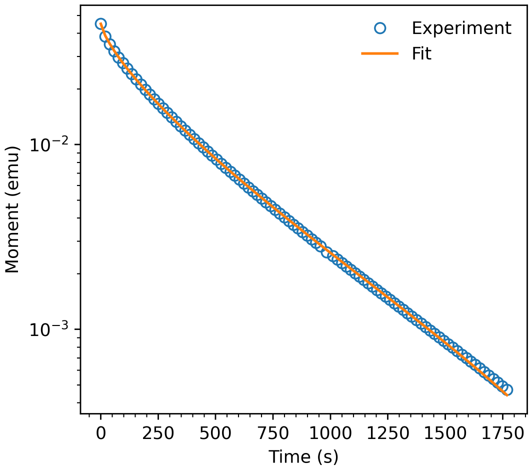

(e) 36 K

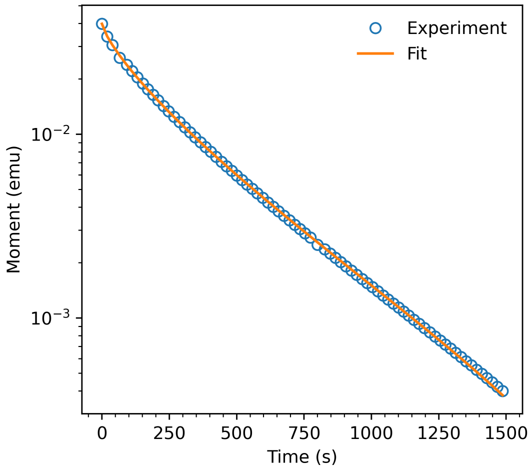

(f) 39 K

Figure S26: Zero-field DC Decay measurements for 200mM of $[Dy(Cp^{ttt})_2][B(C_6F_5)_4]$ dissolved in DCM

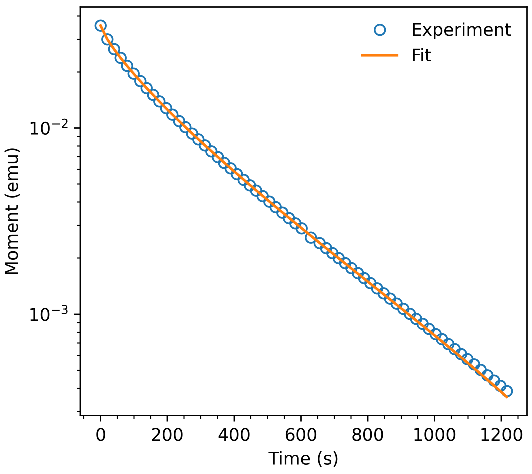

(a) 42 K

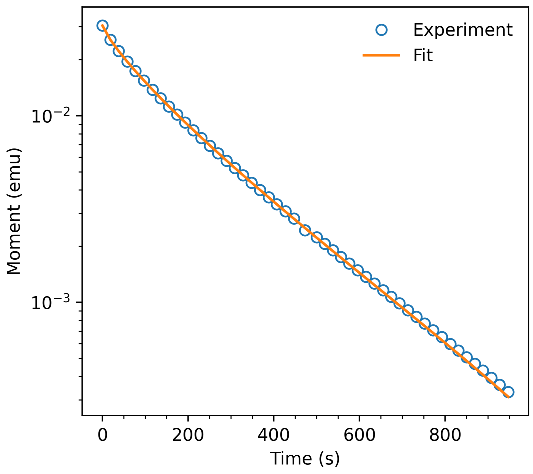

(b) 46 K

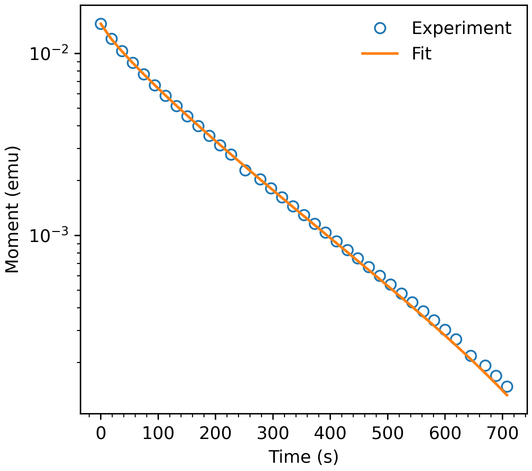

(c) 50 K

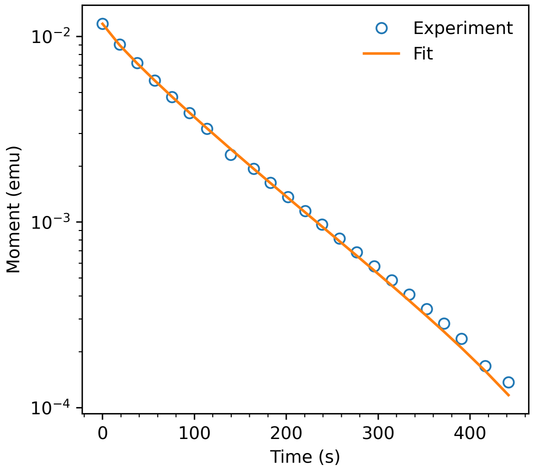

(d) 54 K

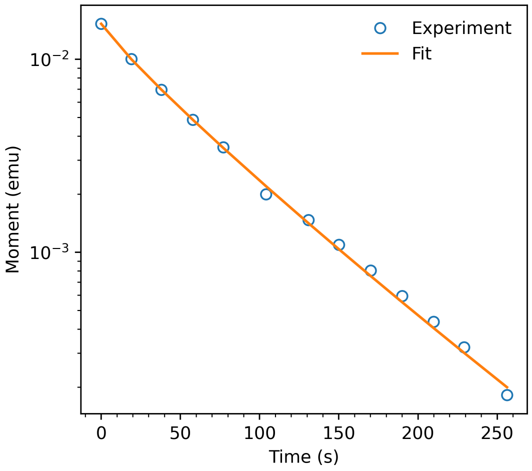

(e) 57 K

Figure S27: Zero-field DC Decay measurements for 200mM of $[Dy(Cp^{ttt})_2][B(C_6F_5)_4]$ dissolved in DCM

100mM

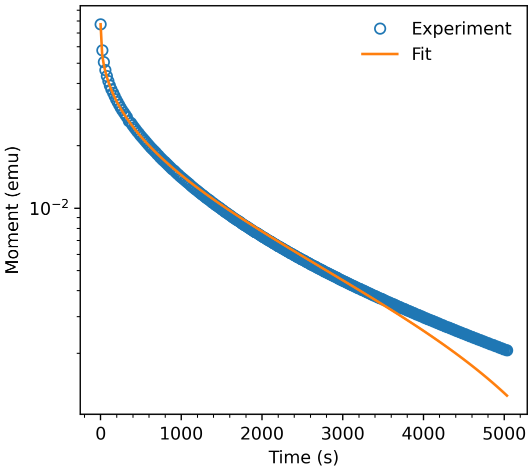

(a) 1.8 K

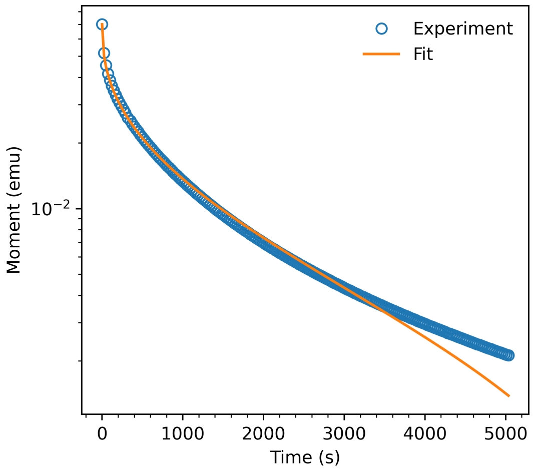

(b) 2.5 K

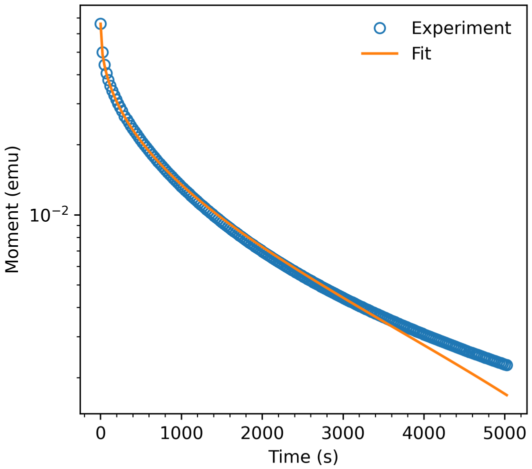

(c) 4 K

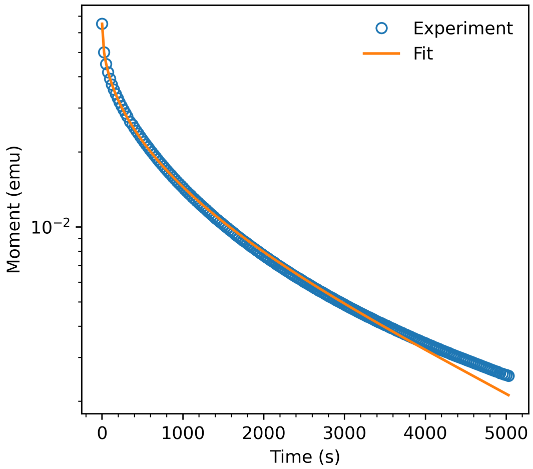

(d) 6.5 K

Figure S28: Zero-field DC Decay measurements for 100mM of $[Dy(Cp^{ttt})_2][B(C_6F_5)_4]$ dissolved in DCM

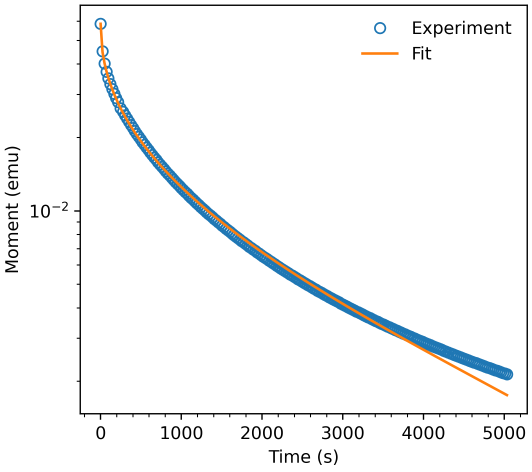

(a) 8 K

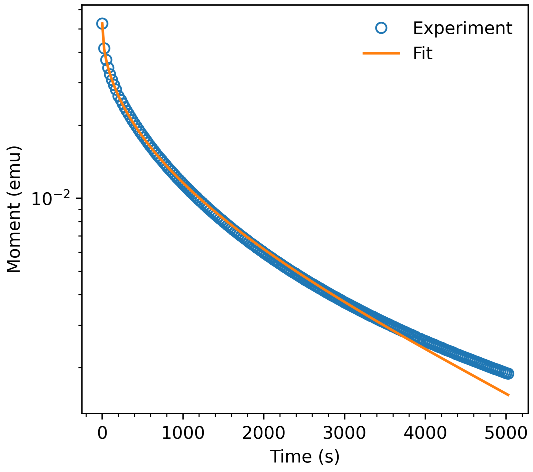

(b) 10 K

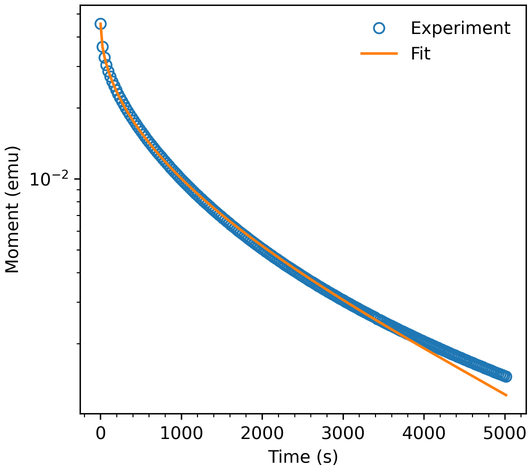

(c) 13 K

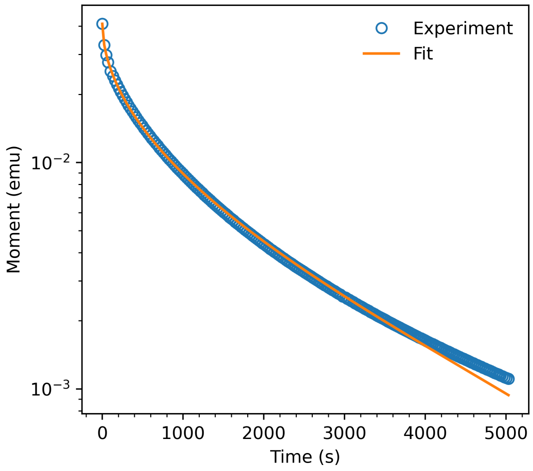

(d) 15 K

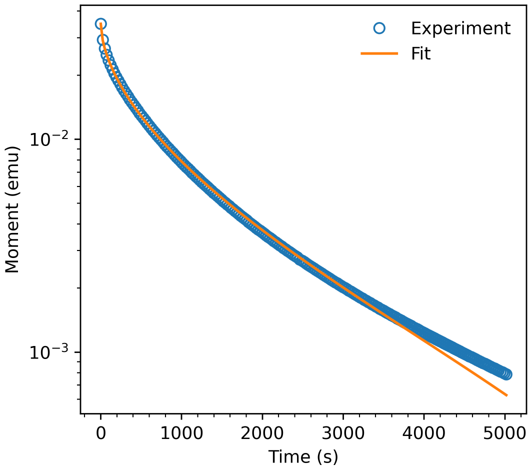

(e) 17 K

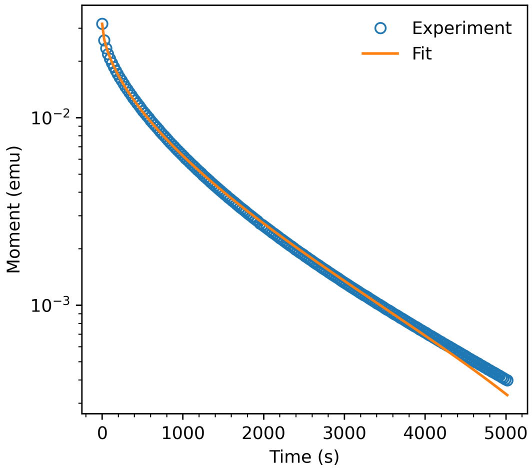

(f) 20 K

Figure S29: Zero-field DC Decay measurements for 100mM of $[Dy(Cp^{ttt})_2][B(C_6F_5)_4]$ dissolved in DCM

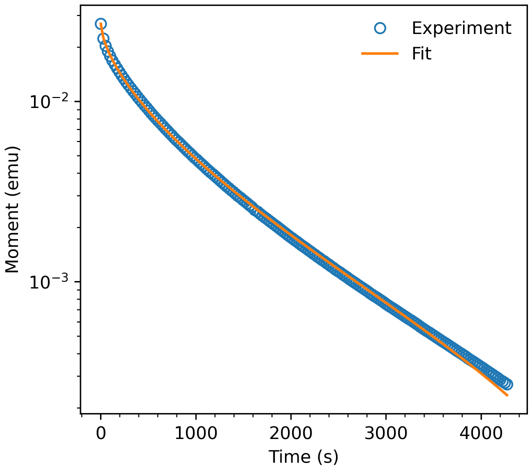

(a) 23 K

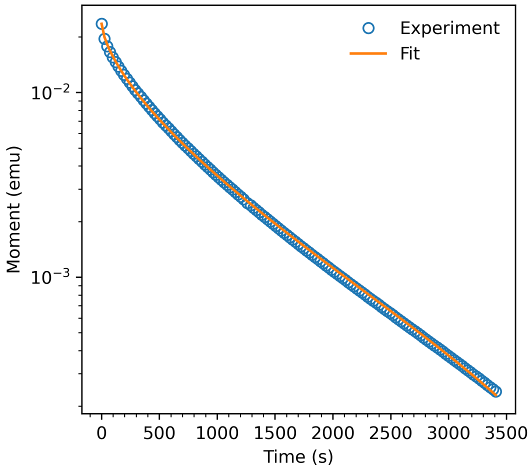

(b) 26 K

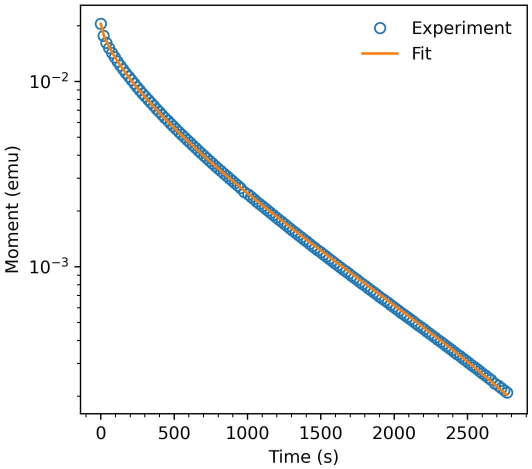

(c) 29 K

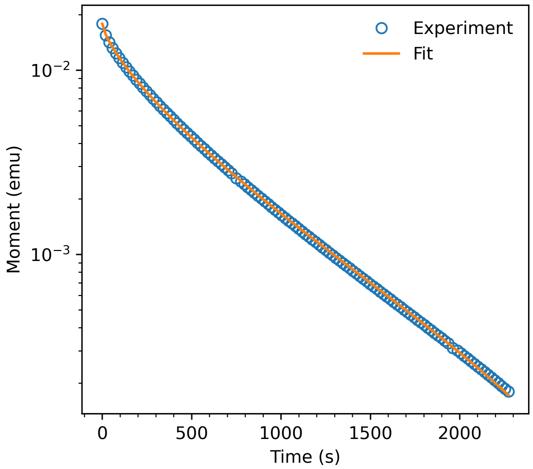

(d) 32 K

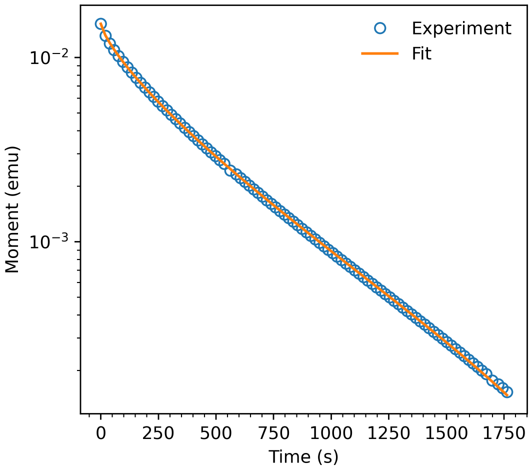

(e) 36 K

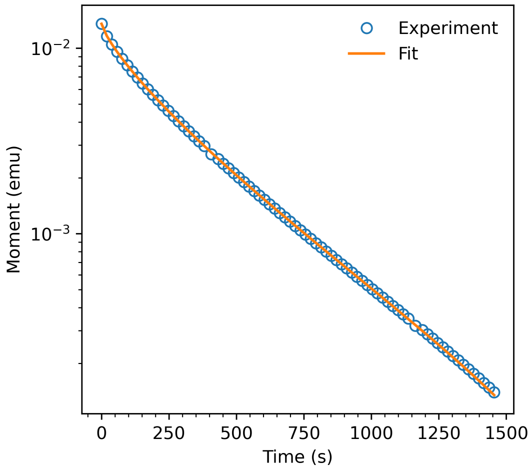

(f) 39 K

Figure S30: Zero-field DC Decay measurements for 200mM of $[Dy(Cp^{ttt})_2][B(C_6F_5)_4]$ dissolved in DCM

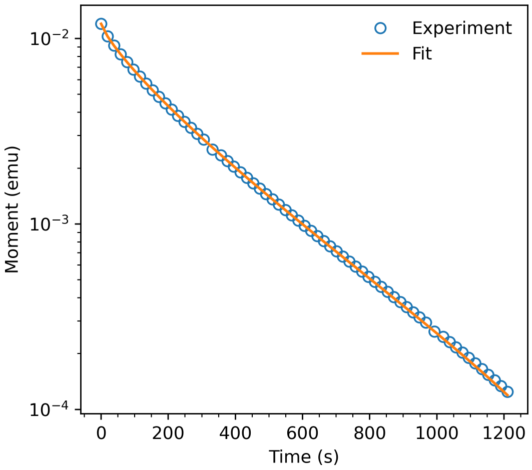

(a) 42 K

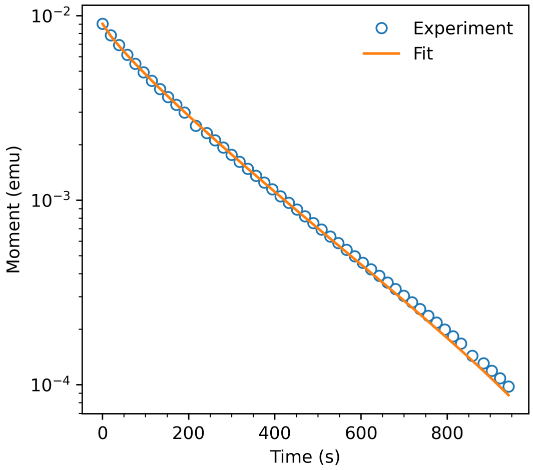

(b) 46 K

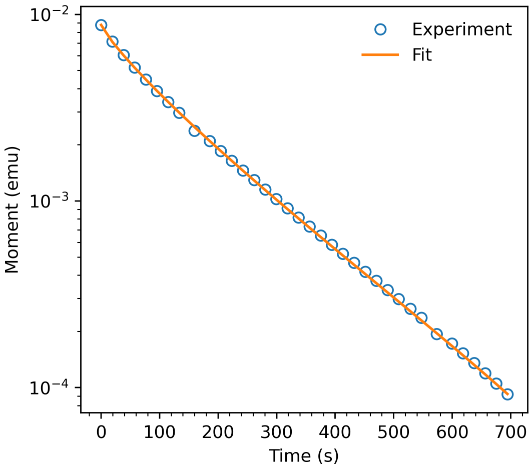

(c) 50 K

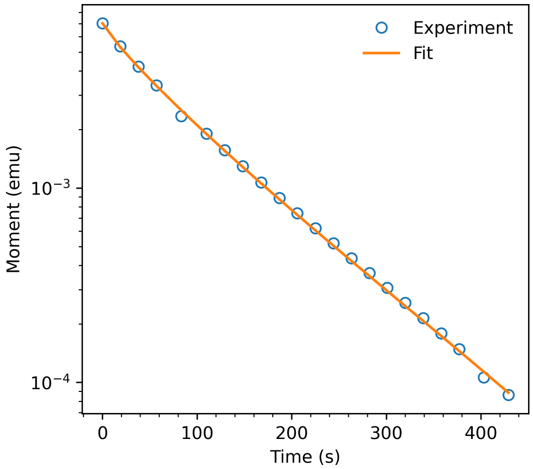

(d) 54 K

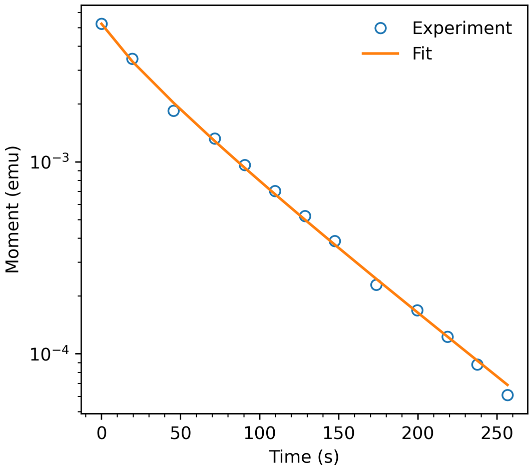

(e) 57 K

Figure S31: Zero-field DC Decay measurements for 100mM of $[Dy(Cp^{ttt})_2][B(C_6F_5)_4]$ dissolved in DCM

Relaxation Profiles

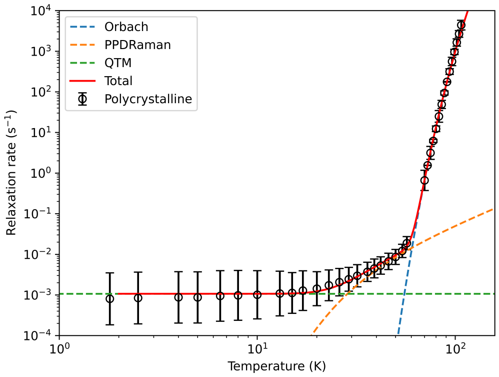

Figure S32: Relaxation profile of polycrystalline $[Dy(Cp^{ttt})_2][B(C_6F_5)_4]$ in zero-field fitted to Eq. S2.

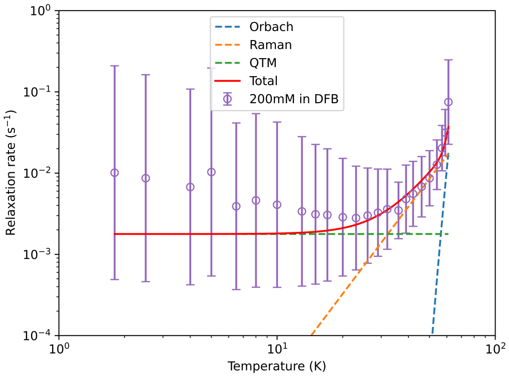

(a) 200mM in DFB

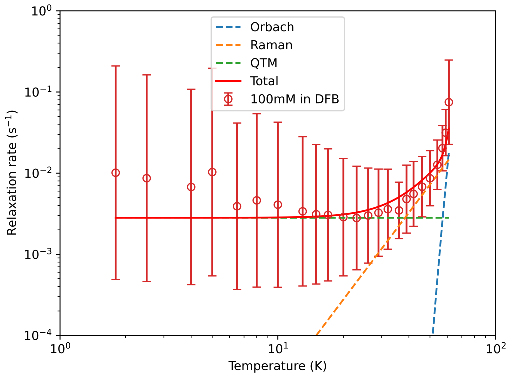

(b) 100 mM in DFB

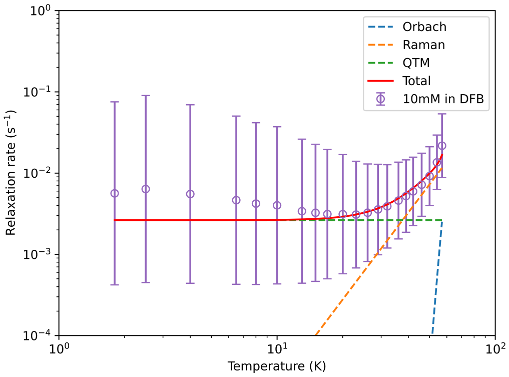

(c) 10 mM in DFB

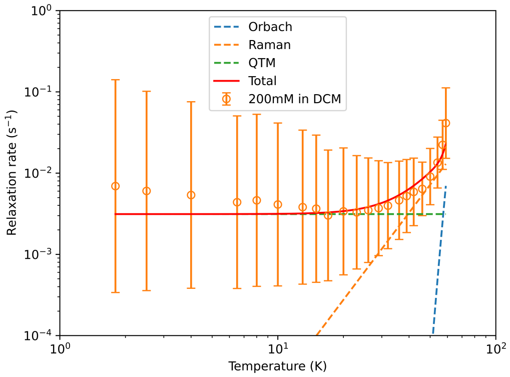

(d) 200 mM in DCM

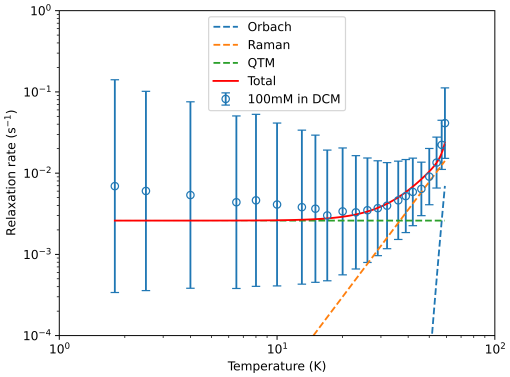

(e) 100mM in DCM

Figure S33: Zero-field relaxation profiles for $[Dy(Cp^{ttt})_2][B(C_6F_5)_4]$ dissolved in DFB and DCM

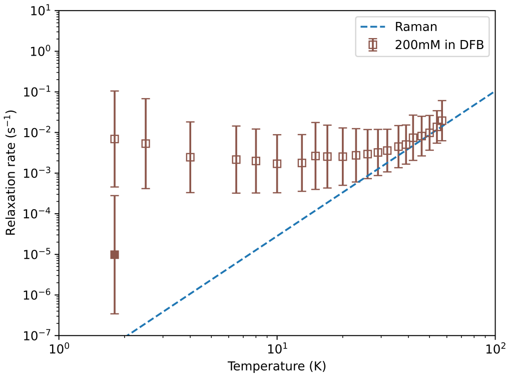

(a) 200mM in DFB

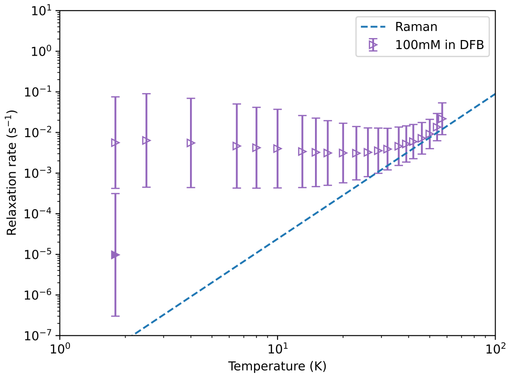

(b) 100 mM in DFB

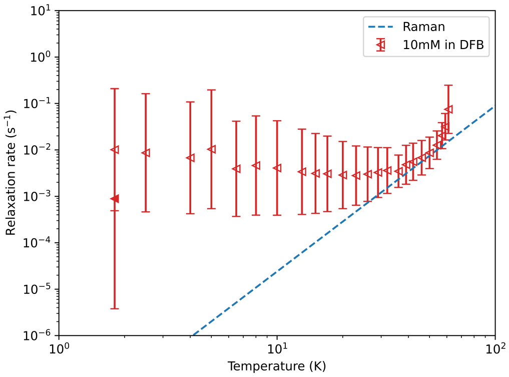

(c) 10 mM in DFB

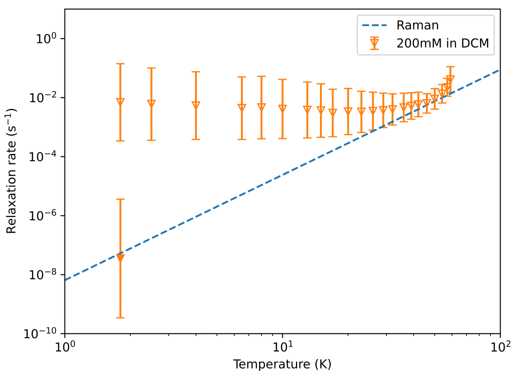

(d) 200mM in DCM

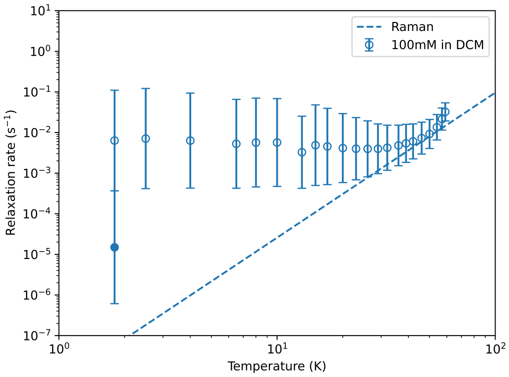

(e) 100mM in DCM

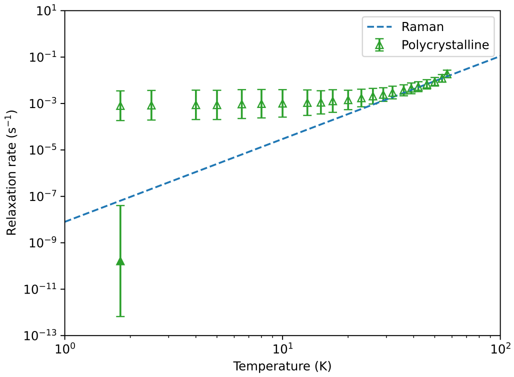

(f) Polycrystalline

Figure S34: Temperature-dependent relaxation profiles for solution phase $[Dy(Cp^{ttt})_2][B(C_6F_5)_4]$ showing the fits from Fig. 2 and Fig. S33 but extrapolating the power-law Raman-I model to lower temperatures and including the 1.8 K data point from the minimum of the field-dependent data.

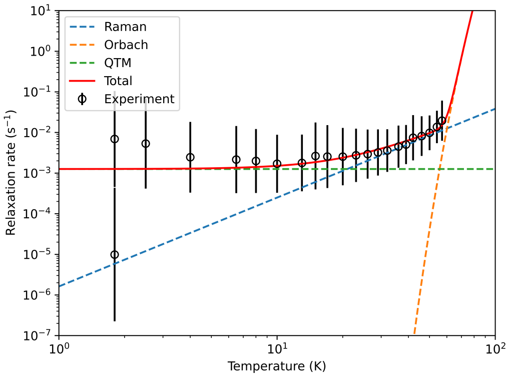

(a) 200mM in DFB power-law Raman

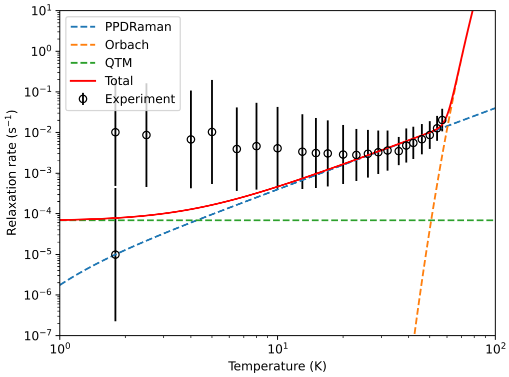

(b) 200mM in DFB PPD Raman

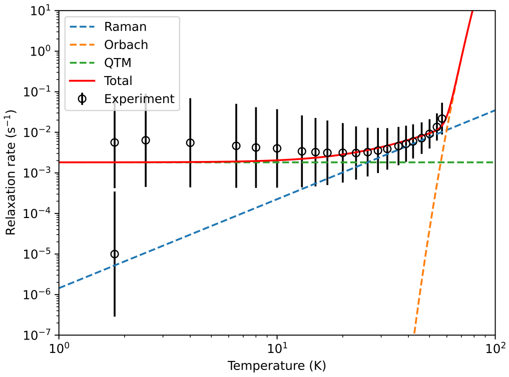

(c) 100 mM in DFB power-law Raman

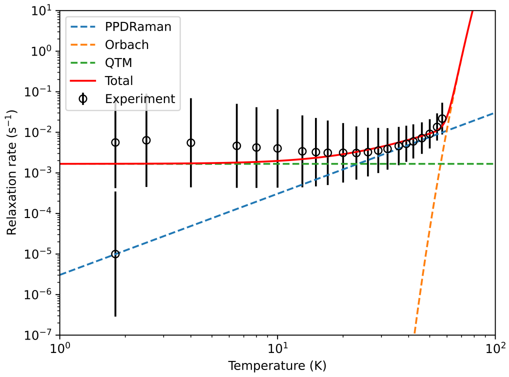

(d) 100 mM in DFB PPD Raman

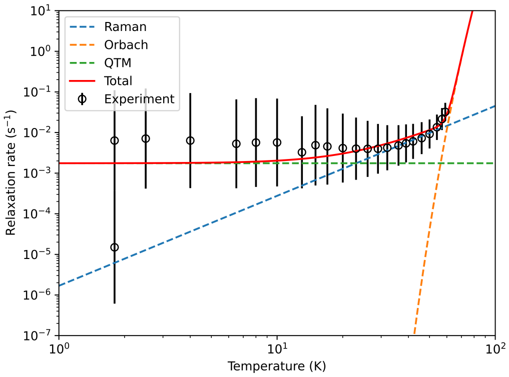

(e) 100mM in DCM power-law Raman

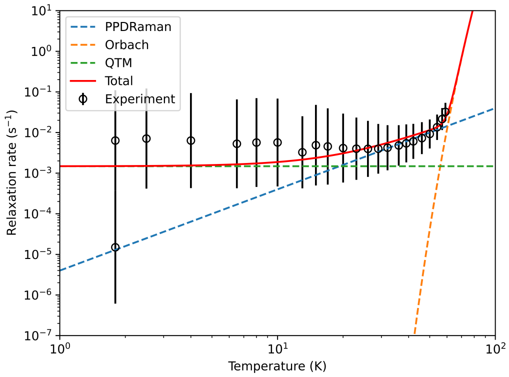

(f) 100mM in DCM PPD Raman

Figure S35: Temperature-dependent relaxation profiles for solution phase $[Dy(Cp^{ttt})_2][B(C_6F_5)_4]$ with the 1.8 K data point from the minimum of the field-dependent data being used to fit the power-law Raman-I parameters R and n (left column) and PPD Raman parameters R and w (right column).

Table S1: Parameters from fitting Eq. 3 to relaxation data of solution phase samples of $[Dy(Cp^{ttt})_2][B(C_6F_5)_4]$, including an additional 1.8 K data point from the minimum of the field-dependent data. The Orbach parameters were kept fixed to the zero-field fits of the polycrystalline sample. Parentheses indicate the one estimated standard deviation of the distribution of the parameters.

Sample	U_{eff} K	A Log[s]	n	R Log[$s^{-1}K^{-n}$]	Q Log[s]
200 mM DFB	1706	-10.40	2.10(16)	-5.79(27)	2.90(12)
100 mM DFB	1706	-10.40	2.20(20)	-5.85(35)	2.74(11)
100 mM DCM	1706	-10.40	2.24(28)	-5.79(50)	2.76(24)

$$\log_{10}\left[\tau^{-1}(T)\right] = \log_{10}\left[10^{-A}\exp\left[-U_{\mathrm{eff}}/T\right] + 10^{R}\frac{\exp\left[\hbar\omega_{\alpha}/k_{\mathrm{B}}T\right]}{\left(\exp\left[\hbar\omega_{\alpha}/k_{\mathrm{B}}T\right]-1\right)^{2}} + 10^{-Q}\right] \quad \text{(S2)}$$

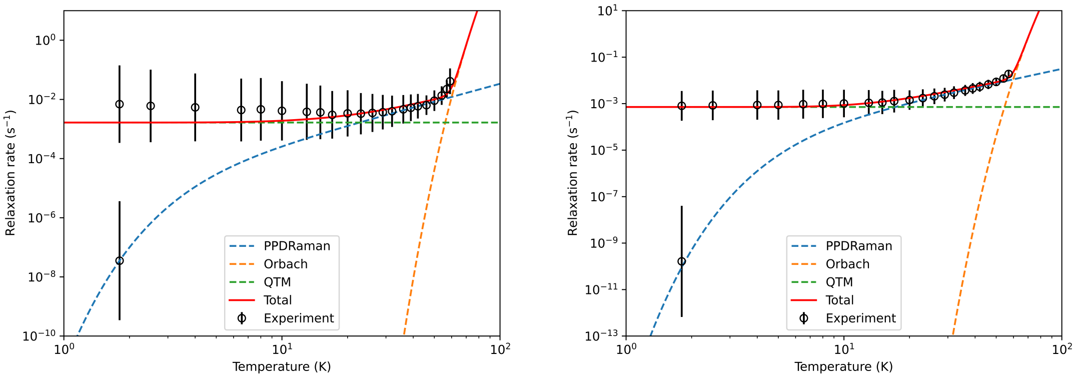

(a) 200mM in DCM

(b) Polycrystalline

Figure S36: Temperature-dependent relaxation profiles for polycrystalline and 200 mM DCM $[Dy(Cp^{ttt})_2][B(C_6F_5)_4]$ samples with the 1.8 K data point from the minimum of the field-dependent data being used to fit the zero-field PPD-Raman parameters R and ω.

Table S2: Parameters from fitting Eq. S2 to relaxation data of polycrystalline and solution phase samples of $[Dy(Cp^{ttt})_2][B(C_6F_5)_4]$, including an additional 1.8 K data point from the minimum of the field-dependent data. The Orbach parameters were kept fixed to the zero-field fits of the polycrystalline sample. Parentheses indicate the one estimated standard deviation of the distribution of the parameters.

Sample	U_{eff} K	A Log[s]	ω (K)	R Log[s^{-1}K^{-n}]	Q Log[s]
poly-crystalline	1706	-10.40	31.2(3.6)	-2.51(11)	3.15(7)
200 mM DCM	1706	-10.40	18.8(3.3)	-2.92(17)	2.78(19)

Determining M_{eq} for infield measurements

The relaxation times for infield measurements of $[Dy(Cp^{ttt})_2][B(C_6F_5)_4]$ are so long that it is not feasible to measure the decay curve to equilibrium. Furthermore, in this case it was not possible to determine the mass of sample in solution. To fix M_{eq}, the equilibrium magnetisation was calculated in PHI using the crystal field parameters for $[Dy(Cp^{ttt})_2][B(C_6F_5)_4]$ calculated by Goodwin *et al*[S2] (Table. S3).

Table S3: PHI input file used to calculate the equilibrium magnetisation for $[\mathbf{Dy(Cp^{ttt})_2}][\mathbf{B(C_6F_5)_4}]$.

```
****Ion
Dy(J)
***CrystalField
1 2 0 1226.9
1 2 1 -124.4
1 2 2 -5.2
1 4 -3 0.1
1 4 0 31.2
1 4 1 -11.3
1 4 2 -40.5
1 4 3 -123.5
1 4 4 42.1
1 6 -5 0.1
1 6 -3 0.1
1 6 -2 -0.1
1 6 0 -36
1 6 1 108.9
1 6 2 198.3
1 6 3 -141.1
1 6 4 -86
1 6 5 -23.8
1 6 6 -5.2
***Mag
Field Powder 3
TMag 1.8
Sweep 0 3 3001
***Params
OpMode Sim M
***end
```

The resultant magnetisation curve was calibrated to each measurements using the exper-

imental saturation magnetisation at 3 T:

$$M_{\text{calibrated}} = M_{\text{calc}} \times \frac{M_{\text{exp sat}}}{M_{\text{calc sat}}}. \tag{S3}$$

and M_{eq} was extracted for each decay from the calibrated curves.

Infield decays

Infield DC decay measurements of $[Dy(Cp^{ttt})_2][B(C_6F_5)_4]$ were modelled using Eq. 1, with M_0 fixed to the first data point at measured zero-field, and M_{eq} fixed to a value calculated using Eq. S3 (see previous section).[S1]

Polycrystalline

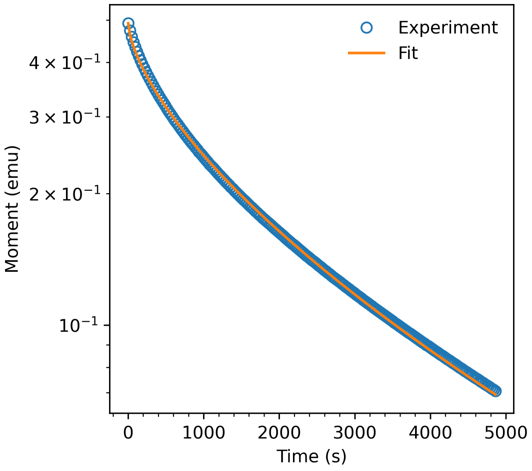

(a) 10 Oe

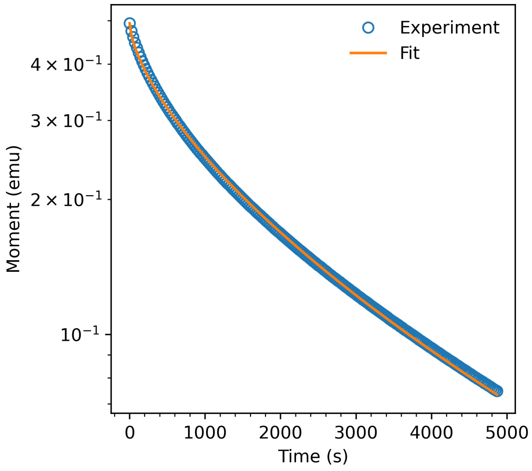

(b) 20 Oe

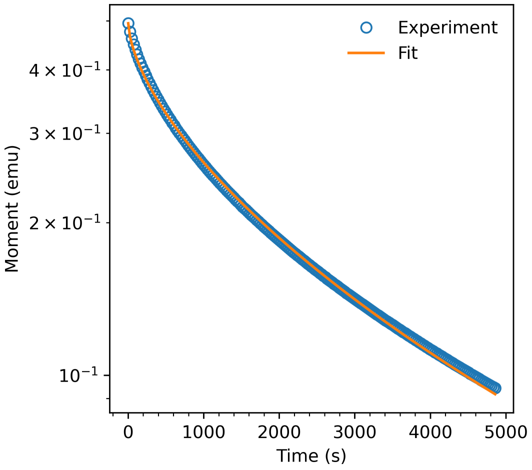

(c) 50 Oe

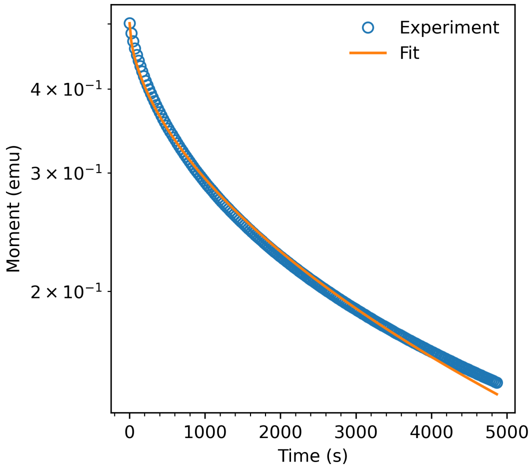

(d) 100 Oe

Figure S37: Infield DC Decay measurements polycrystalline $[Dy(Cp^{ttt})_2][B(C_6F_5)_4]$

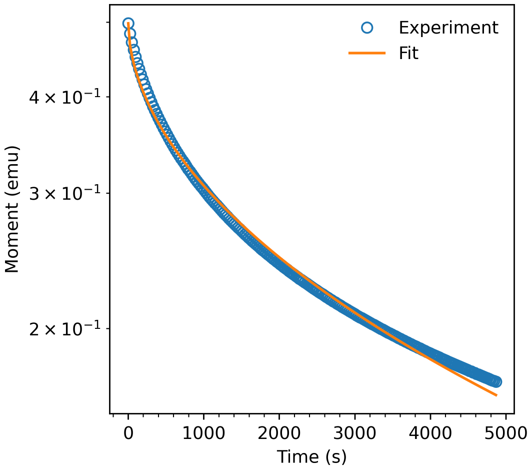

(a) 120 Oe

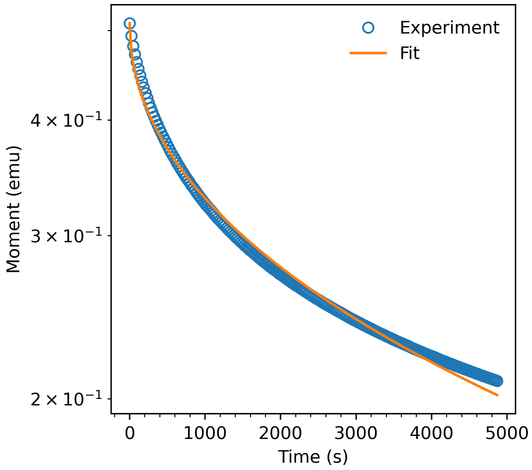

(b) 150 Oe

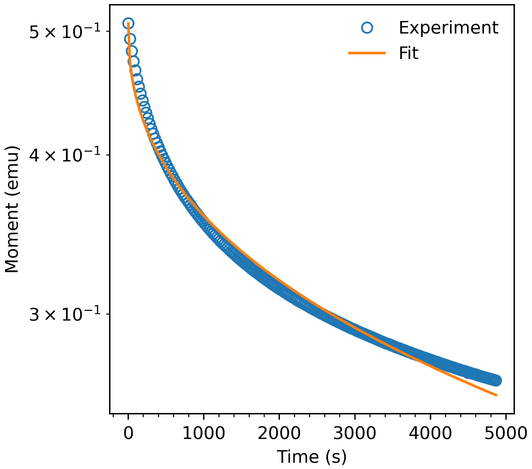

(c) 200 Oe

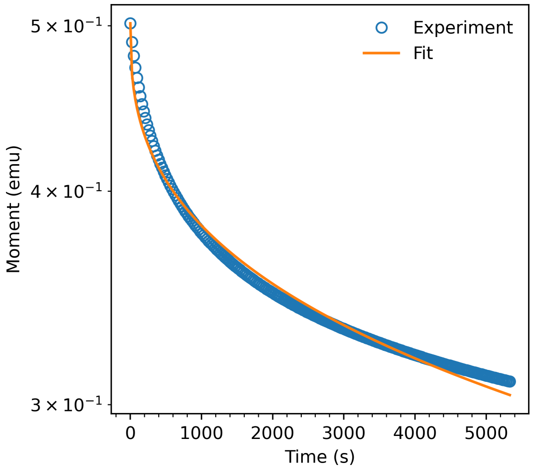

(d) 250 Oe

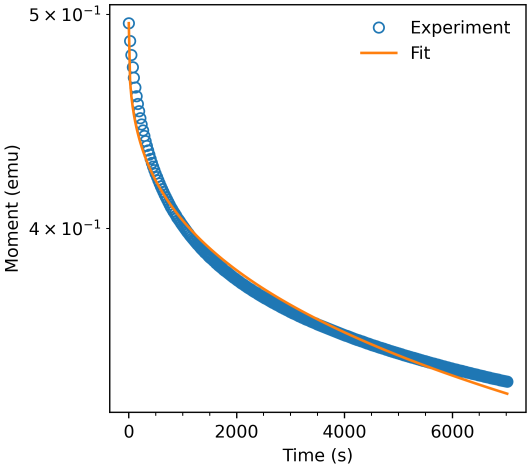

(e) 300 Oe

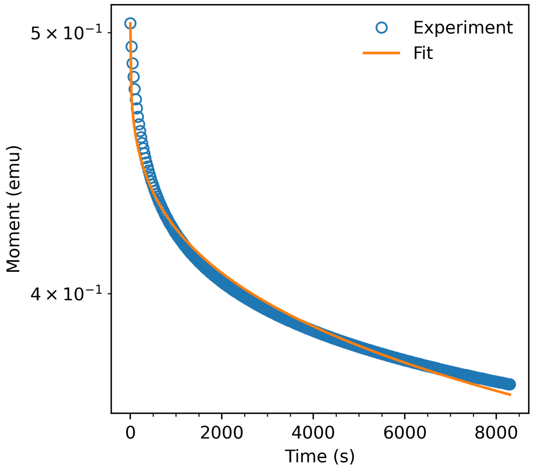

(f) 350 Oe

Figure S38: Infield DC Decay measurements of polycrystalline $[Dy(Cp^{ttt})_2][B(C_6F_5)_4]$

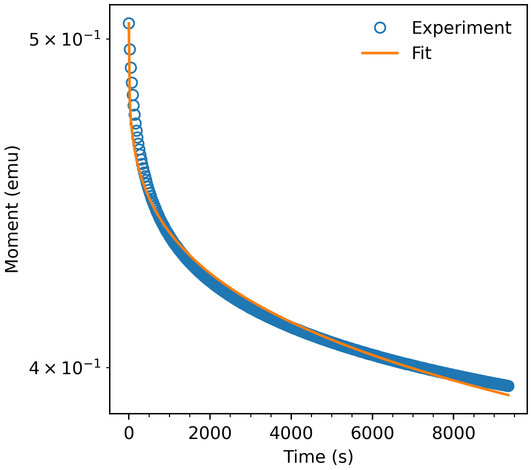

(a) 400 Oe

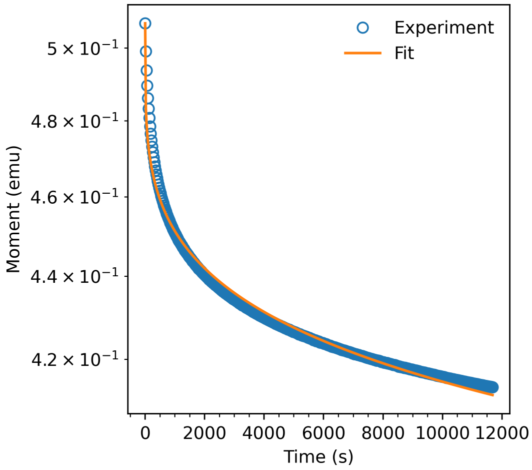

(b) 450 Oe

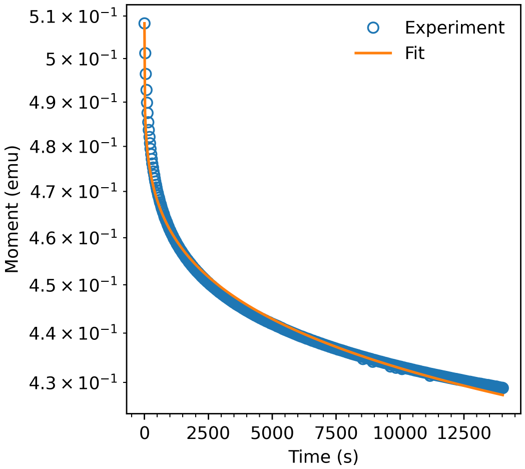

(c) 500 Oe

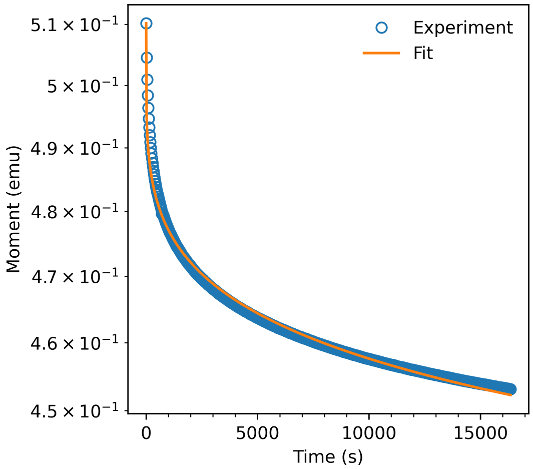

(d) 600 Oe

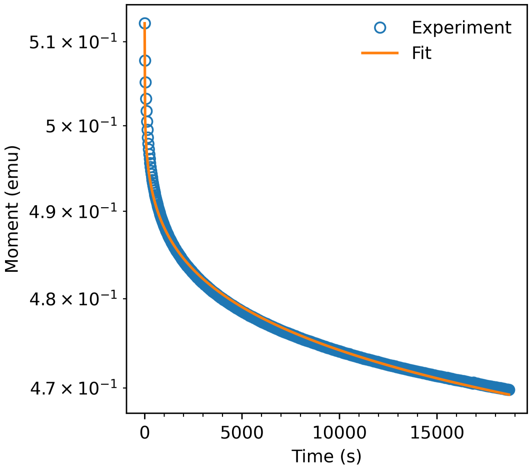

(e) 700 Oe

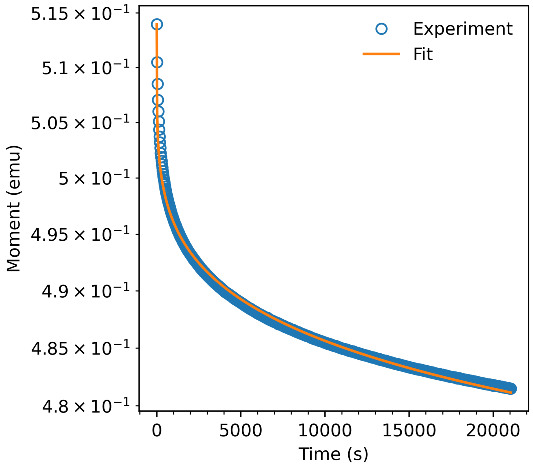

(f) 800 Oe

Figure S39: Infield DC Decay measurements of polycrystalline $[Dy(Cp^{ttt})_2][B(C_6F_5)_4]$

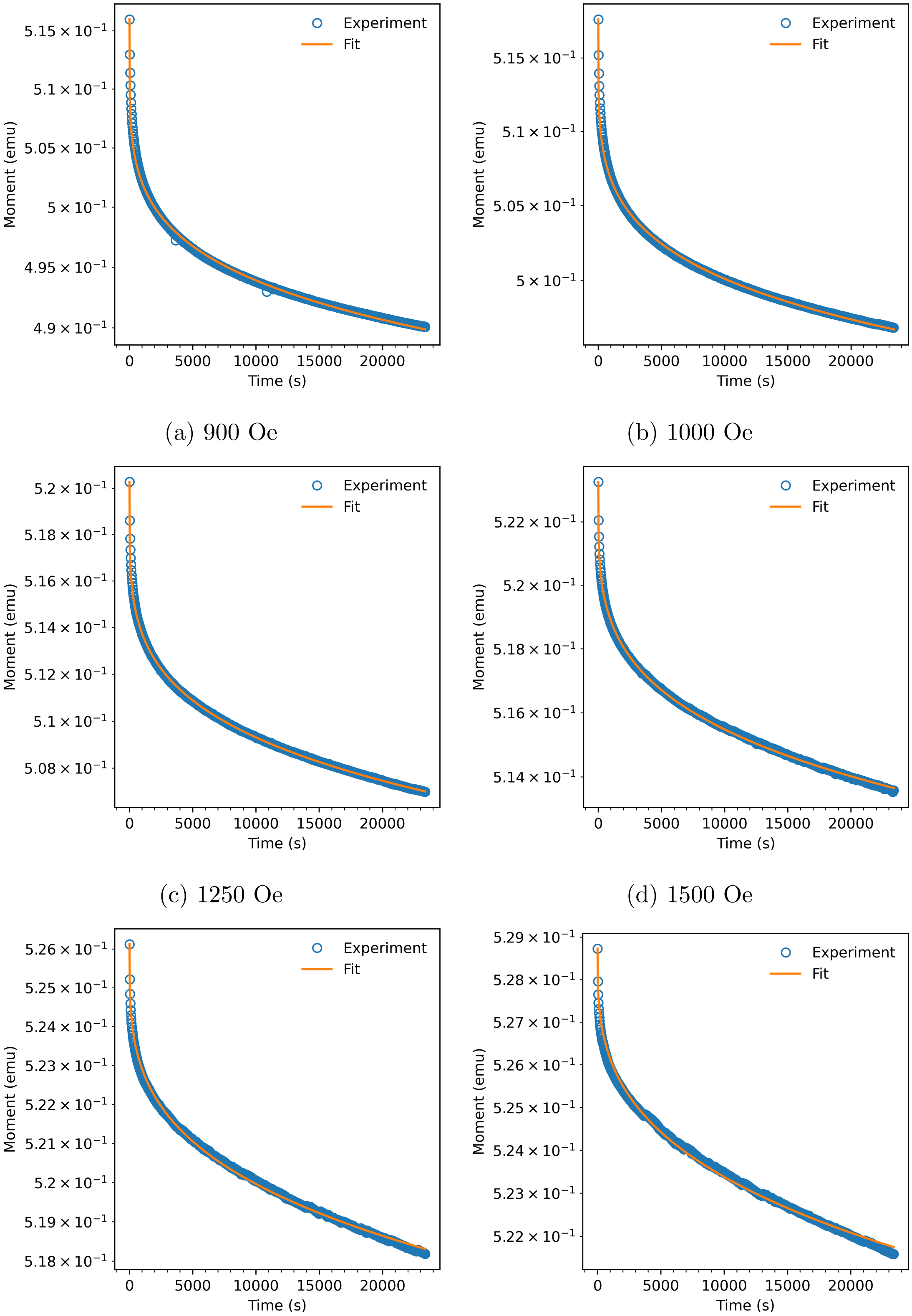

(a) 900 Oe

(b) 1000 Oe

(c) 1250 Oe

(d) 1500 Oe

(e) 1750 Oe

(f) 2000 Oe

Figure S40: Infield DC Decay measurements of polycrystalline $[Dy(Cp^{ttt})_2][B(C_6F_5)_4]$

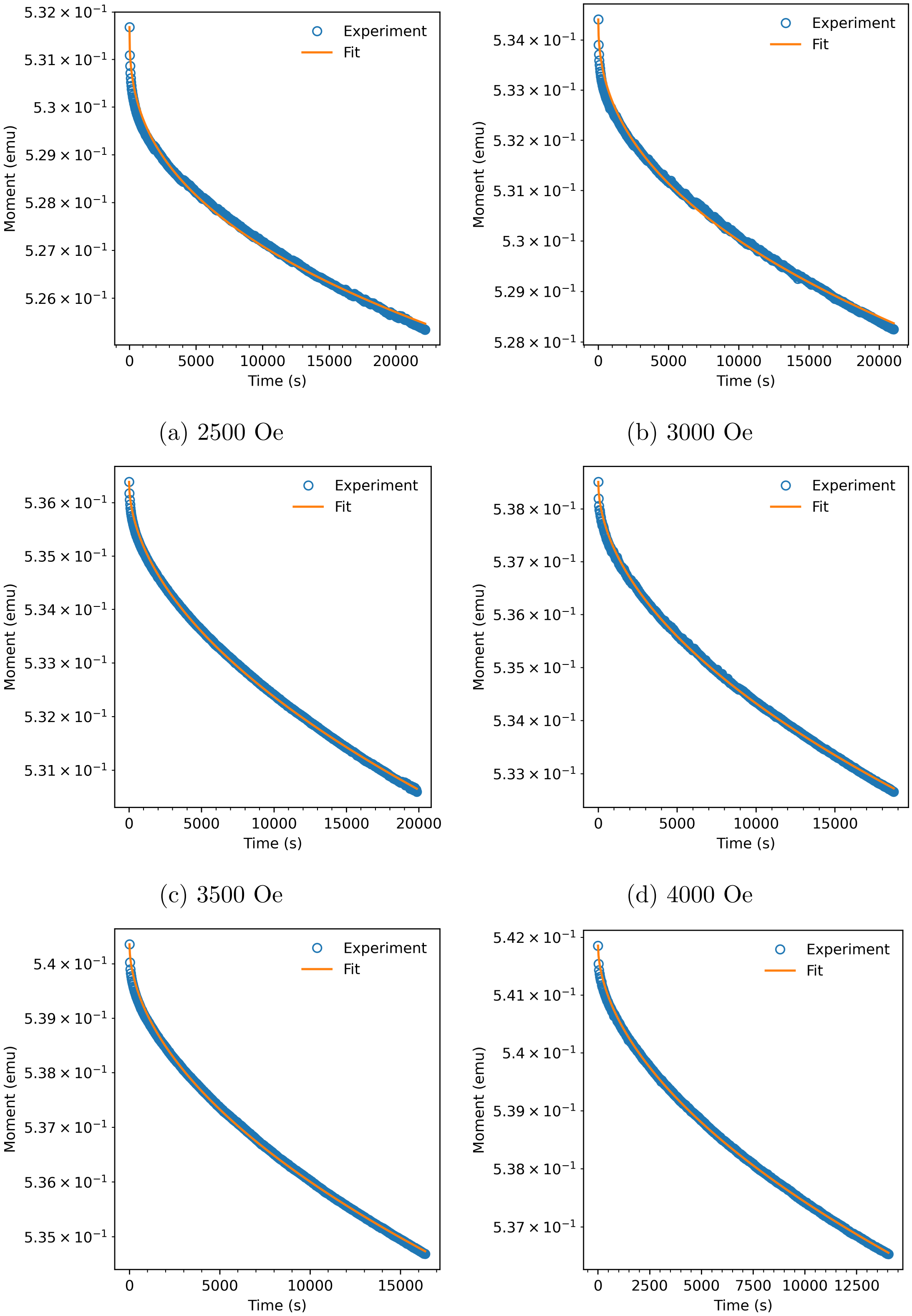

(a) 2500 Oe

(b) 3000 Oe

(c) 3500 Oe

(d) 4000 Oe

(e) 4500 Oe

(f) 5000 Oe

Figure S41: Infield DC Decay measurements of polycrystalline $[Dy(Cp^{ttt})_2][B(C_6F_5)_4]$

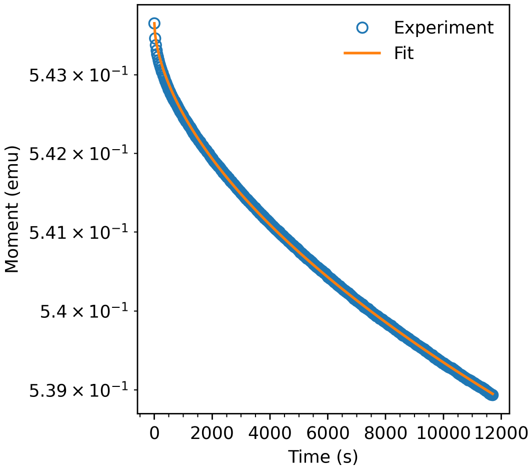

(a) 6000 Oe

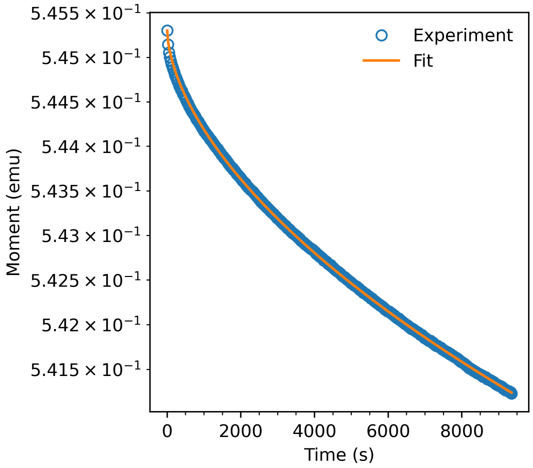

(b) 7000 Oe

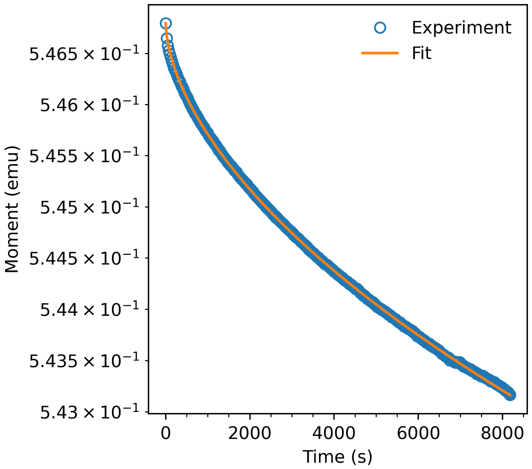

(c) 8000 Oe

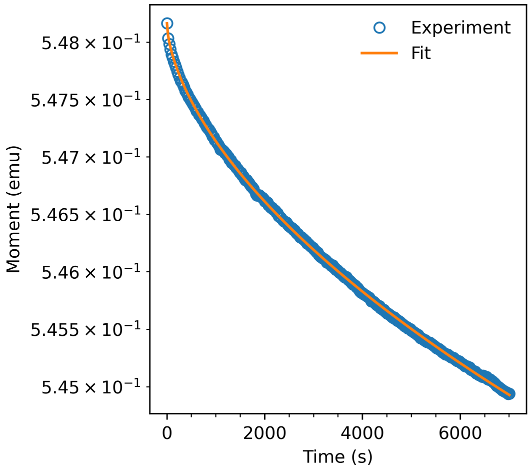

(d) 9000 Oe

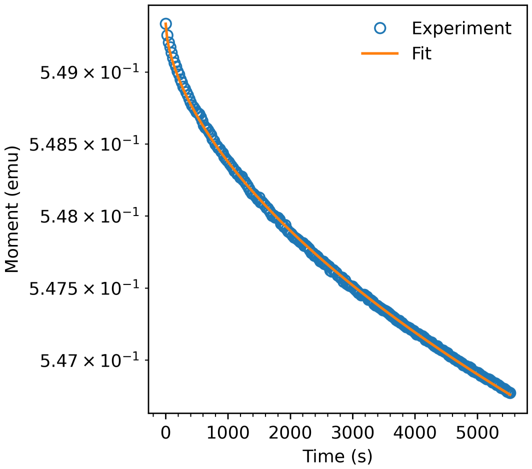

(e) 10000 Oe

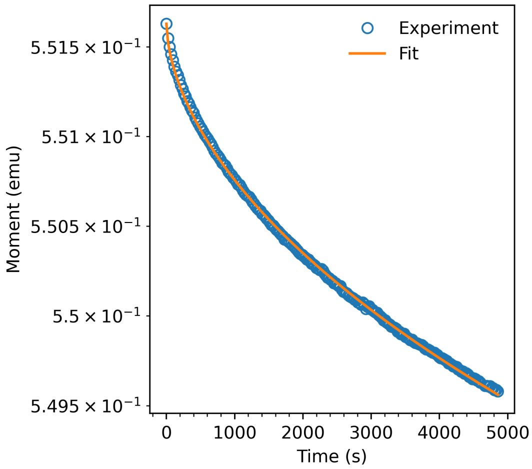

(f) 12500 Oe

Figure S42: Infield DC Decay measurements of polycrystalline $[Dy(Cp^{ttt})_2][B(C_6F_5)_4]$

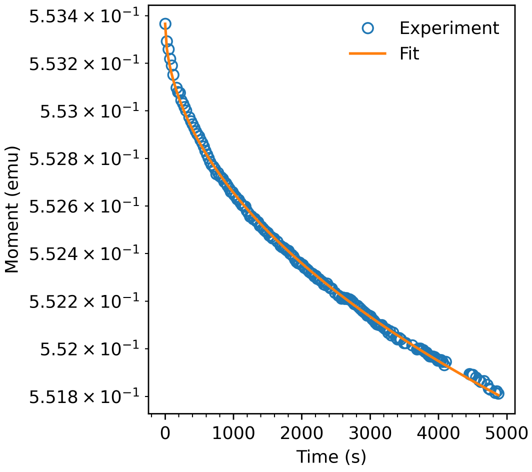

(a) 15000 Oe

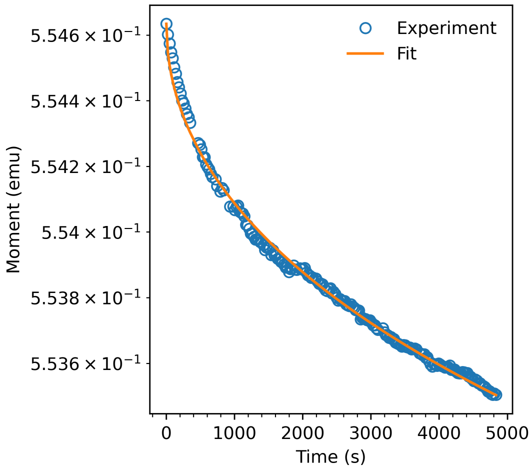

(b) 17500 Oe

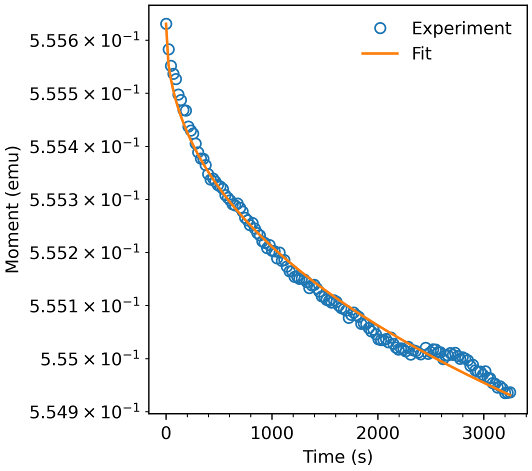

(c) 20000 Oe

Figure S43: Infield DC Decay measurements of polycrystalline $[Dy(Cp^{ttt})_2][B(C_6F_5)_4]$

DFB

200mM

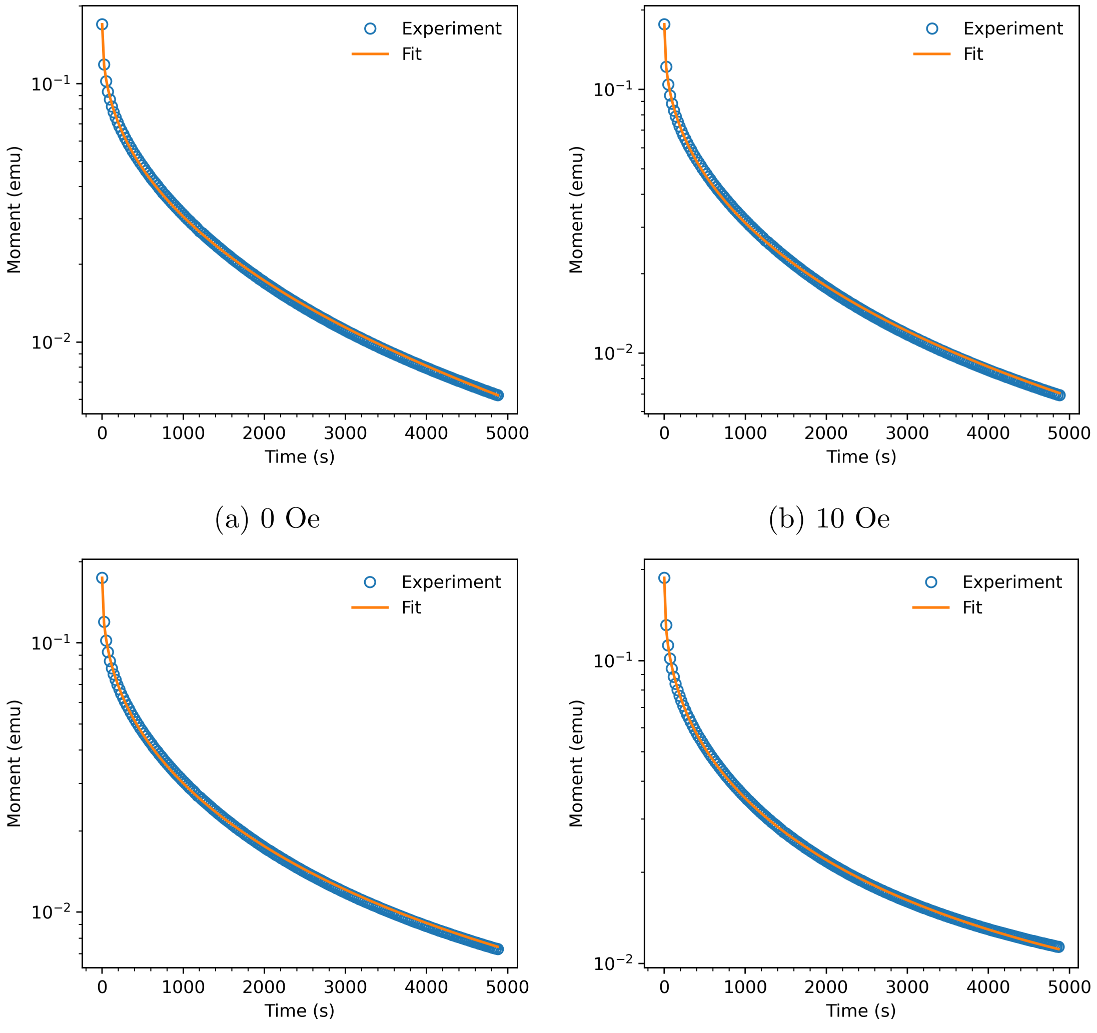

(a) 0 Oe

(b) 10 Oe

(c) 20 Oe

(d) 50 Oe

Figure S44: Infield DC Decay measurements for 200mM of $[Dy(Cp^{ttt})_2][B(C_6F_5)_4]$ dissolved in DFB performed at 1.8 K

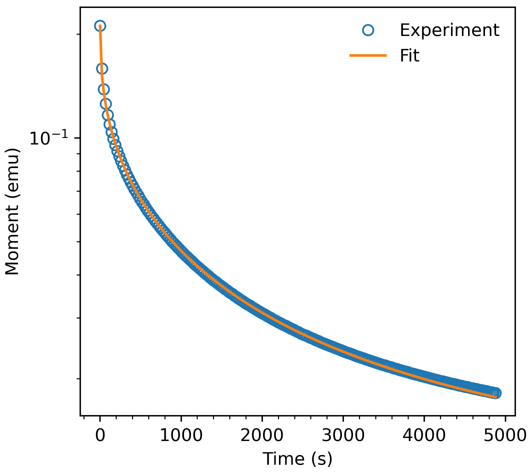

(a) 100 Oe

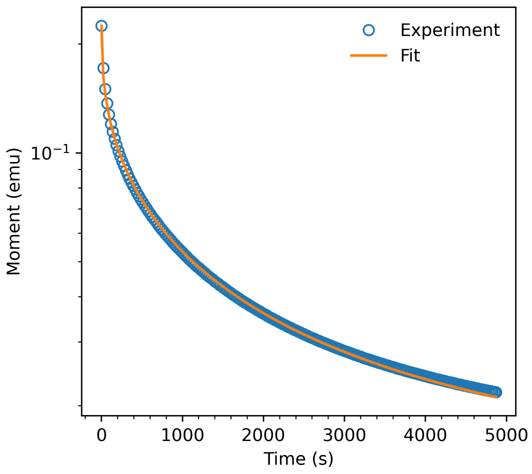

(b) 120 Oe

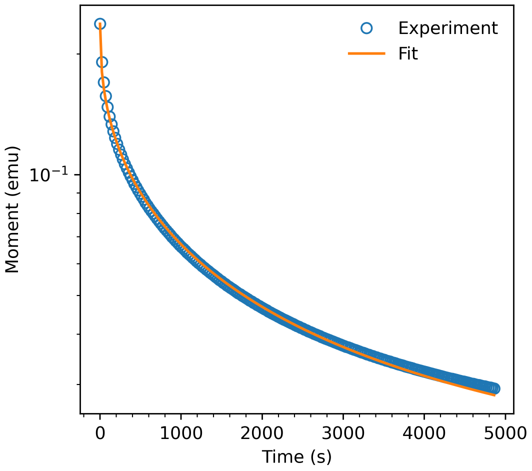

(c) 150 Oe

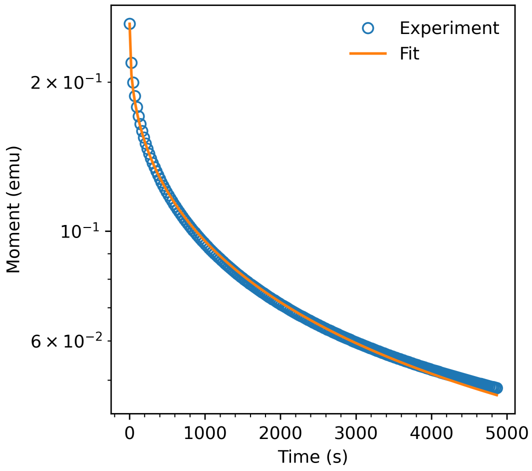

(d) 200 Oe

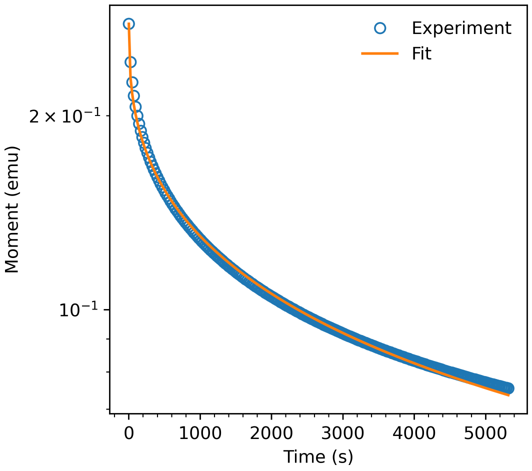

(e) 250 Oe

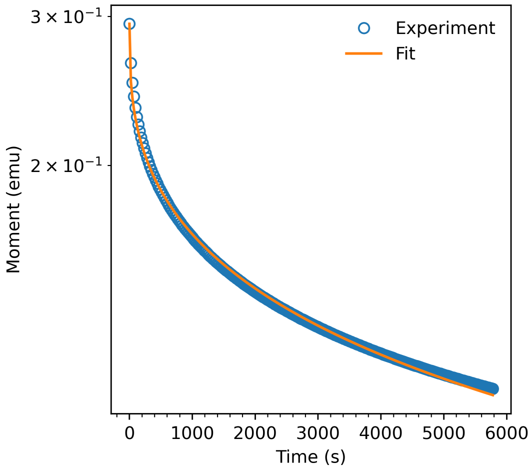

(f) 300 Oe

Figure S45: Infield DC Decay measurements for 200mM of $[Dy(Cp^{ttt})_2][B(C_6F_5)_4]$ dissolved in DFB performed at 1.8 K

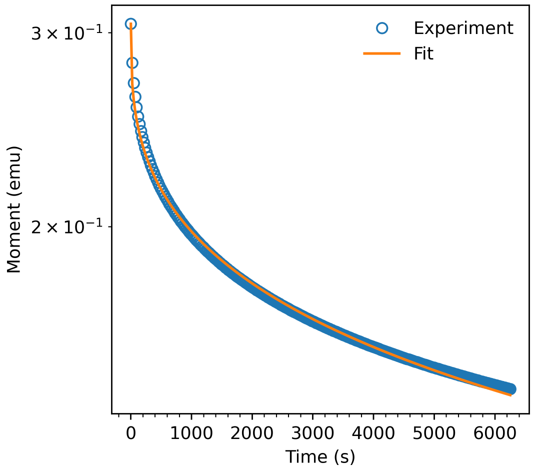

(a) 350 Oe

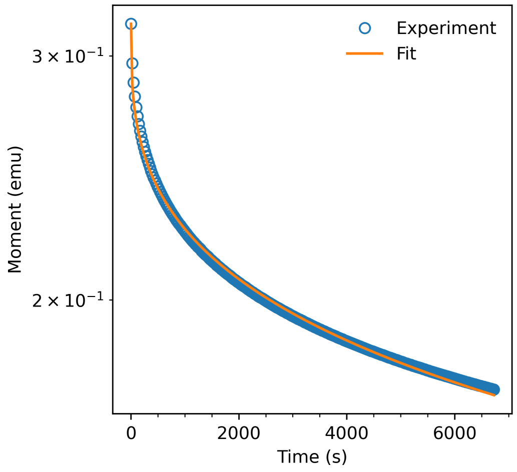

(b) 400 Oe

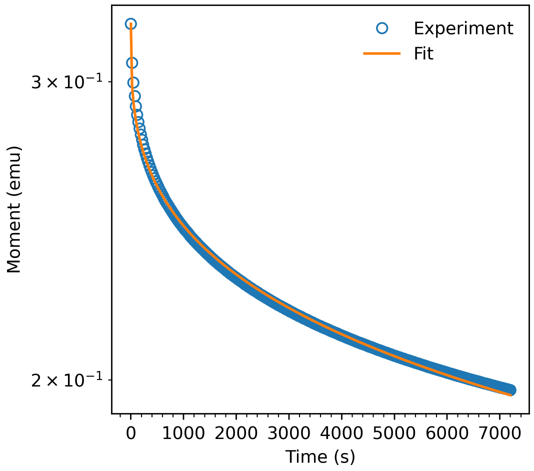

(c) 450 Oe

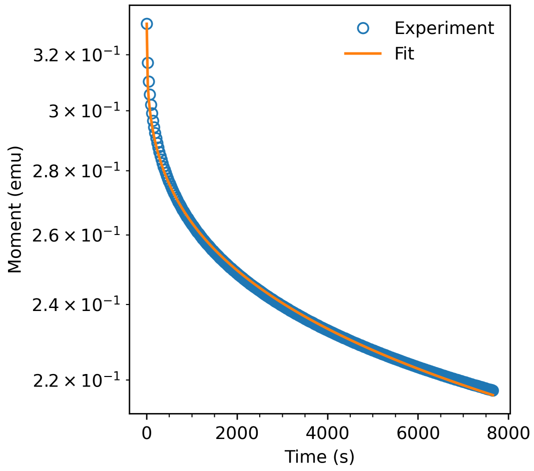

(d) 500 Oe

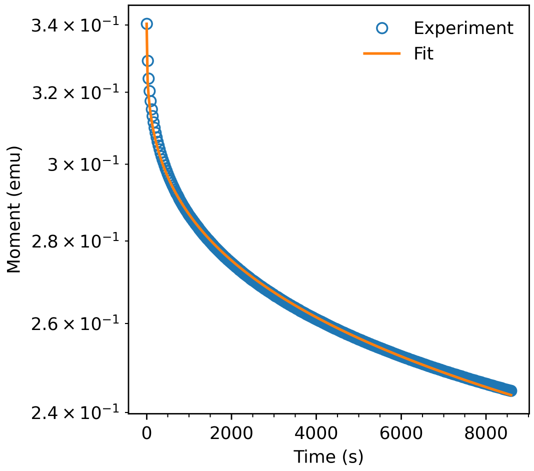

(e) 600 Oe

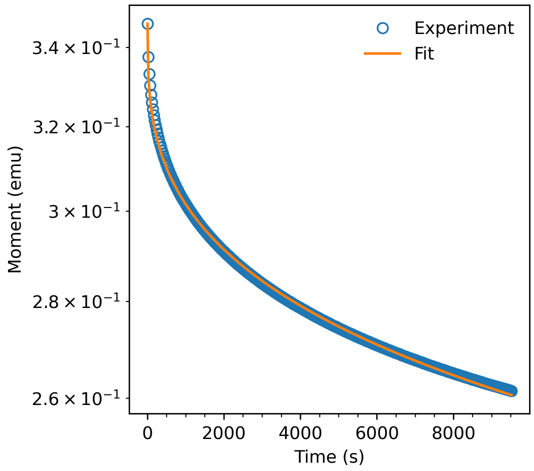

(f) 700 Oe

Figure S46: Infield DC Decay measurements for 200mM of $[Dy(Cp^{ttt})_2][B(C_6F_5)_4]$ dissolved in DFB performed at 1.8 K

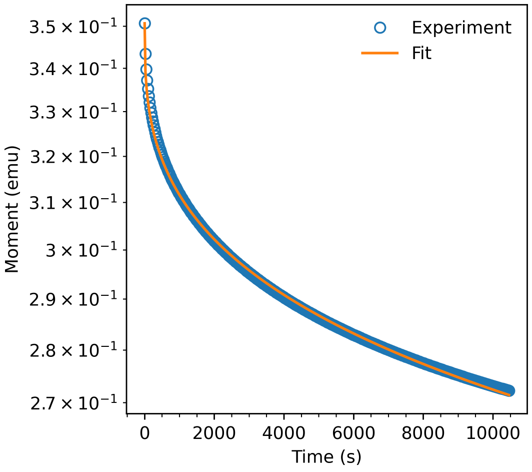

(a) 800 Oe

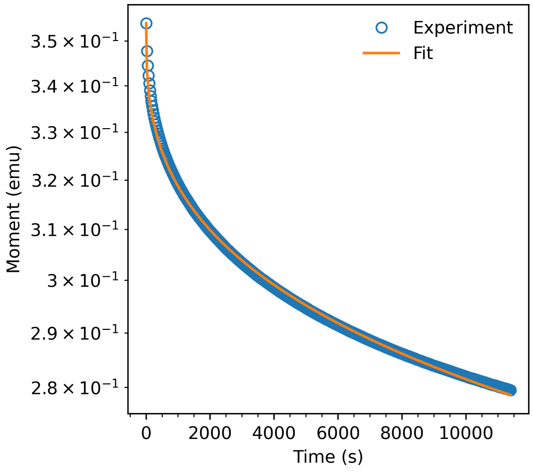

(b) 900 Oe

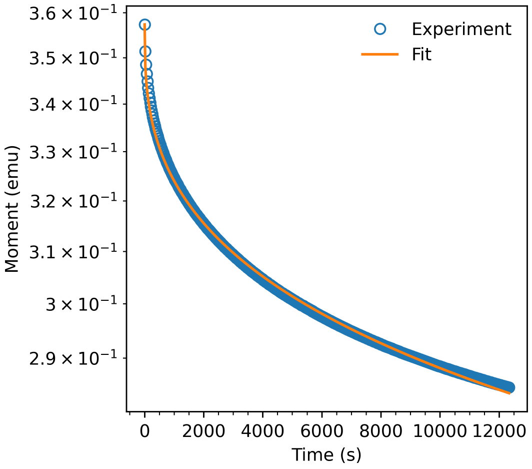

(c) 1000 Oe

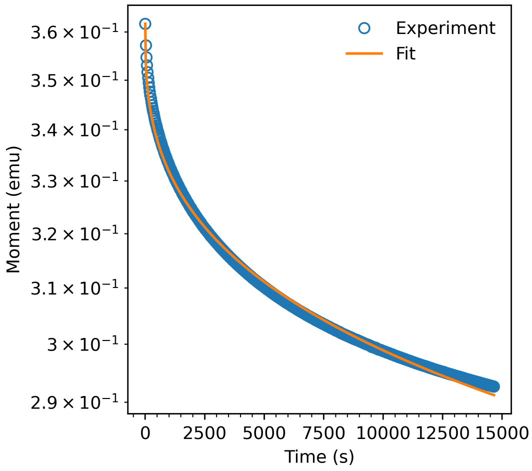

(d) 1250 Oe

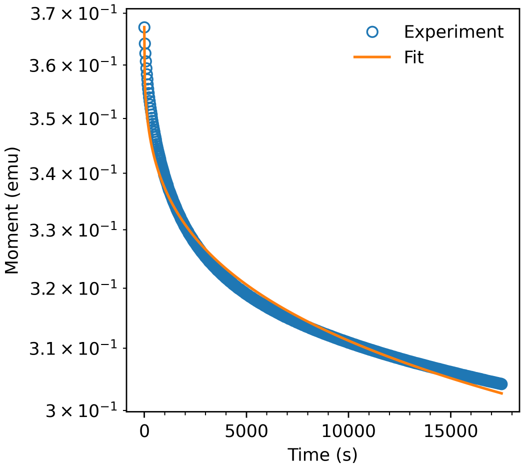

(e) 2000 Oe

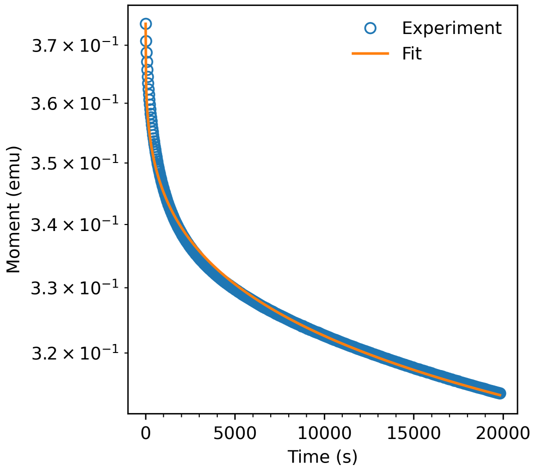

(f) 2500 Oe

Figure S47: Infield DC Decay measurements for 200mM of $[Dy(Cp^{ttt})_2][B(C_6F_5)_4]$ dissolved in DFB performed at 1.8 K

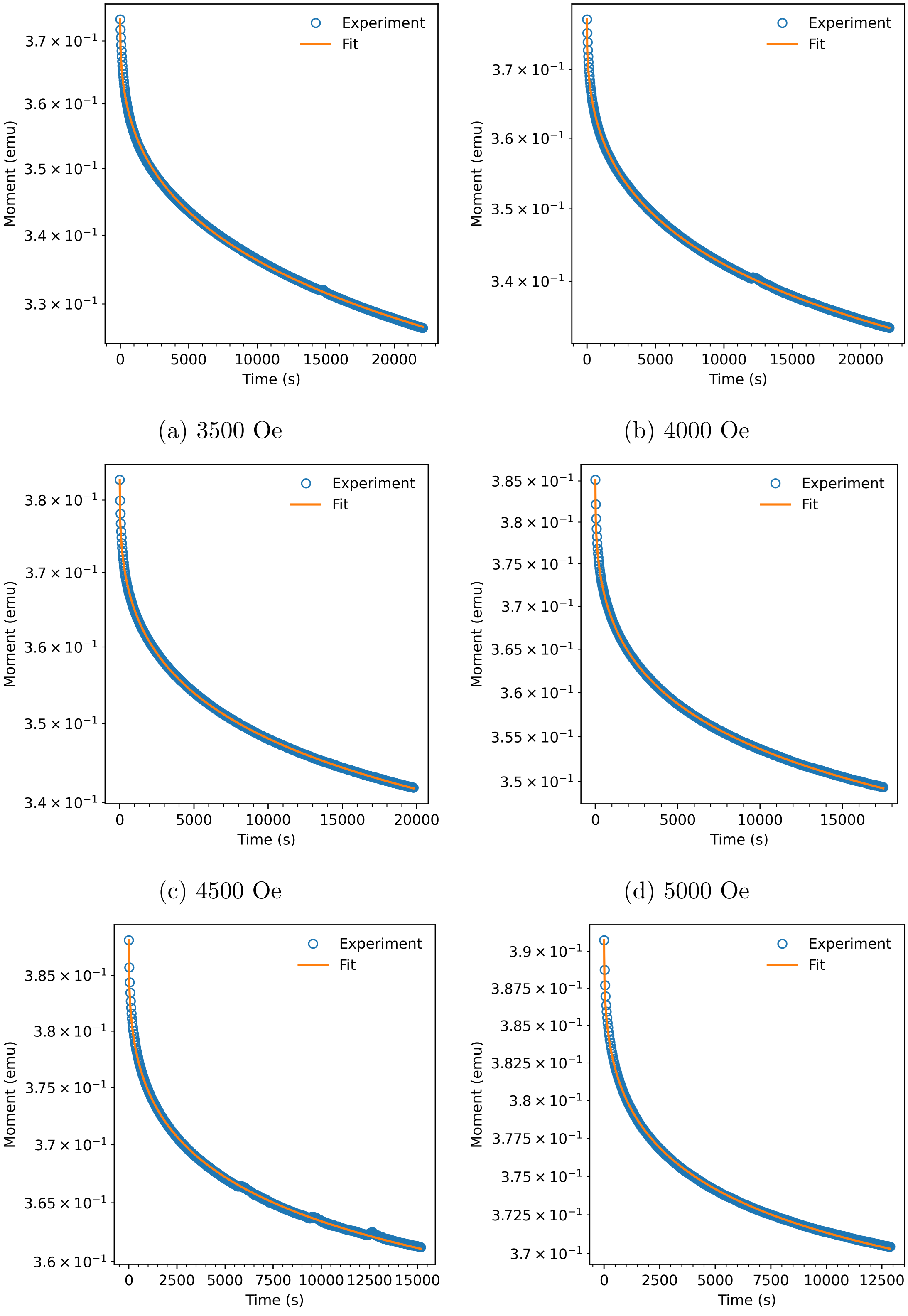

(a) 3500 Oe (b) 4000 Oe

(c) 4500 Oe (d) 5000 Oe

(e) 6000 Oe (f) 7000 Oe

Figure S48: Infield DC Decay measurements for 200mM of $[Dy(Cp^{ttt})_2][B(C_6F_5)_4]$ dissolved in DFB performed at 1.8 K

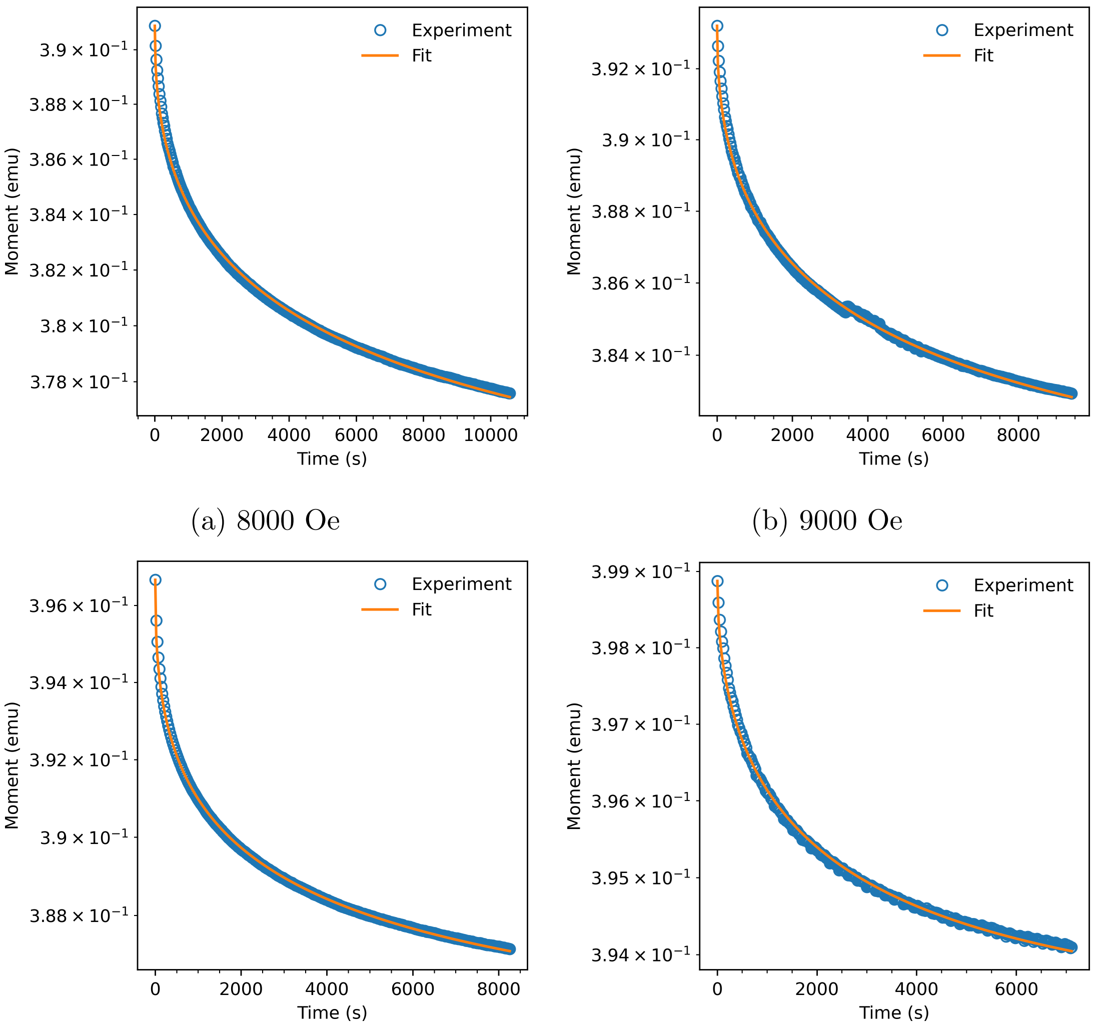

(a) 8000 Oe

(b) 9000 Oe

(c) 10000 Oe

(d) 12500 Oe

Figure S49: Infield DC Decay measurements for 200mM of $[Dy(Cp^{ttt})_2][B(C_6F_5)_4]$ dissolved in DFB performed at 1.8 K

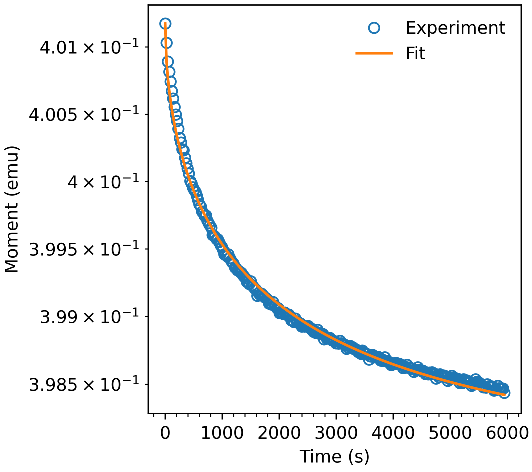

(a) 15000 Oe

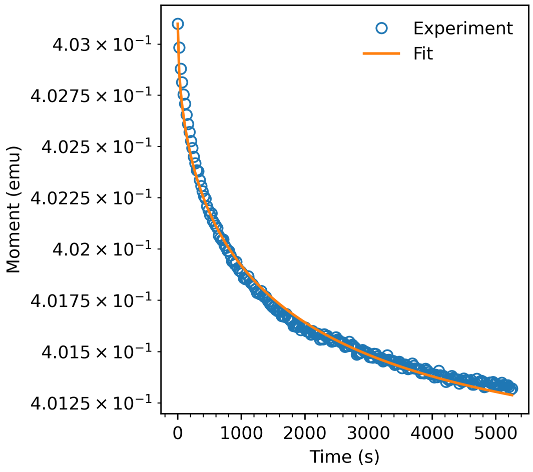

(b) 17500 Oe

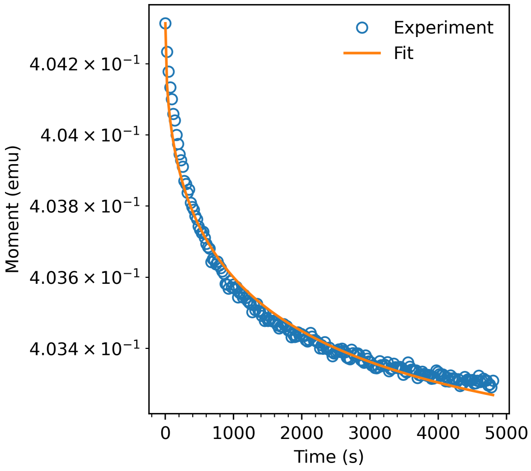

(c) 20000 Oe

Figure S50: Infield DC Decay measurements for 200mM of $[Dy(Cp^{ttt})_2][B(C_6F_5)_4]$ dissolved in DFB performed at 1.8 K

100mM

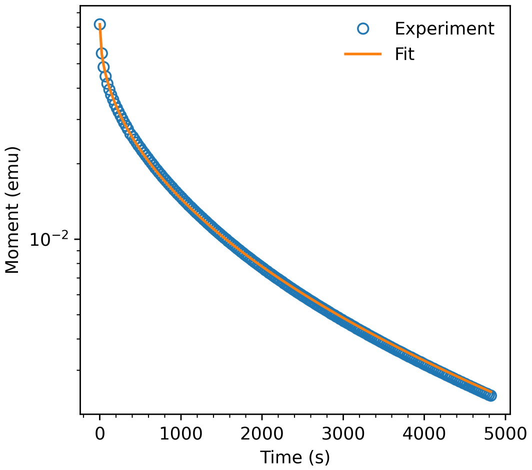

(a) 0 Oe

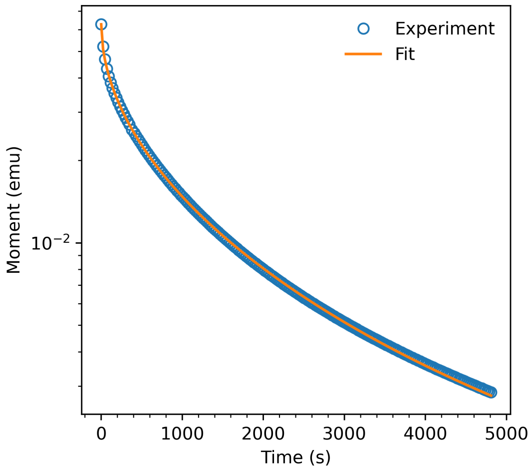

(b) 10 Oe

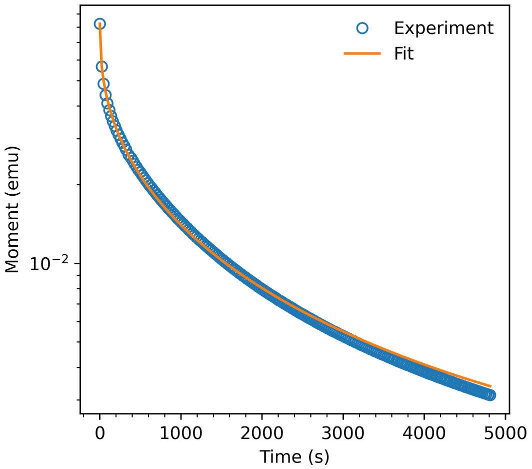

(c) 20 Oe

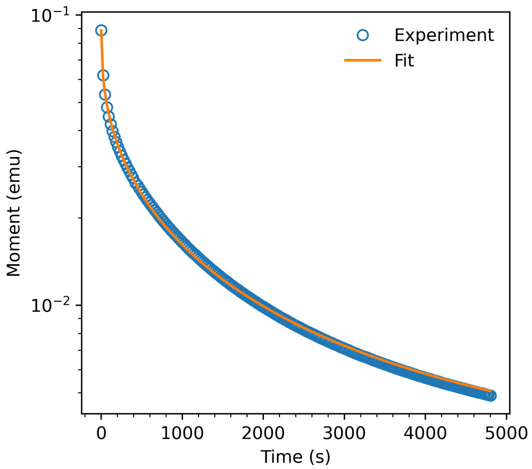

(d) 50 Oe

Figure S51: Infield DC Decay measurements for 100mM of $[Dy(Cp^{ttt})_2][B(C_6F_5)_4]$ dissolved in DFB performed at 1.8 K

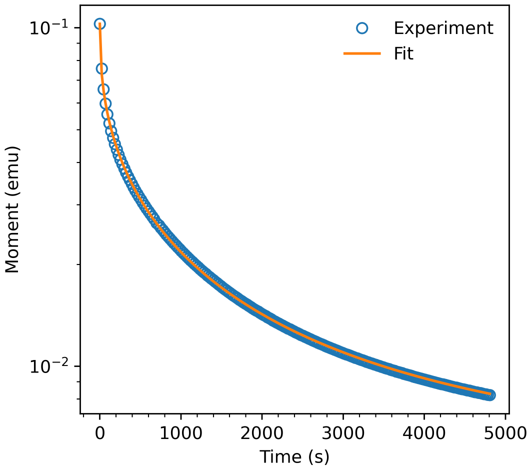

(a) 100 Oe

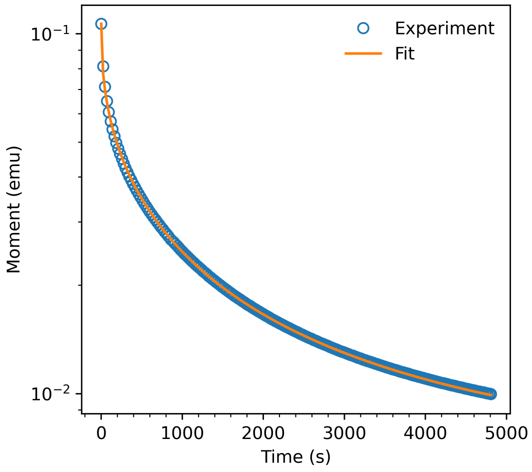

(b) 120 Oe

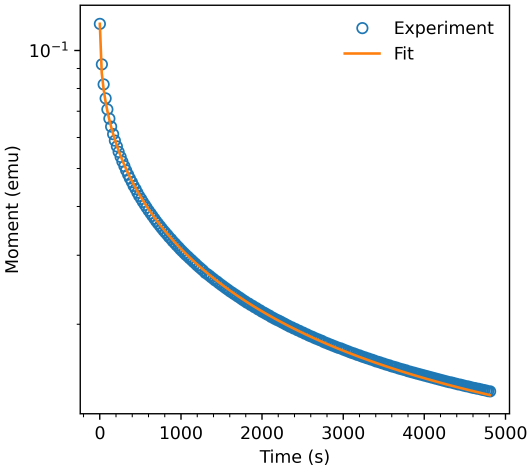

(c) 150 Oe

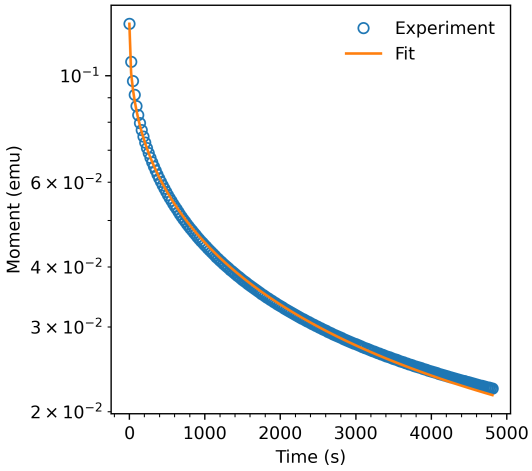

(d) 200 Oe

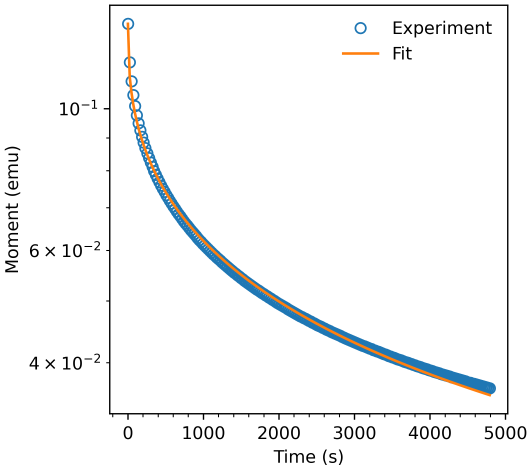

(e) 250 Oe

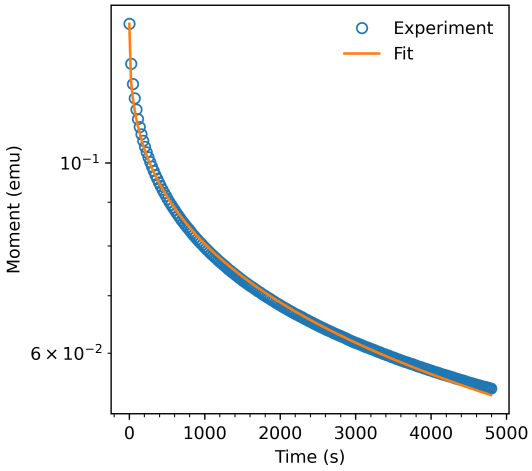

(f) 300 Oe

Figure S52: Infield DC Decay measurements for 100mM of $[Dy(Cp^{ttt})_2][B(C_6F_5)_4]$ dissolved in DFB performed at 1.8 K

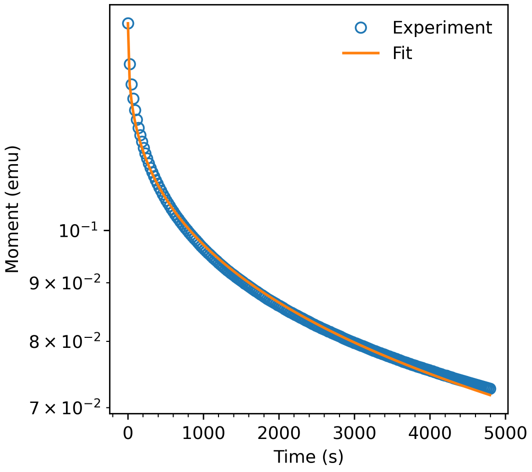

(a) 350 Oe

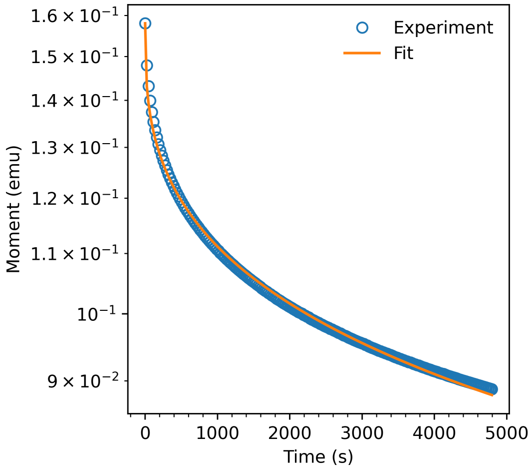

(b) 400 Oe

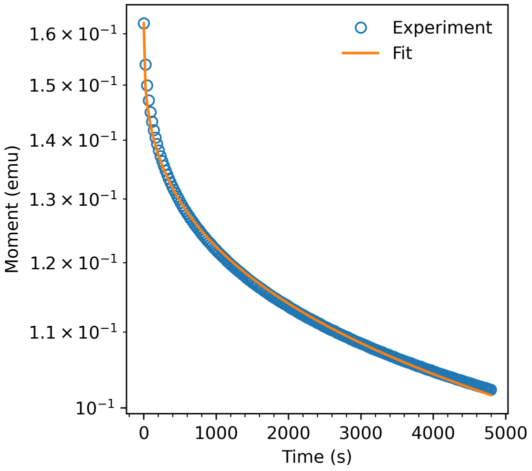

(c) 450 Oe

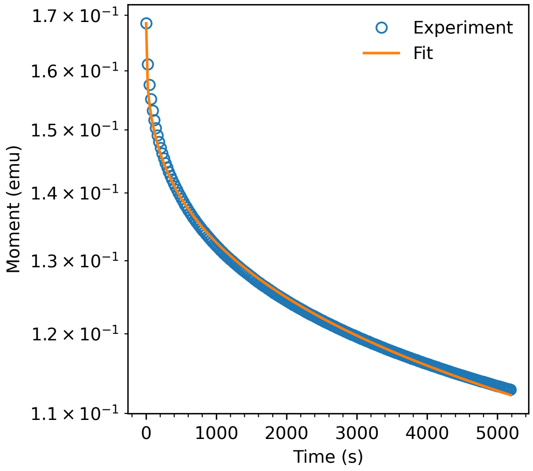

(d) 500 Oe

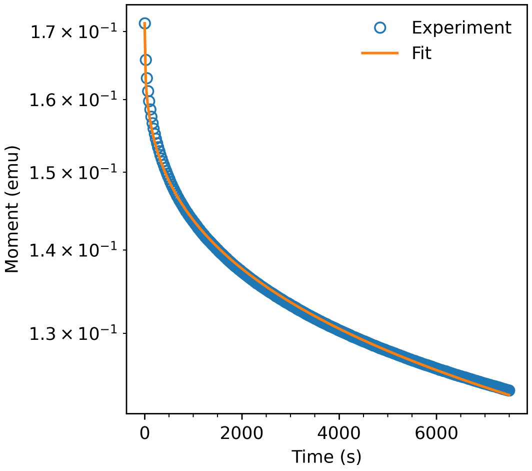

(e) 600 Oe

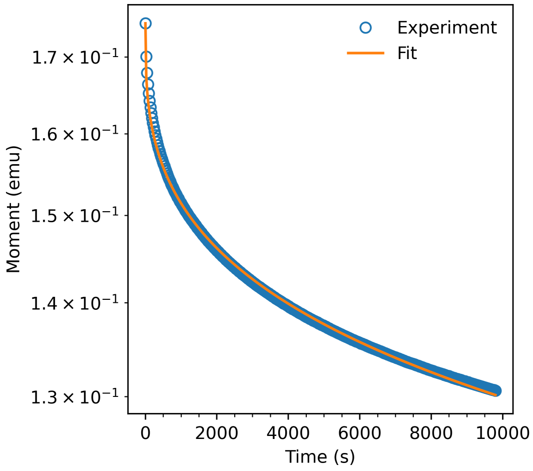

(f) 700 Oe

Figure S53: Infield DC Decay measurements for 100mM of $[Dy(Cp^{ttt})_2][B(C_6F_5)_4]$ dissolved in DFB performed at 1.8 K

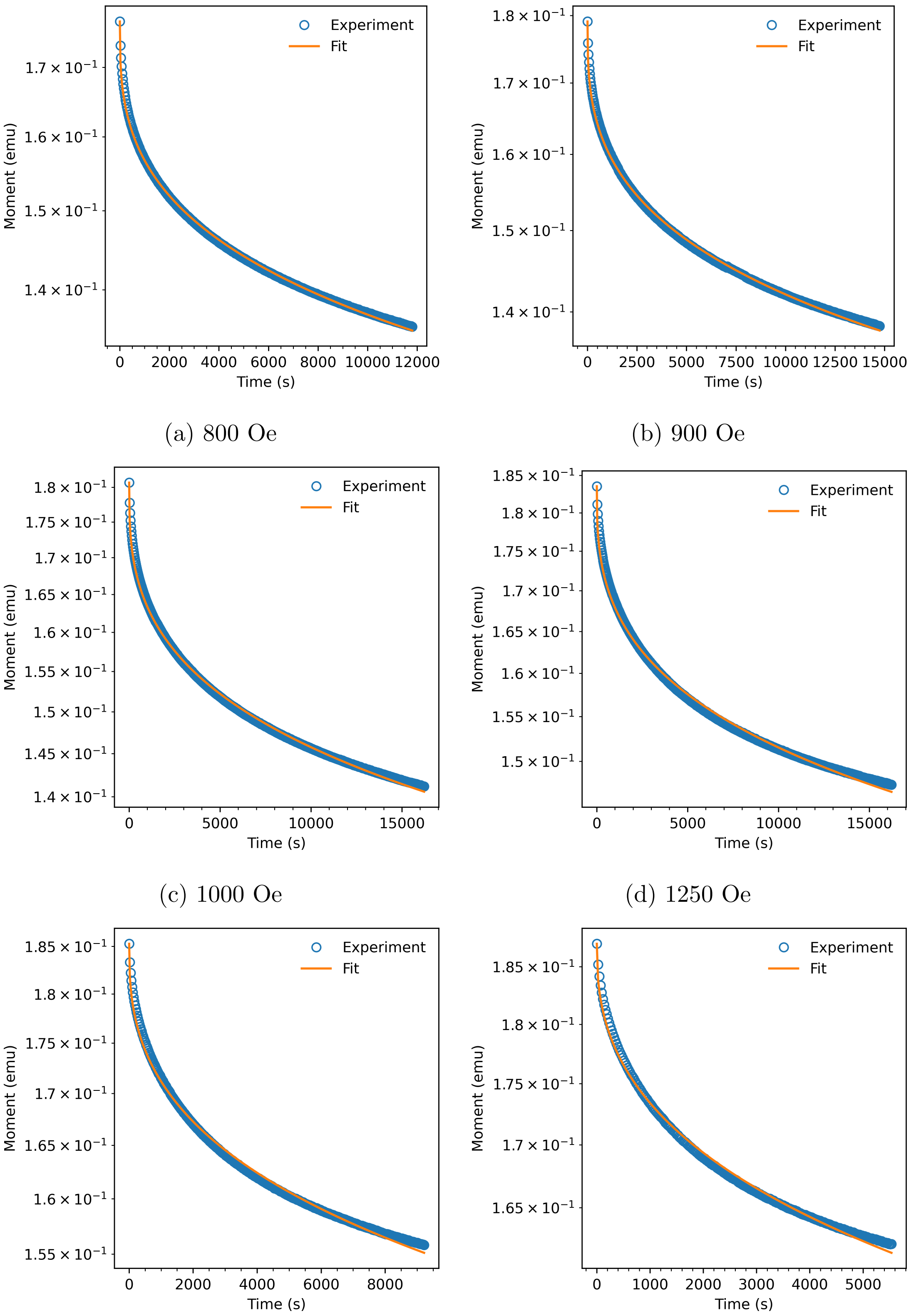

(a) 800 Oe

(b) 900 Oe

(c) 1000 Oe

(d) 1250 Oe

(e) 1500 Oe

(f) 1750 Oe

Figure S54: Infield DC Decay measurements for 100mM of $[Dy(Cp^{ttt})_2][B(C_6F_5)_4]$ dissolved in DFB performed at 1.8 K

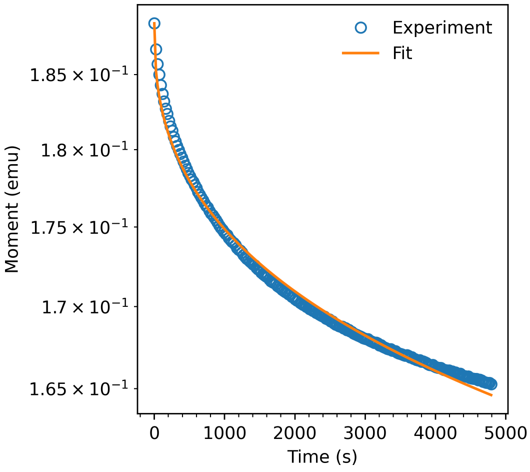

(a) 2000 Oe

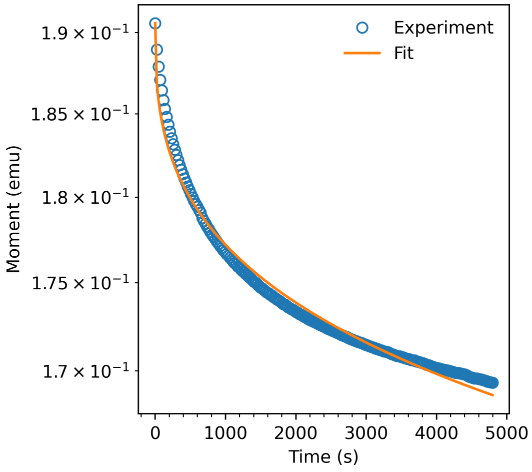

(b) 2500 Oe

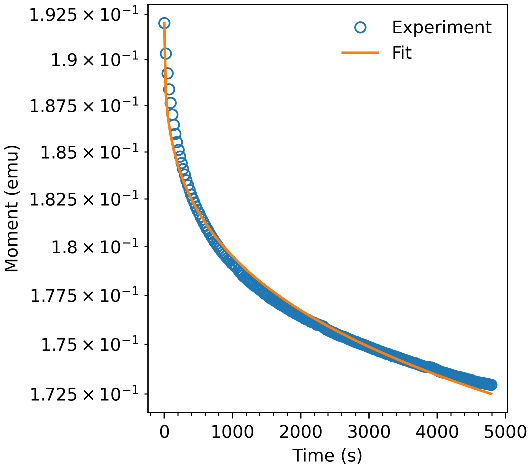

(c) 3000 Oe

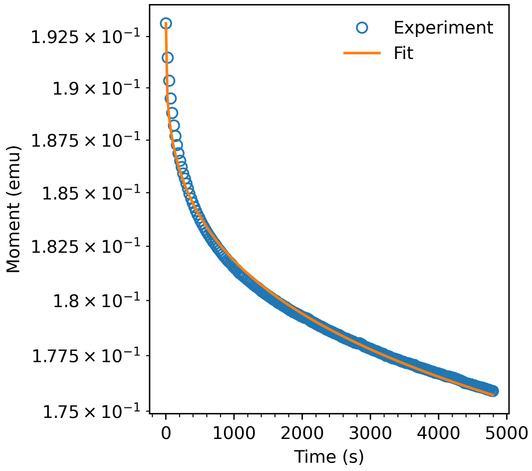

(d) 3500 Oe

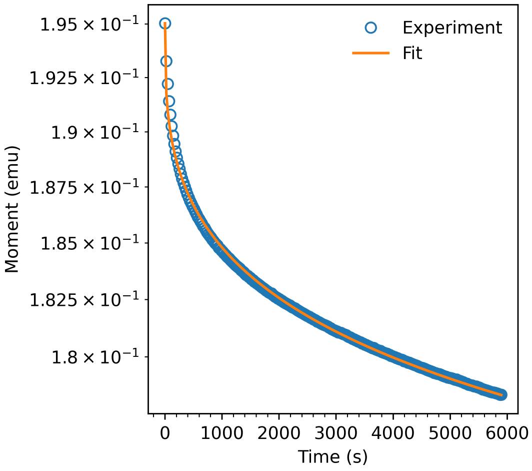

(e) 4000 Oe

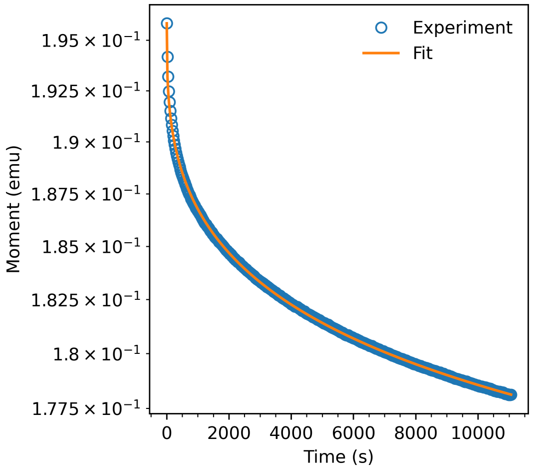

(f) 4500 Oe

Figure S55: Infield DC Decay measurements for 100mM of $[Dy(Cp^{ttt})_2][B(C_6F_5)_4]$ dissolved in DFB performed at 1.8 K

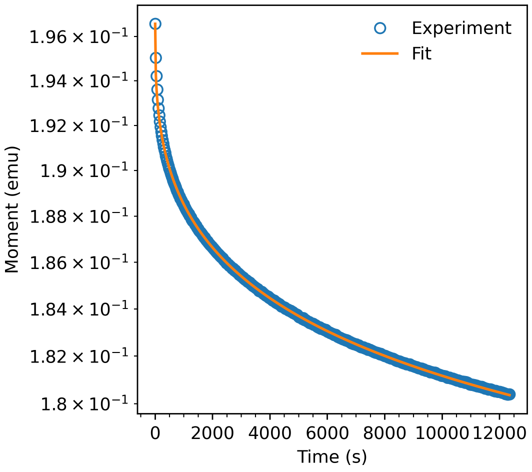

(a) 5000 Oe

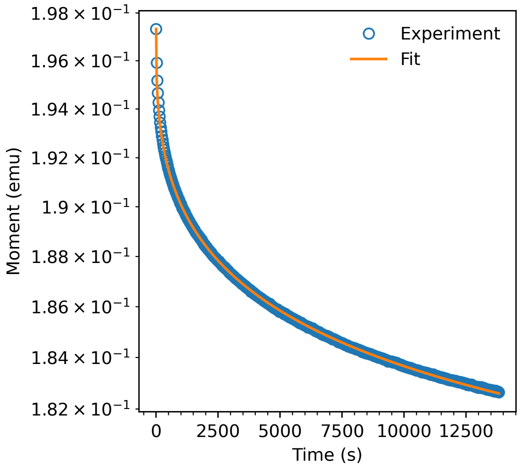

(b) 5500 Oe

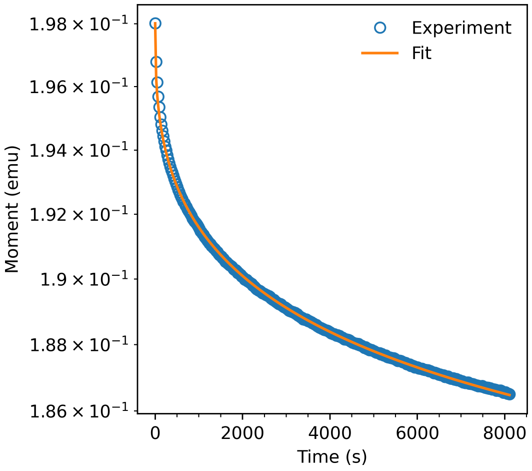

(c) 6000 Oe

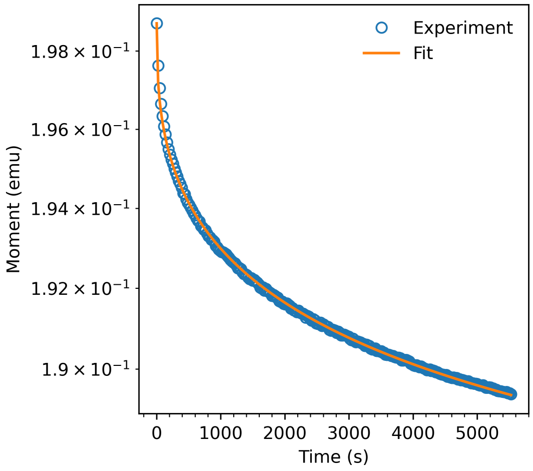

(d) 6500 Oe

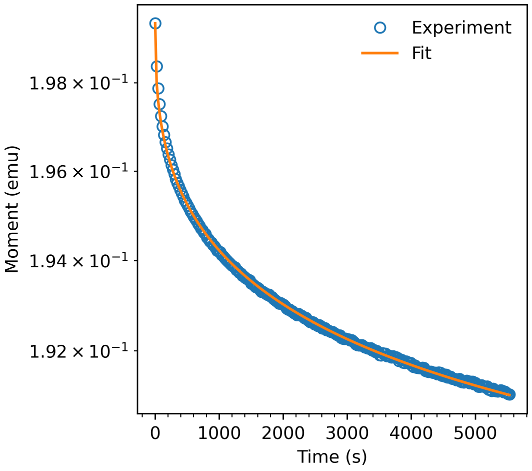

(e) 7000 Oe

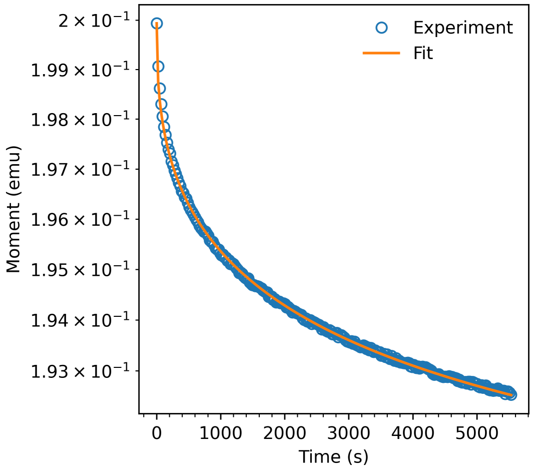

(f) 7500 Oe

Figure S56: Infield DC Decay measurements for 100mM of $[Dy(Cp^{ttt})_2][B(C_6F_5)_4]$ dissolved in DFB performed at 1.8 K

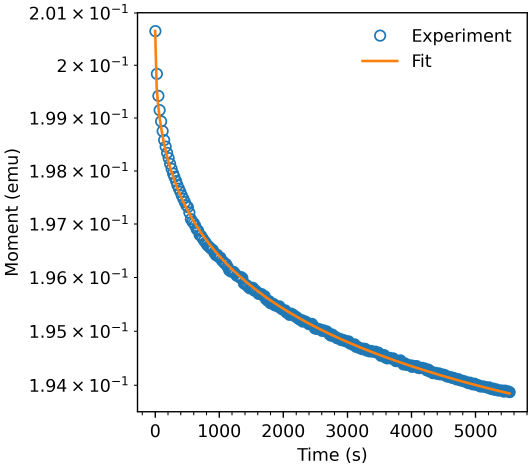

(a) 8000 Oe

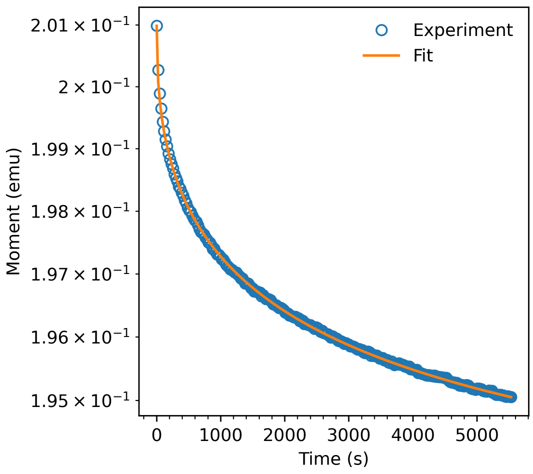

(b) 8500 Oe

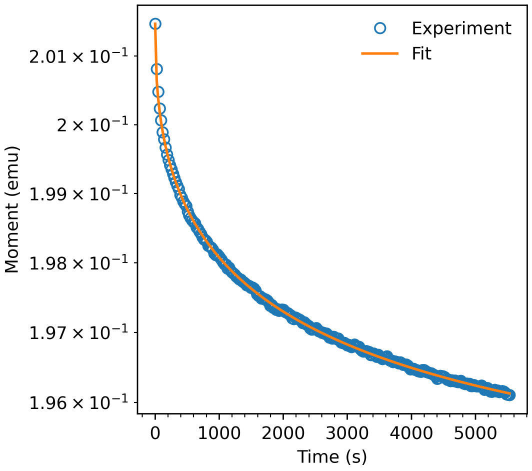

(c) 9000 Oe

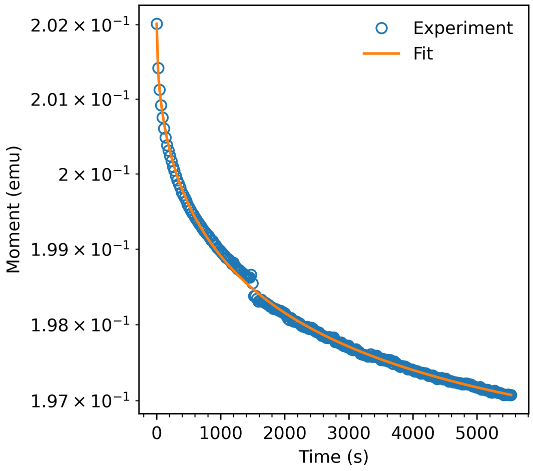

(d) 9500 Oe

Figure S57: Infield DC Decay measurements for 100mM of $[Dy(Cp^{ttt})_2][B(C_6F_5)_4]$ dissolved in DFB performed at 1.8 K

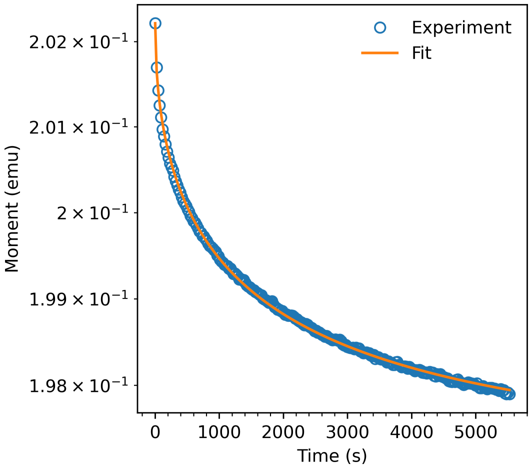

(a) 10000 Oe

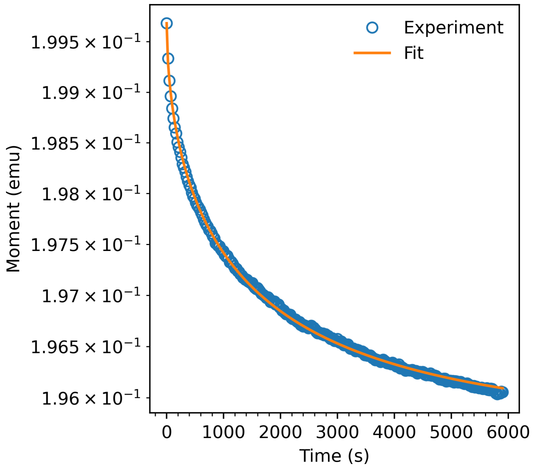

(b) 10500 Oe

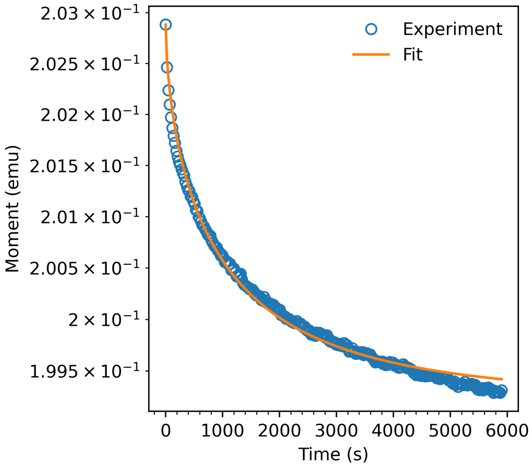

(c) 11000 Oe

Figure S58: Infield DC Decay measurements for 100mM of $[Dy(Cp^{ttt})_2][B(C_6F_5)_4]$ dissolved in DFB performed at 1.8 K

10mM

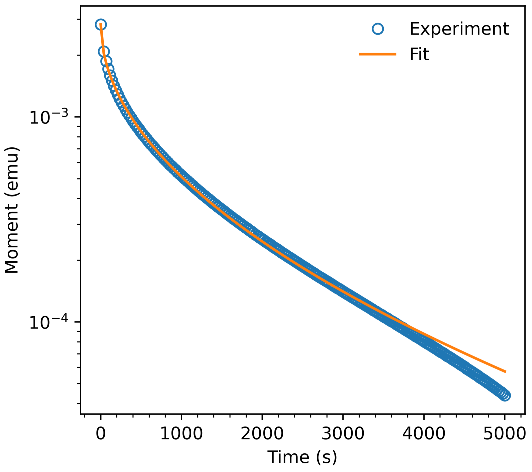

(a) 0 Oe

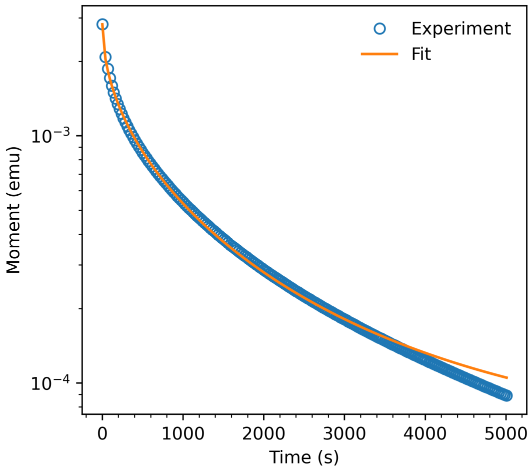

(b) 10 Oe

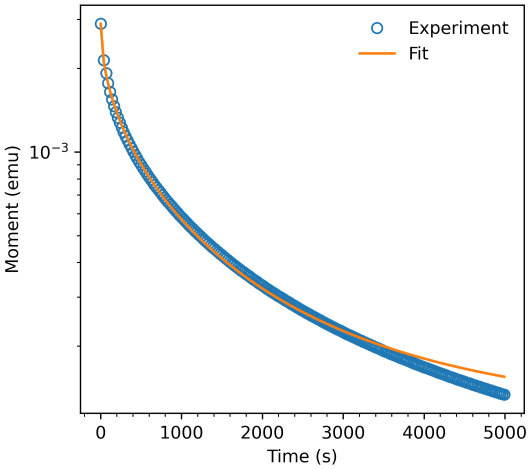

(c) 20 Oe

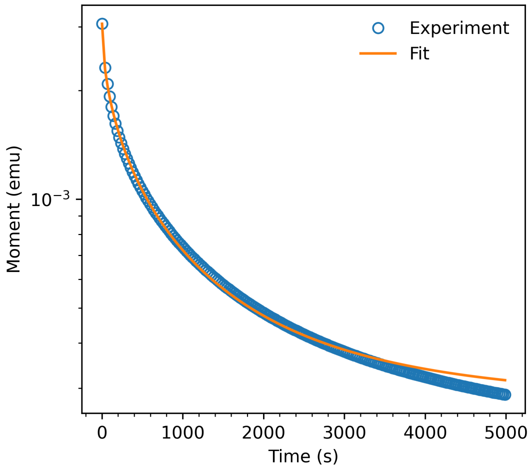

(d) 50 Oe

Figure S59: Infield DC Decay measurements for 10mM of $[Dy(Cp^{ttt})_2][B(C_6F_5)_4]$ dissolved in DFB performed at 1.8 K

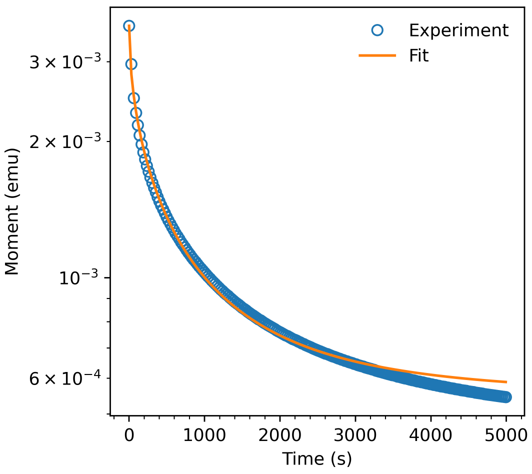

(a) 100 Oe

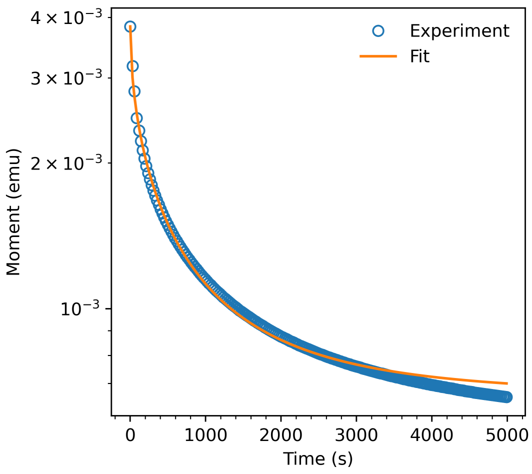

(b) 120 Oe

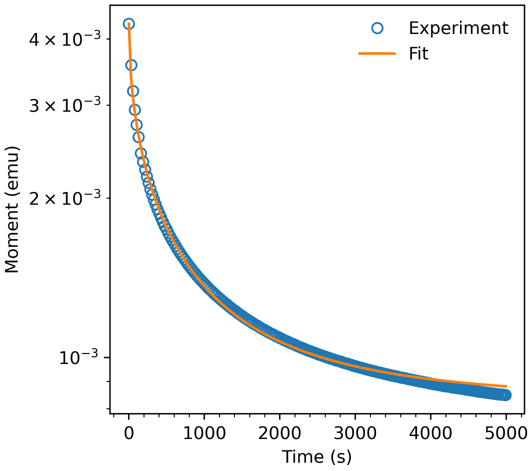

(c) 150 Oe

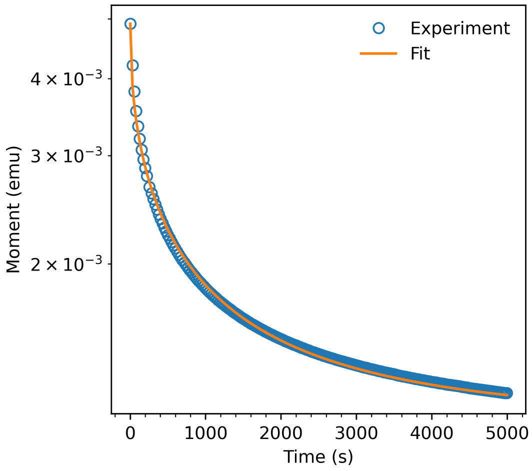

(d) 200 Oe

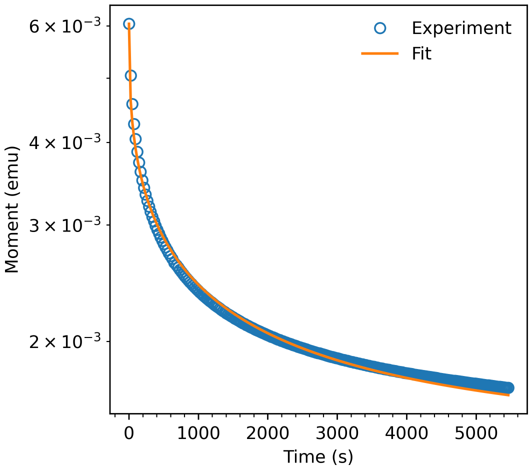

(e) 250 Oe

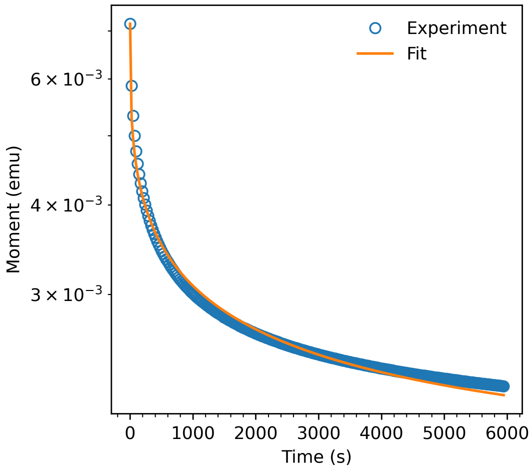

(f) 300 Oe

Figure S60: Infield DC Decay measurements for 10mM of $[Dy(Cp^{ttt})_2][B(C_6F_5)_4]$ dissolved in DFB performed at 1.8 K

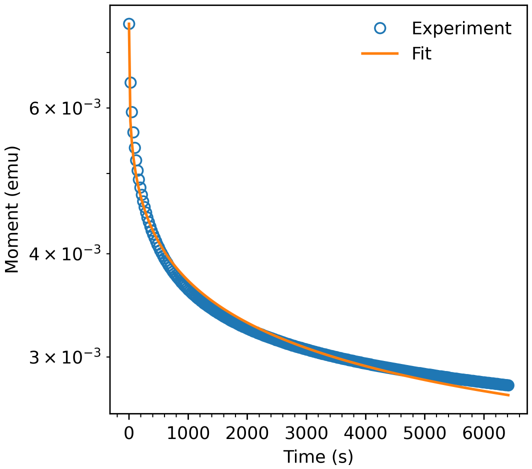

(a) 350 Oe

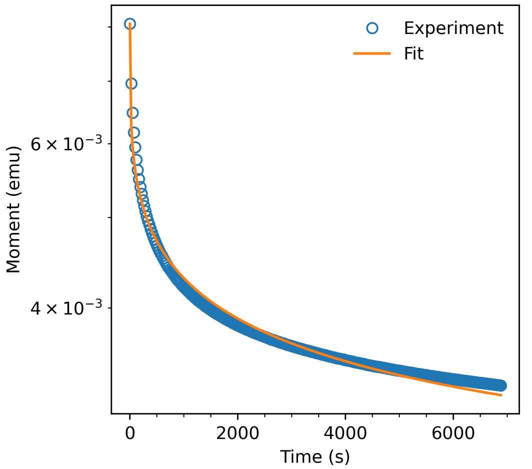

(b) 400 Oe

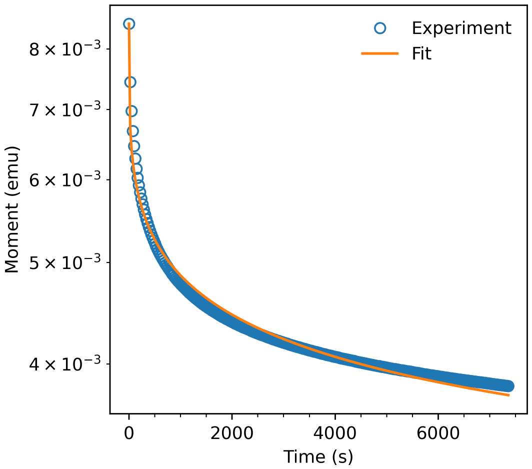

(c) 450 Oe

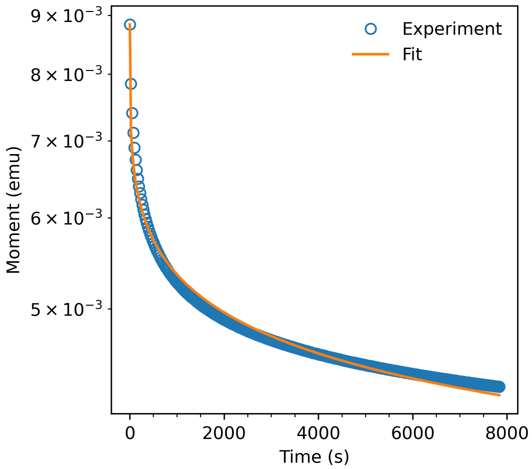

(d) 500 Oe

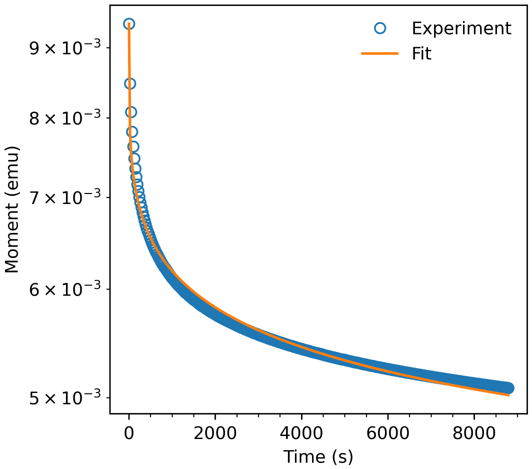

(e) 600 Oe

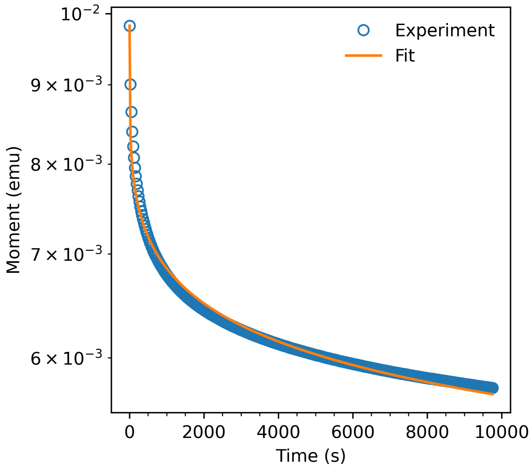

(f) 700 Oe

Figure S61: Infield DC Decay measurements for 10mM of $[Dy(Cp^{ttt})_2][B(C_6F_5)_4]$ dissolved in DFB performed at 1.8 K

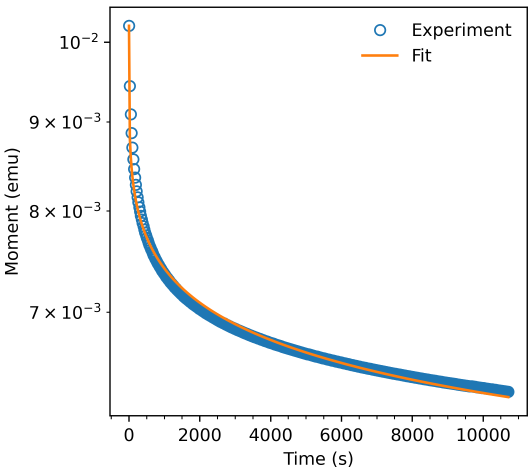

(a) 800 Oe

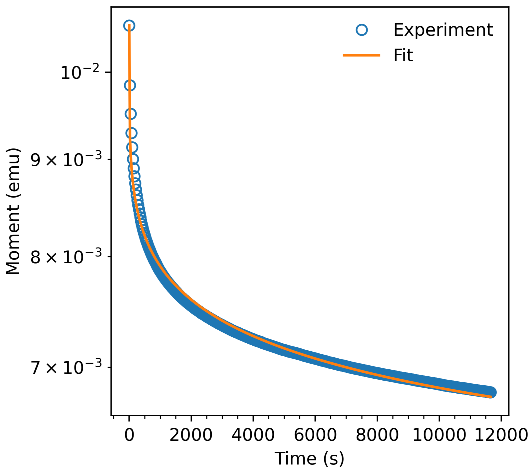

(b) 900 Oe

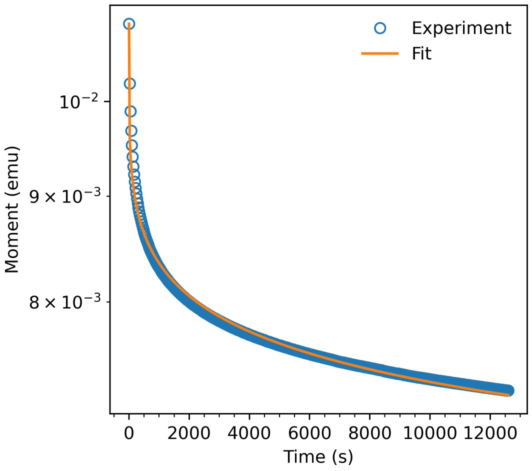

(c) 1000 Oe

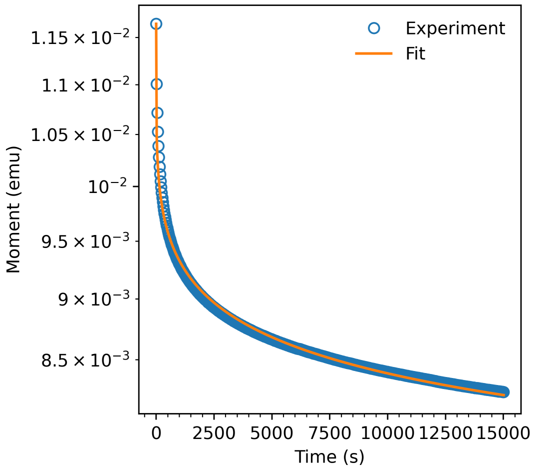

(d) 1250 Oe

Figure S62: Infield DC Decay measurements for 10mM of $[Dy(Cp^{ttt})_2][B(C_6F_5)_4]$ dissolved in DFB performed at 1.8 K

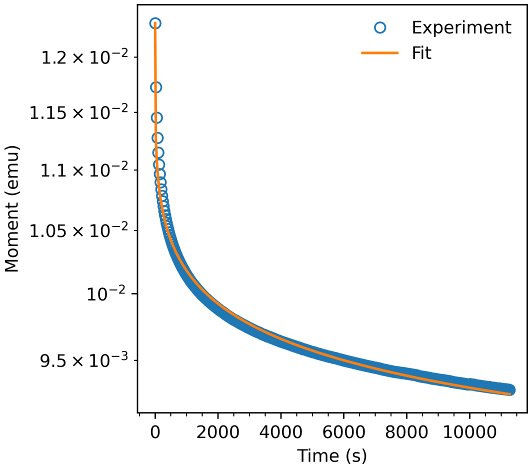

(a) 1500 Oe

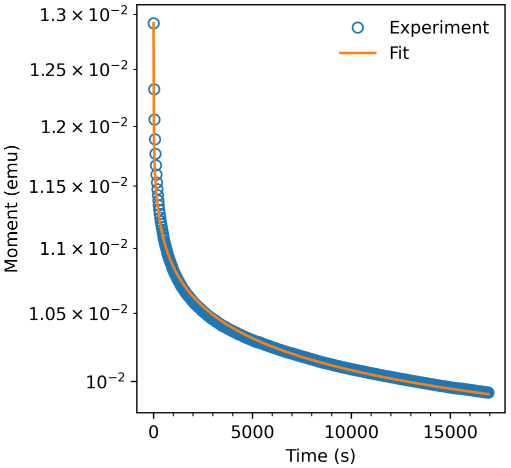

(b) 1750 Oe

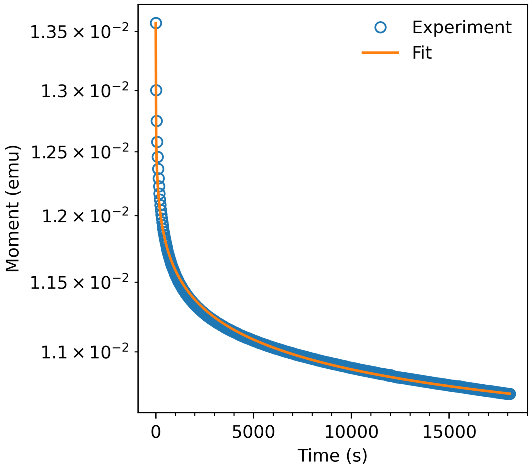

(c) 2000 Oe

Figure S63: Infield DC Decay measurements for 10mM of $[Dy(Cp^{ttt})_2][B(C_6F_5)_4]$ dissolved in DFB performed at 1.8 K

DCM

200mM

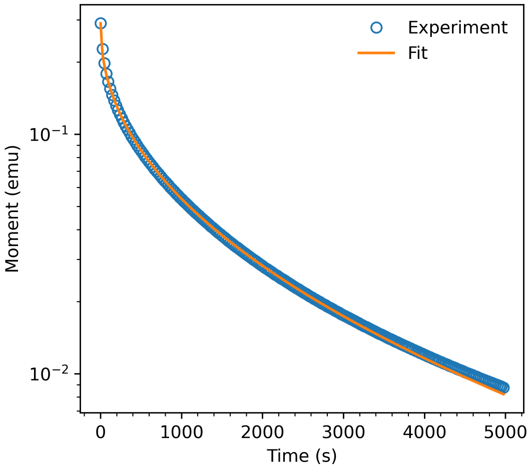

(a) 0 Oe

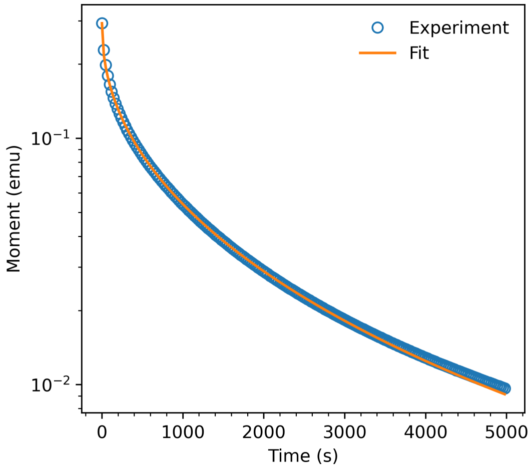

(b) 10 Oe

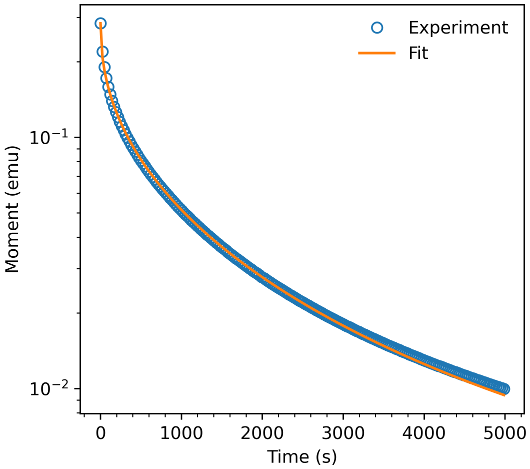

(c) 20 Oe

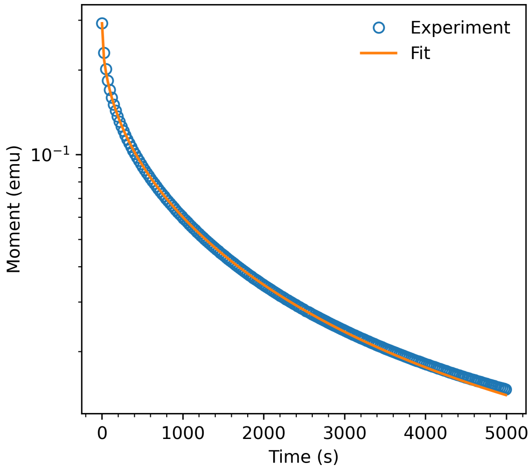

(d) 50 Oe

Figure S64: Infield DC Decay measurements for 200mM of $[Dy(Cp^{ttt})_2][B(C_6F_5)_4]$ dissolved in DCM performed at 1.8 K

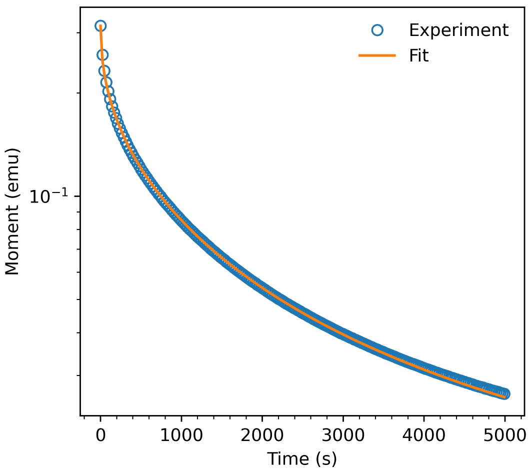

(a) 100 Oe

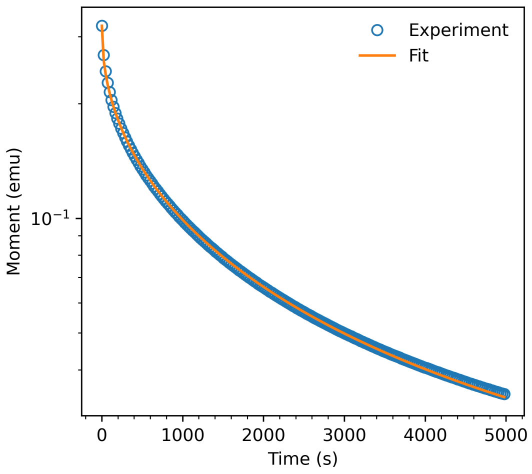

(b) 120 Oe

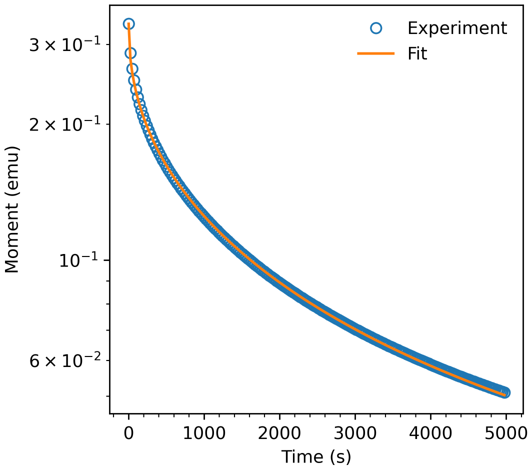

(c) 150 Oe

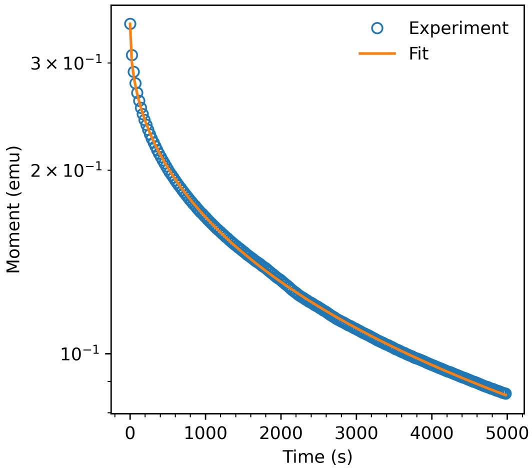

(d) 200 Oe

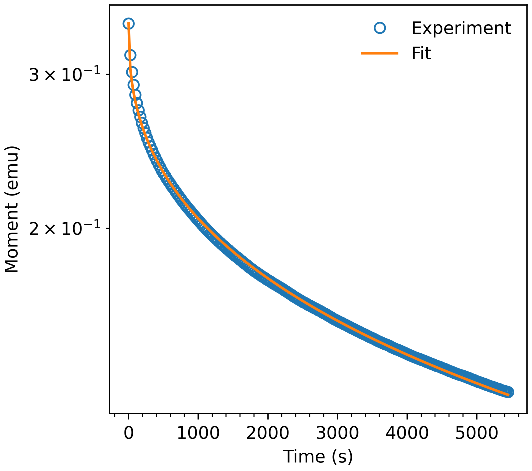

(e) 250 Oe

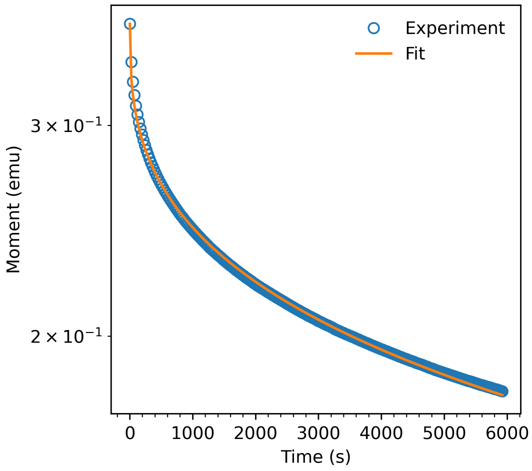

(f) 300 Oe

Figure S65: Infield DC Decay measurements for 200mM of $[Dy(Cp^{ttt})_2][B(C_6F_5)_4]$ dissolved in DCM performed at 1.8 K

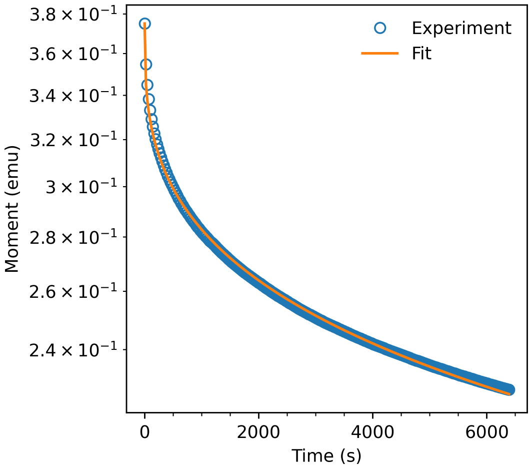

(a) 350 Oe

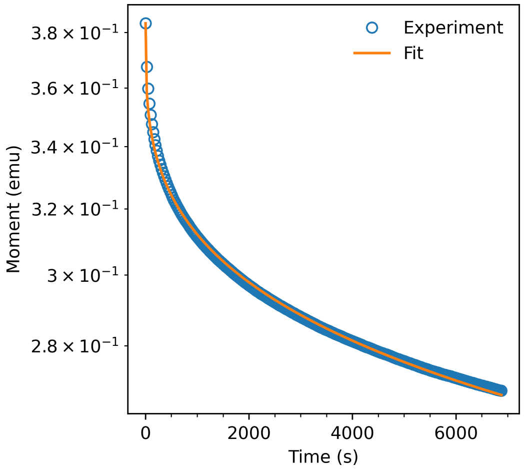

(b) 400 Oe

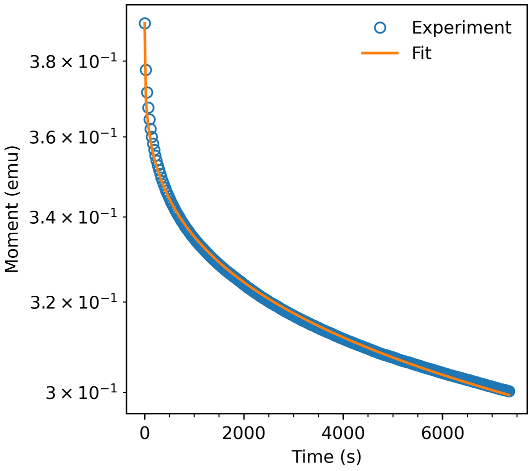

(c) 450 Oe

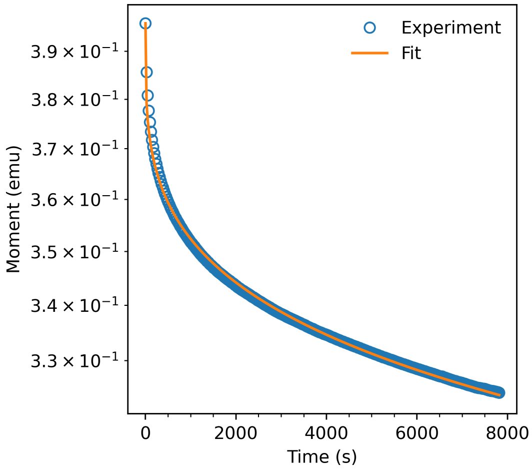

(d) 500 Oe

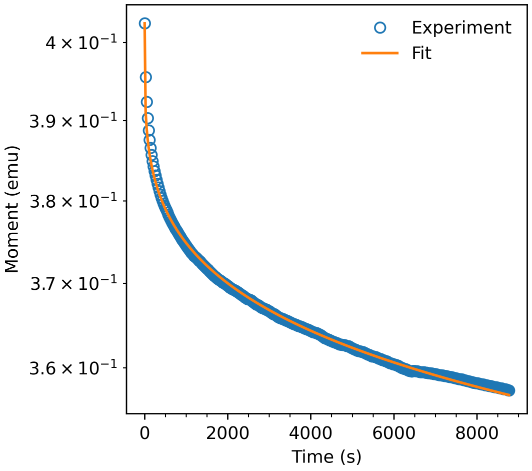

(e) 600 Oe

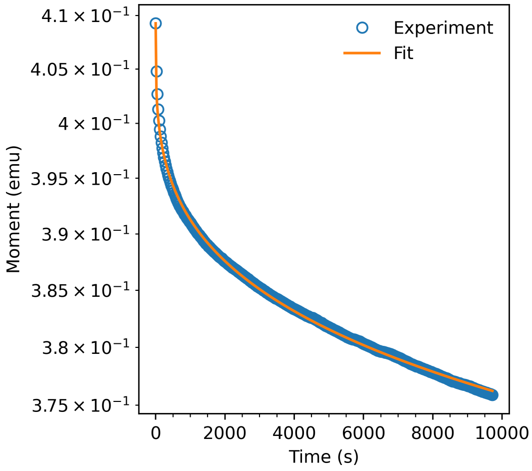

(f) 700 Oe

Figure S66: Infield DC Decay measurements for 200mM of $[Dy(Cp^{ttt})_2][B(C_6F_5)_4]$ dissolved in DCM performed at 1.8 K

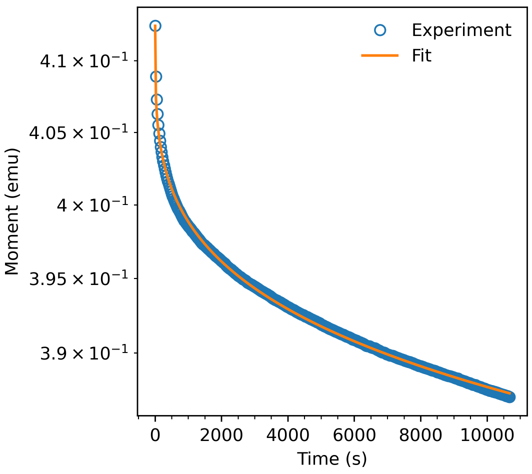

(a) 800 Oe

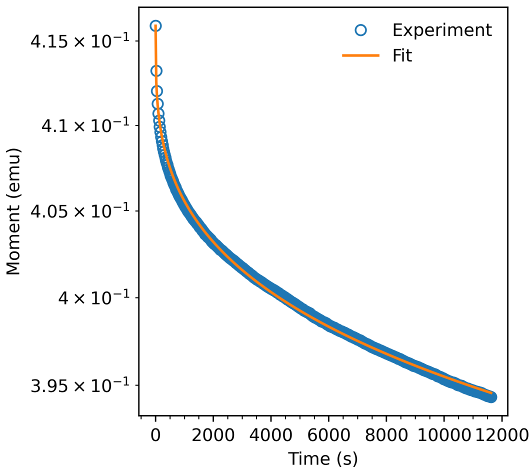

(b) 900 Oe

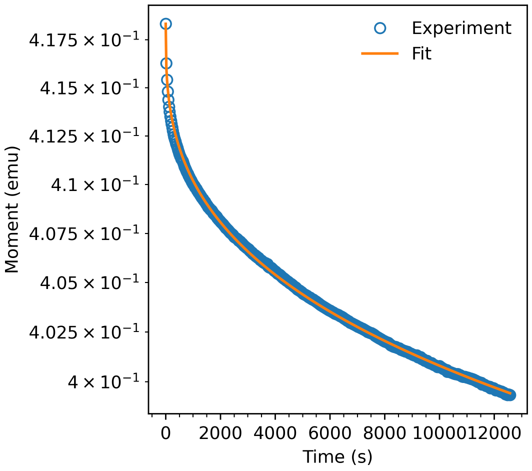

(c) 1000 Oe

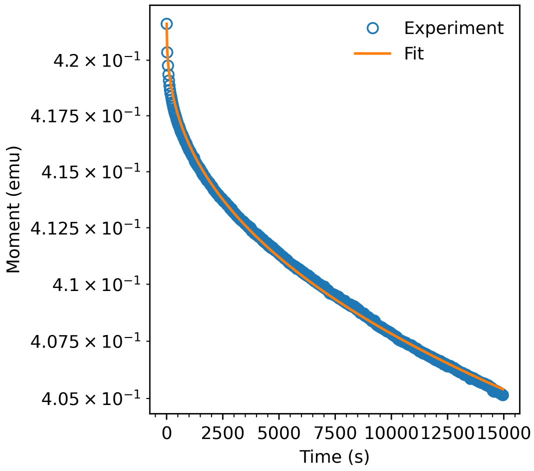

(d) 1250 Oe

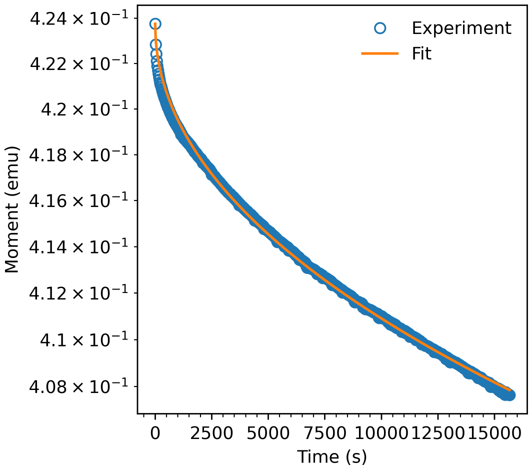

(e) 1500 Oe

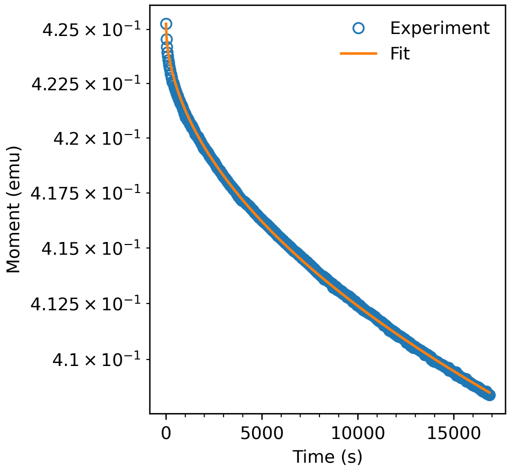

(f) 1750 Oe

Figure S67: Infield DC Decay measurements for 200mM of $[Dy(Cp^{ttt})_2][B(C_6F_5)_4]$ dissolved in DCM performed at 1.8 K

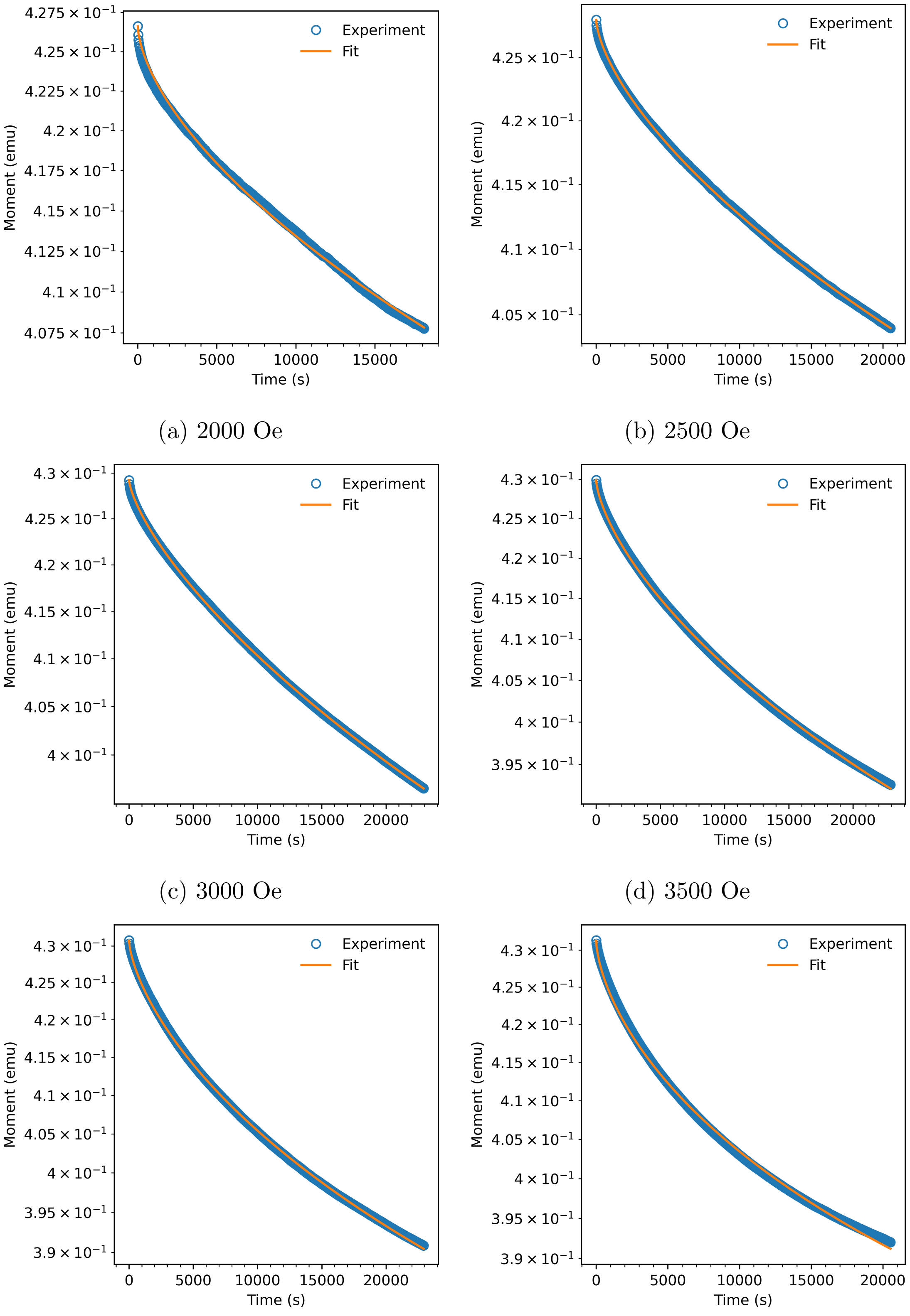

(a) 2000 Oe

(b) 2500 Oe

(c) 3000 Oe

(d) 3500 Oe

(e) 4000 Oe

(f) 4500 Oe

Figure S68: Infield DC Decay measurements for 200mM of $[Dy(Cp^{ttt})_2][B(C_6F_5)_4]$ dissolved in DCM performed at 1.8 K

100mM

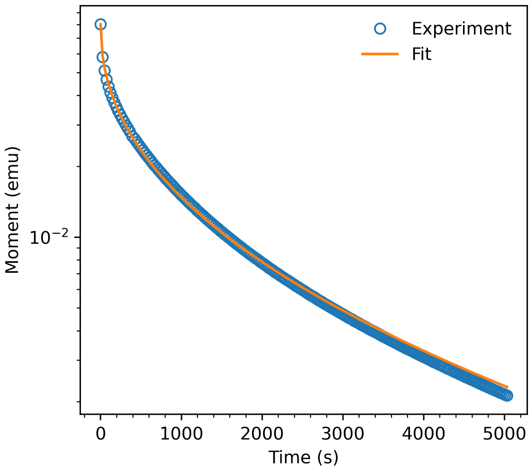

(a) 0 Oe

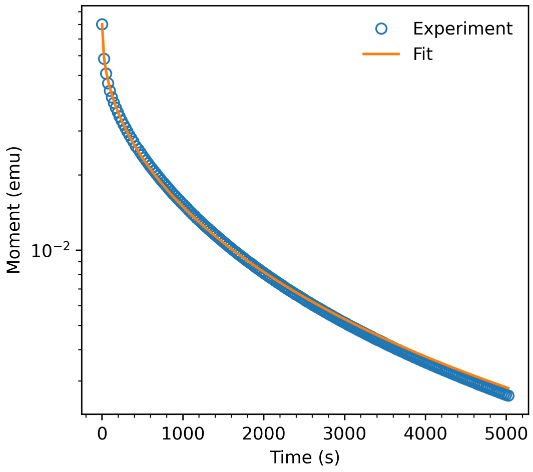

(b) 10 Oe

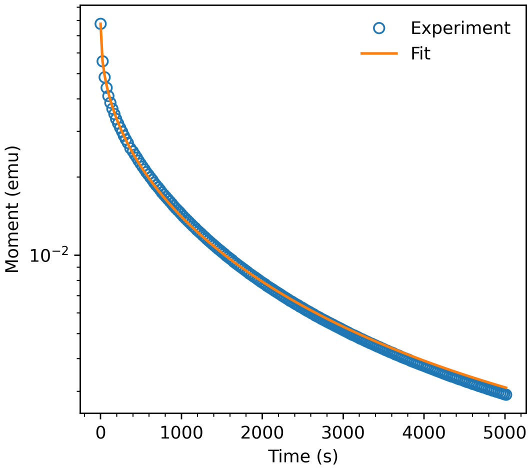

(c) 20 Oe

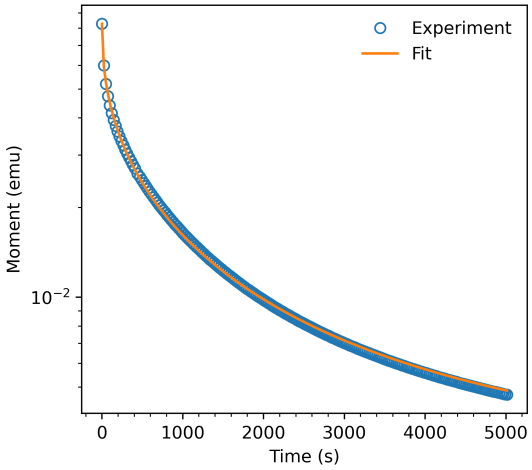

(d) 50 Oe

Figure S69: Infield DC Decay measurements for 100mM of $[Dy(Cp^{ttt})_2][B(C_6F_5)_4]$ dissolved in DCM performed at 1.8 K

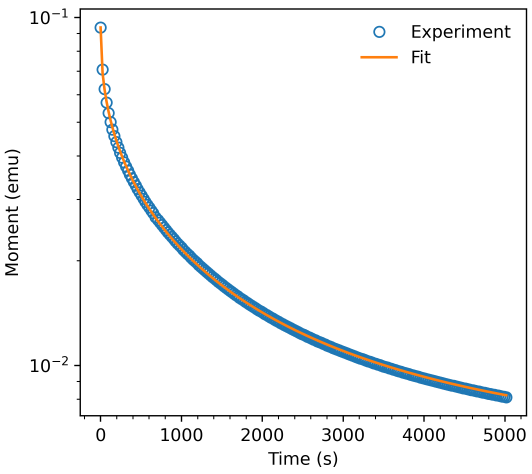

(a) 100 Oe

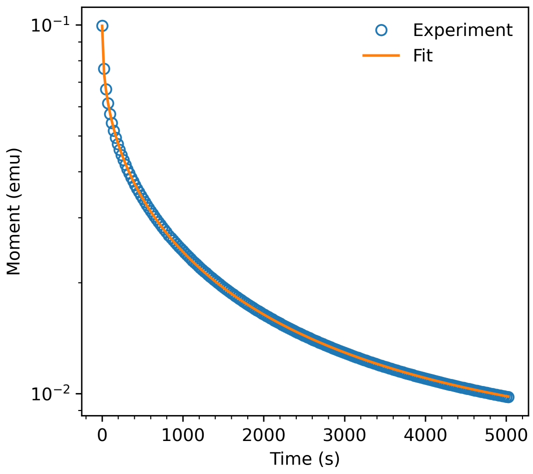

(b) 120 Oe

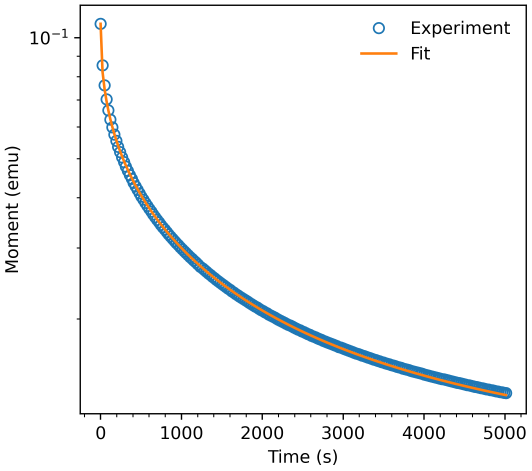

(c) 150 Oe

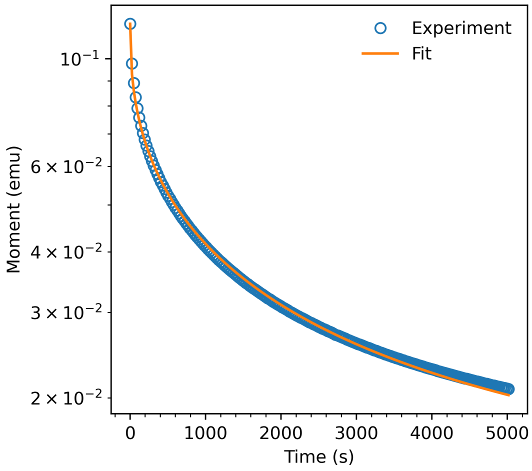

(d) 200 Oe

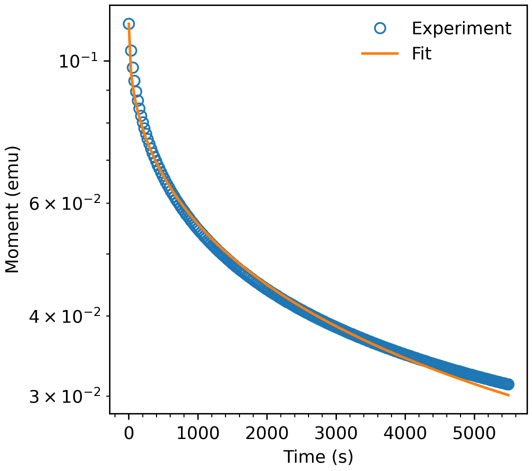

(e) 250 Oe

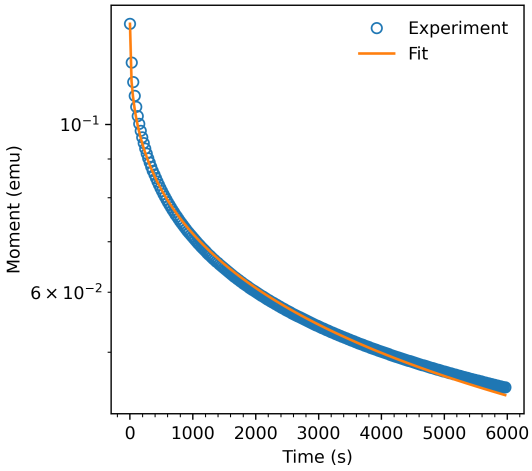

(f) 300 Oe

Figure S70: Infield DC Decay measurements for 100mM of $[Dy(Cp^{ttt})_2][B(C_6F_5)_4]$ dissolved in DCM performed at 1.8 K

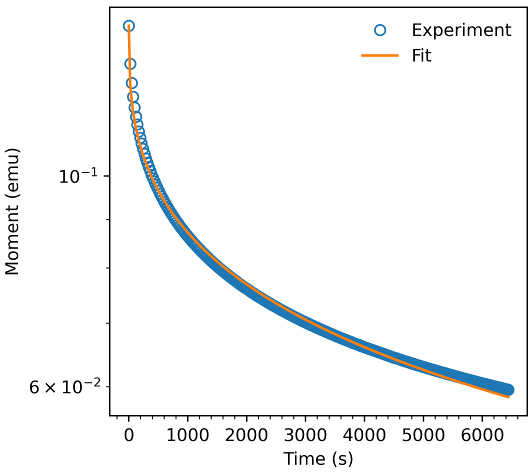

(a) 350 Oe

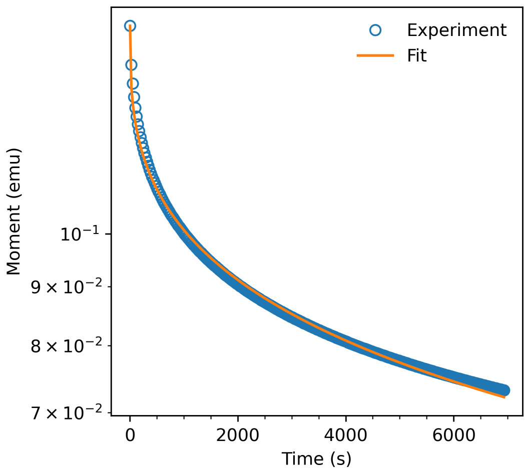

(b) 400 Oe

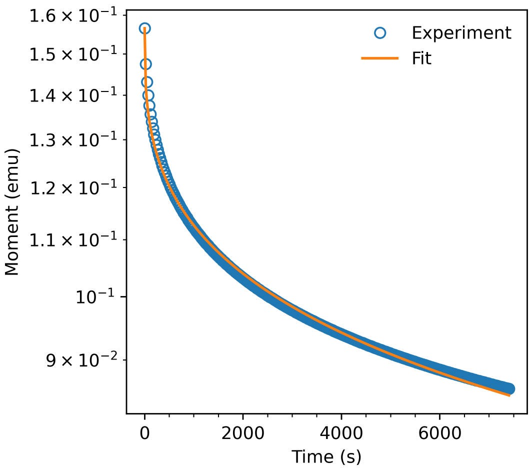

(c) 450 Oe

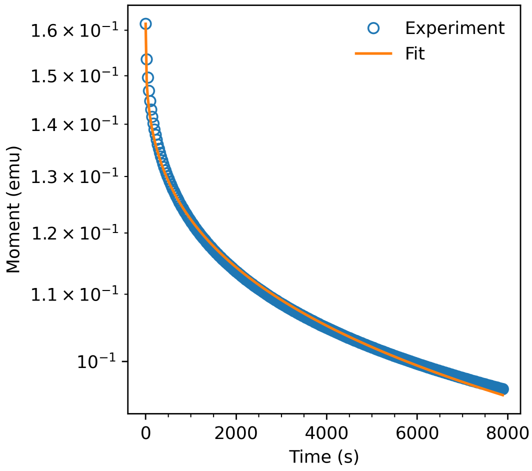

(d) 500 Oe

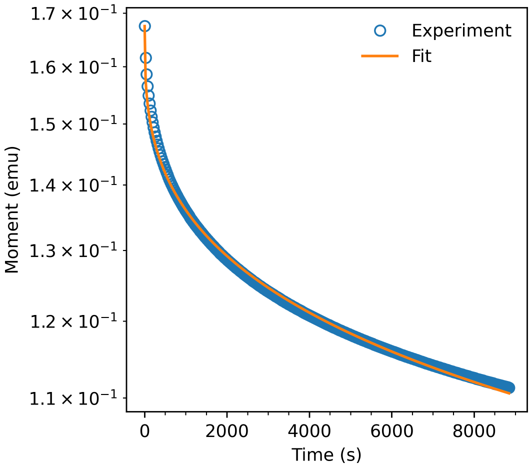

(e) 600 Oe

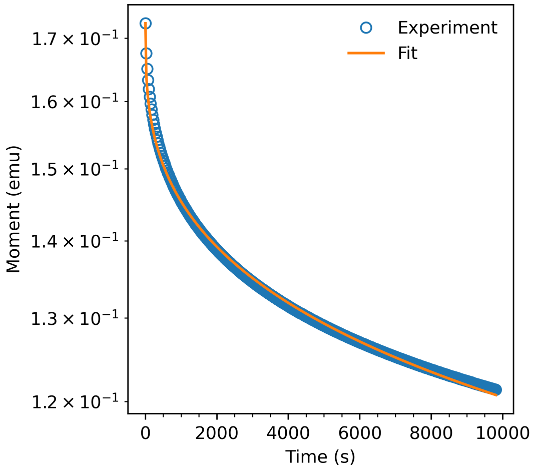

(f) 700 Oe

Figure S71: Infield DC Decay measurements for 100mM of $[Dy(Cp^{ttt})_2][B(C_6F_5)_4]$ dissolved in DCM performed at 1.8 K

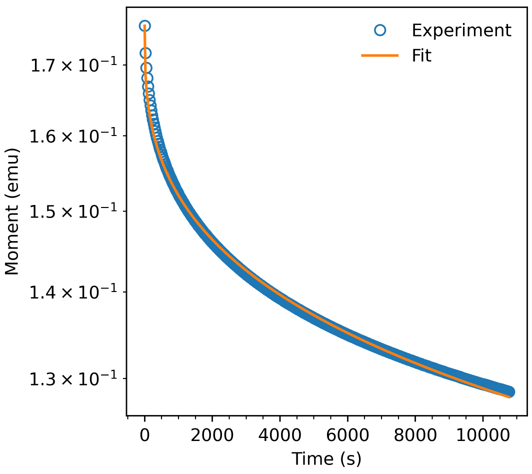

(a) 800 Oe

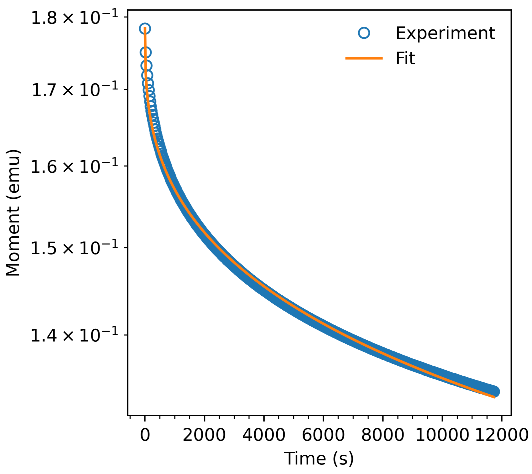

(b) 900 Oe

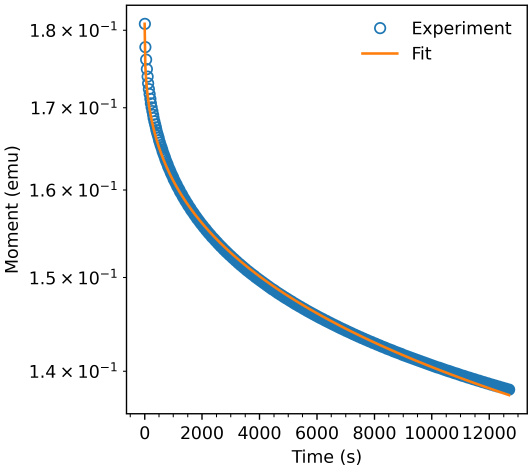

(c) 1000 Oe

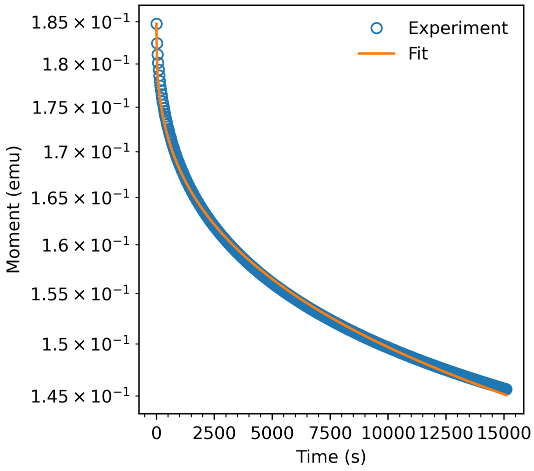

(d) 1250 Oe

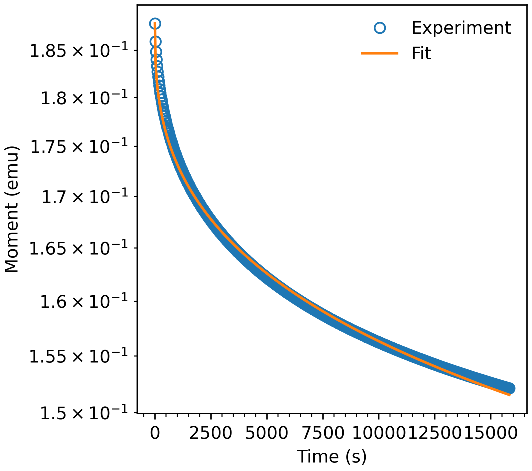

(e) 1500 Oe

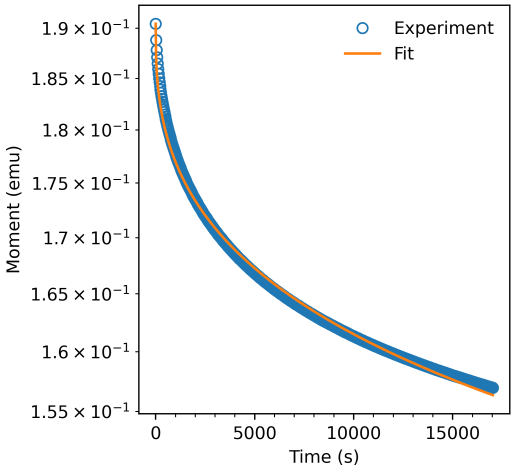

(f) 1750 Oe

Figure S72: Infield DC Decay measurements for 100mM of $[Dy(Cp^{ttt})_2][B(C_6F_5)_4]$ dissolved in DCM performed at 1.8 K

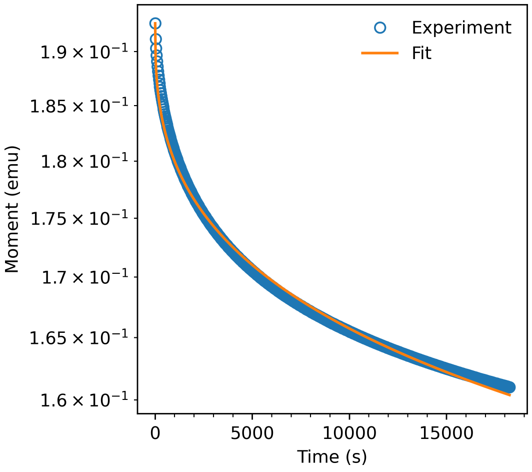

(a) 2000 Oe

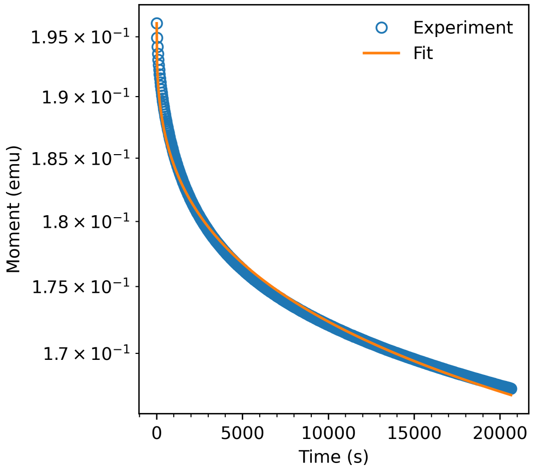

(b) 2500 Oe

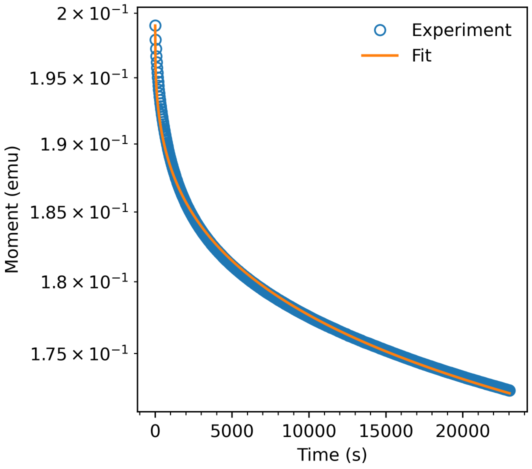

(c) 3000 Oe

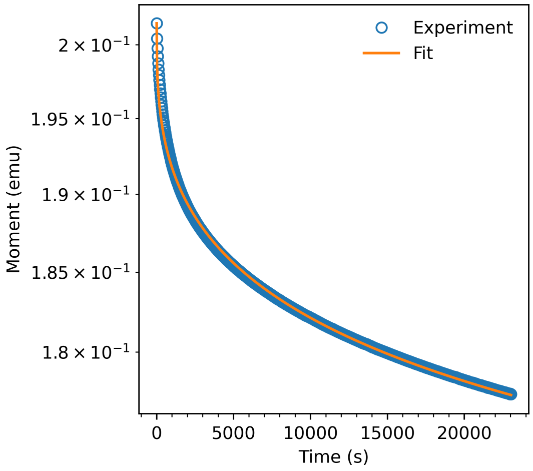

(d) 3500 Oe

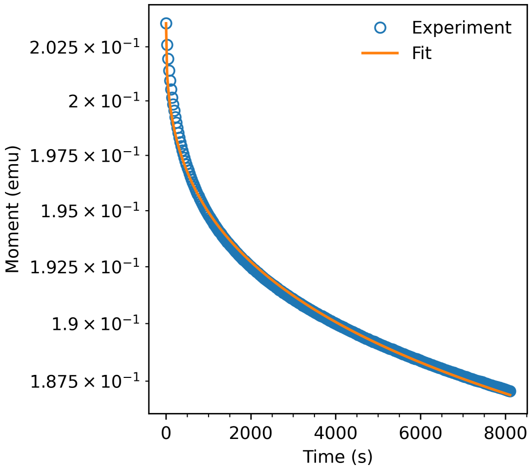

(e) 4000 Oe

Figure S73: Infield DC Decay measurements for 100mM of $[Dy(Cp^{ttt})_2][B(C_6F_5)_4]$ dissolved in DCM performed at 1.8 K

References

[S1] Blackmore, W. J.; Gransbury, G. K.; Evans, P.; Kragskow, J. G.; Mills, D. P.; Chilton, N. F. Characterisation of magnetic relaxation on extremely long timescales. *Physical Chemistry Chemical Physics* **2023**, *25*, 16735–16744.

[S2] Goodwin, C. A. P.; Ortu, F.; Reta, D.; Chilton, N. F.; Mills, D. P. Molecular magnetic hysteresis at 60 kelvin in dysprosocenium. *Nature* **2017**, *548*, 439–442.
